# Oversecretion of CCL3 by Irradiation-Induced Senescent Osteocytes Mediates Bone Homeostasis Imbalance

**DOI:** 10.3390/cells14040249

**Published:** 2025-02-10

**Authors:** Fanyu Zhao, Haiqing Han, Jing Wang, Jianping Wang, Jianglong Zhai, Guoying Zhu

**Affiliations:** Institute of Radiation Medicine, Fudan University, 2094 Xietu Road, Shanghai 200032, China; 22211140009@m.fudan.edu.cn (F.Z.); 23211140001@m.fudan.edu.cn (H.H.); 21211140004@m.fudan.edu.cn (J.W.); jianpingwang@fudan.edu.cn (J.W.); jlzhai@fudan.edu.cn (J.Z.)

**Keywords:** CC chemokine ligand 3, ionizing radiation, inflammation, osteocytes, cellular senescence, PI3K/Akt/NF-κB signaling pathway, bone homeostasis

## Abstract

Various stressors such as ionizing radiation (IR), chemotherapeutic agents, oxidative stress, and inflammatory responses can trigger the stress-induced premature senescence (SIPS) of cells in the bone microenvironment, including osteocytes. However, little is known about the mechanisms underlying the senescent cellular regulation of the differentiation potential and bone homeostasis. Here, we report a secretory change in senescent osteocytes activated by IR, its subsequent impact on osteogenic and osteoclastic differentiation, and the inflammatory cascade response. It was observed that osteocytes exhibited altered biological function, persistent and incomplete DNA damage repair, and characteristic senescence phenotypes after exposure to IR in vitro. Meanwhile, a concomitant increase in the CC chemokine ligand 3 (CCL3), a key component of the senescence-associated secretory phenotype (SASP), was observed in the IR-induced senescent osteocytes, which could further downregulate the osteogenic differentiation and enhance the osteoclastic differentiation in cell supernatant co-culture experiments. Notably, the enhancement of the PI3K/Akt/NF-κB signaling pathway in IR-induced senescent osteocytes appears to be an essential driver of the imbalance between the osteogenic and osteoclastic differentiation potentials. Taken together, these data suggest a novel role of CCL3 in IR-induced bone homeostatic imbalance through SASP cascade secretion, mediated by the PI3K/Akt/NF-κB signaling pathway.

## 1. Introduction

Radiotherapy is essential for cancer treatment to kill cancer cells and shrink tumors [1], while it remains an intractable challenge to eradicate tumors and reduce the side effects to healthy tissue. Bone tissue is often involved during local radiotherapy treatments because of its unique distribution in the body, leading to a deleterious effect on the bone metabolism and microstructure, which increases the risk of bone loss and pathological fractures, as well as tumor bone metastasis due to bone matrix damage [2,3,4,5]. However, the deleterious effects of irradiation on healthy bone tissue and the resulting radiation damage mechanisms are poorly understood. Furthermore, as survival rates improve and cancer patients live longer, the combination of therapy-induced senescence (TIS) and physiological senescence in tumor-prone age groups can exacerbate bone loss and structural damage in irradiated areas and even throughout the entire body [6,7,8].

Studies have shown that ionizing radiation (IR) directly affects cells’ activity and repopulation capacity, resulting in deteriorated bone formations with cell cycle arrest and direct cell death [9,10]. Meanwhile, IR can prompt oxidative stress, DNA damage, and cellular apoptosis [11], leading to a senescent state in most cell types within the bone microenvironment and to the development of a heterogeneous senescence-associated secretory phenotype (SASP) [12,13], commonly referred to as stress-induced premature senescence (SIPS) [7]. The pathological accumulation of senescent cells (SNCs) is implicated in the generation of a pro-inflammatory secretome or SASP, subsequently promoting secondary senescence and tissue homeostasis disruption [14,15,16]. The level of SASP relies on the positive feedback loop of autocrine and paracrine, resulting in a cascade amplification effect [13,15].

Osteocytes (OCYs), former osteoblasts that are encapsulated by a mineralized bone matrix, are multifunctional and dynamic cells that are capable of integrating hormonal and mechanical signals and transmitting them to effector cells in bone and other distant tissues. They also play a crucial role in maintaining skeletal homeostasis [17,18,19,20,21]. Senescent OCYs, as one of the main SASP-secreting cell types in the bone microenvironment [22], and diverse SASPs may contribute to senescence-related bone matrix disintegration and bone loss [23]. Studies have revealed that aging is linked to OCY dysfunction, with degeneration being observed in the OCY lacuna-canalicular network in both older adults and aging animal models [24,25]. Furthermore, the accumulation of senescent OCYs can alter mesenchymal lineage commitment, subsequently inhibiting osteogenesis and promoting osteoclastogenesis, and ultimately leading to a bone homeostasis imbalance [26,27,28]. These findings have revealed that increased senescent OCYs and their SASPs could be key etiological factors of the SIPS and bone damage associated with natural aging and premature aging syndromes, including radiotherapy, chemotherapy, and metabolic dysfunction, where they appear to mediate skeletal deterioration [12]. It is worth noting that certain pivotal SASP factors can participate in an autocrine feedback loop, further amplifying the SASP signal [29].

These SASP factors, which are heterogeneous and tissue-specific, mainly include proteins (such as interleukins, chemokines, and growth factors), bioactive lipids, small extracellular vesicles, and non-coding nucleic acids that can lead to multiple senescence-associated disorders by eliciting local and systemic consequences [30]. As one of the key SASP factors that are secreted by senescent OCYs, CC chemokine ligand 3 (CCL3), initially known as macrophage inflammatory protein 1α (MIP-1α) [31], is a pro-inflammatory cytokine that can activate the chemotaxis of T cells, B cells, and monocytes [32]. It is also involved in the inflammatory process [33] and the modulation of bone homeostasis. Firstly, CCL3 can inhibit osteoblast proliferation and osteogenic differentiation by binding to CCR1 and downregulating the expression of osteocalcin (OCN) [34,35], as well as promoting adipogenic differentiation [35]. Additionally, as a potent osteoclast-activating factor (OAF) [36], CCL3 can regulate the recruitment, migration, adhesion, and differentiation of osteoclast precursors, thereby promoting osteoclast formation in a direct or indirect manner [37,38]. However, it is not fully understood whether CCL3 plays an important role in IR-induced bone homeostasis imbalance associated with oxidative stress and cellular senescence.

To understand how bone homeostasis is damaged by IR, it is necessary to use several types of analysis, considering changes in distinct cellular alterations and their heterogeneous SASP in the bone microenvironment. The aim of the present study was to evaluate the effects of IR on OCYs, with a specific focus on IR-induced cellular senescence, aberrant secretion of SASP factors, and subsequent activation of cytokine cascades. Furthermore, this study aimed to elucidate the role of CCL3 in IR-mediated bone homeostasis disruption through SASP-mediated signaling pathways, particularly the PI3K/Akt/NF-κB axis. This research provides novel insights regarding the pathogenesis of cancer-treatment-induced bone loss (CTIBL), while laying the groundwork and providing experimental evidence for future research to identify new targets to treat IR-induced bone homeostasis imbalance.

## 2. Materials and Methods

### 2.1. Primary OCY Isolation and Identification

Primary OCYs were isolated from the calvaria of specific pathogen-free male BALB/c mice, adopting the protocol standardized in our laboratory [39]. All animal procedures underwent ethical review and were sanctioned by the Experimental Animal Ethics Committee of School of Pharmacy Fudan University (approval code: 2024-02-FYS-ZGY-02, approved on 20 February 2024). In brief, six-week-old, male BALB/c mice (weighing 18–22 g) were purchased from Jihui Laboratory Animal Breeding Co. (Shanghai, China). The mice’s calvaria were aseptically extracted and fragmented, followed by sequential digestion using 0.25% Trypsin (25200072; Gibco, Eggenstein, Germany), 0.1% g/mL collagenase type II (C6885; Sigma-Aldrich, St. Louis, MO, USA), 0.05 mM EDTA (E1170; Solarbio, Beijing, China), and 0.1% g/mL collagenase type I (C0130; Sigma-Aldrich, St. Louis, MO, USA) at 37 °C. Subsequently, the bone fragments and supernatant were incubated in a humidified incubator at 37 °C and 5% CO_2_. After 7 days of culture in vitro, the dendritic OCYs around bone fragments were visualized and subsequently identified as passage 0. The following experiments were conducted by utilizing passage 2–3 cells.

For the identification of primary OCYs, the indexes of alkaline phosphatase (ALP) stain and E11/gp38 marker protein were utilized. During the ALP staining, the cells were washed with PBS after removing the medium, fixed with 2.5% glutaraldehyde for 5 min, rinsed with PBS, and then incubated at room temperature on a shaker with an ALP staining solution, provided in the BCIP/NBT Alkaline Phosphatase Color Development Kit (C3206; Beyotime Institute of Biotechnology, Shanghai, China), for 1 h. For each well, images of 10 random view fields were taken at 100× magnification under a light microscope (Leica Microsystems, Weltzar, Germany). Furthermore, the mRNA and protein expression levels of the OCY marker protein E11/gp38 were detected by means of RT-qPCR and Western blot analysis, respectively. Primary osteoblasts (OBs), isolated according to the protocol established in our laboratory [39], were detected simultaneously and utilized as controls. The detailed procedures of the RT-qPCR and Western blot analysis are described below.

### 2.2. Cell Culture and Irradiation

The cells were cultured in a humidified environment containing 5% CO_2_ at 37 °C. The culture medium consisted of an α-minimum essential medium (α-MEM) (C12571500BT; Gibco, Eggenstein, Germany) supplemented with 10% fetal bovine serum (FBS) (10099-141C; Gibco, Eggenstein, Germany), 100 μg/mL of penicillin-streptomycin (PS) (15140122; Gibco-Thermo Fisher, Waltham, MA, USA), and 5 μg/mL of plasmocin^TM^ prophylactic (ant-mpp; Invivogen, San Diego, CA, USA). Once 30–40% confluence was reached after 24 h of culture, the cells were irradiated with 2 Gy of X-ray using an X-Rad 320 Biological Irradiator (X-RAD320; PXi, Houston, TX, USA). The irradiation parameters were set as follows: a tube voltage of 320 kV, tube current of 4.3 mA, source surface distance of 50.0 cm, and dose rate of 106.3 cGy/min. The control group was placed on the irradiator platform for an equivalent duration, but without undergoing irradiation treatment.

For experiments involving CCL3-neutralizing antibody treatment (AF-450; R&D Systems, Inc., Minneapolis, MN, USA), the irradiated OCYs were treated with 50 ng/mL of the antibody. The culture persisted for 72 h, with the culture medium being replaced every 2 days, and the neutralizing antibody was replenished every time the culture medium was changed.

### 2.3. IR-Induced Morphological Changes and Biological Impairments of OCYs In Vitro

#### 2.3.1. Cell Counting Kit-8 (CCK8) Cell Viability Assay

The cell viability of the irradiated OCYs was assessed using a CCK8 assay. In brief, OCYs were seeded into 96-well plates (3 × 10^3^ cells/well) and incubated for 24 h, then irradiated with 2 Gy of X-ray, followed by incubation for 1, 2, or 3 days. In parallel, OCYs were also irradiated at different levels of X-ray (0, 0.25, 0.5, 1, 2 Gy) to explore the dose-dependent effects of radiation on the cell viability. Following the incubation period, a CCK8 reagent (CK04; Dojindo, Kumamoto, Japan) was added to each well at a 10% concentration, and the plates were further incubated for 2 h at 37 °C. Finally, cell viability was determined by measuring absorbance at 450 nm using a microplate reader (Epoch™; Biotech, Vicenza, Italy).

#### 2.3.2. 5-Ethynyl-2′-deoxyuridine (EdU) Cell Proliferation Assay

OCYs were seeded onto 3.5 cm coverslips placed in 6-well plates (2.5 × 10^5^ cells/well) and cultured for 24 h, then irradiated with 2 Gy of X-ray, followed by incubation for 24 h. Then, cells were co-incubated with 10 μM of an EdU working solution for 2 h under 5% CO_2_ and 37 °C conditions. Following incubation, EdU fluorescence labeling was achieved using the EdU Cell Proliferation Kit (C0071S; Beyotime Institute of Biotechnology, Shanghai, China). The cell nuclei were stained with a Hoechst solution at room temperature in the dark. The proliferating cells fluoresced green, while the nuclei of all cells fluoresced blue. For each well, images of EdU-positive cells were obtained from 10 random fields at 200× magnification using a Nikon Ni-U compound fluorescence microscope equipped with a Nikon DS-Ri2 camera and the Nikon NIS-Elements Fr software (Nikon Instruments Inc., Melville, NY, USA). Cell counting was facilitated by ImageJ (1.54f; National Institutes of Health, Bethesda, MD, USA), and the percentage of EdU-positive cells was determined using the following formula: EdU-positive rate = (EdU-positive cell count/(EdU-positive cell count + EdU-negative cell count)) × 100%. This was used to visualize the cells’ ability to proliferate.

#### 2.3.3. Morphological Alteration Assays

Specific dendrite-like synapses and cytoskeletal changes were examined using scanning electron microscopy (SEM) and staining procedures. The cells were seeded onto 3.5 cm coverslips placed in 6-well plates (1 × 10^5^ cells/well). After being irradiated with 2 Gy of X-ray, the cells were incubated until they reached 60% confluence and then harvested. Following this, the cells were fixed with an electron microscope fixative (G1102; Servicebio, Wuhan, China) for 24 h at 4 °C. Subsequently, the harvested cells were post-fixed using 0.1 M of phosphate buffer (PH = 7.4), dehydrated through a gradient of ethanol, and coated with a conductive metal layer. They were then examined using a scanning electron microscope (SU8100; Hitachi, Tokyo, Japan) operating at an accelerating voltage of 3.00 kV. The SEM micrographs of the OCYs were obtained with 1500× magnification. Meanwhile, the cytoskeletal changes were observed by using tetramethyl rhodamine (TRITC)-phalloidin (CA1610; Solarbio, Beijing, China) to visualize F-actin, whereas 4′,6-diamino-2-phenylindole (DAPI) (D523; Dojindo, Kumamoto, Japan) was used to highlight the nuclei. Briefly, cells were fixed with 2.5% glutaraldehyde, incubated in the dark at room temperature for 1 h with 200 μL of TRITC-phalloidin working solution (diluted to 1:500), and re-stained for 30 s with 200 μL of DAPI solution (diluted to 1:500) in the dark. Ten random fields were selected in each well, and representative images were captured using a Nikon Ni-U compound microscope equipped with specific TRITC excitation–emission filters (Ex/Em = 540/570 nm) and DAPI excitation–emission filters (Ex/Em = 364/454 nm) at 200× magnification. The dendritic length of the OCYs was measured from randomly selected regions in each well using ImageJ.

### 2.4. DNA Damage and Repair Assays

#### 2.4.1. DNA Damage Detection

The γ-H2AX foci, markers of DNA damage, were examined according to a previously described procedure [40]. The procedures of immunofluorescence staining are described below in detail. γ-H2AX positivity was defined as nuclei containing ≥10 immunoreactive foci.

#### 2.4.2. Cell Cycle and Apoptosis Analysis

The flow cytometry assay was performed to detect the cell cycle and apoptosis of the OCYs. The OCYs were seeded into T25 culture flasks (2.5 × 10^5^ cells/flask) and cultured for 24 h, then irradiated with 2 Gy of X-ray and continued incubation for 72 h, and cell generation cycles and apoptosis were detected. The harvested cells were processed using the Cell Cycle Staining Kit (70-CCS012; Lianke Biotech, Hangzhou, China) and Annexin V-FITC/PI Apoptosis Detection Kit (AP101-30; Lianke Biotech, Hangzhou, China) according to the manufacturer’s instructions. In brief, 1 × 10^6^ cells were collected and washed with PBS. Subsequently, 1 mL of DNA staining solution and 10 μL of permeabilization solution were added, and the mixture was vortexed and shaken for 5–10 s. After incubation for 30 min at room temperature in the dark, the lowest loading rate was selected to detect cell cycles. Likewise, 5 μL of Annexin V-APC and 10 μL of PI were added to detect apoptosis. Detection was carried out using a CytoFlex flow cytometer (CytoFLEX; Beckman Coulter, Inc., Fullerton, CA, USA). The cell cycle profiles were generated using ModFit LT (Version 5.0; verity Software House, Topsham, ME, USA). The cell apoptosis rate was determined using the following formula: cell apoptosis rate (%) = (apoptotic cell number/total cell number) × 100%.

### 2.5. Characteristic Senescence Phenotypes Assays

#### 2.5.1. Senescence-Associated β-Galactosidase (SA-β-Gal) Staining

Briefly, the OCYs were seeded into 6-well plates (1 × 10^5^ cells/well) and incubated for 24 h; then, the cells were irradiated with 2 Gy of X-ray and cultured for an additional 48 h. Following incubation, the cells were washed with PBS and fixed using a fixative solution for 15 min. Subsequently, the cells were incubated with freshly prepared SA-β-gal staining solution (C0602; Beyotime Institute of Biotechnology, Shanghai, China) at 37 °C overnight in a CO_2_-free environment. The number of SA-β-gal-positive cells per 200 cells was counted in randomly selected regions for each well, and the results were expressed as a percentage.

#### 2.5.2. Senescence-Associated Heterochromatin Foci (SAHF) Detection

The SAHF, recognized as a DNase-resistant and DAPI-dense subnuclear structure, is believed to repress genes that promote proliferation, thus aiding in senescence-associated cell cycle arrest [41]. To detect SAHF, cells were immobilized using 4% paraformaldehyde (P1110; Solarbio, Beijing, China) for 15 min. Following 3 washes with PBS, the immobilized cells were permeabilized using 0.1% Triton-X 100 for 10 min, washed again, and stained with DAPI in the dark at room temperature for 5 min. The proportion of SAHF-positive cells was determined by manually examining 200 DAPI-stained cells for each well, which were randomly selected from various regions and observed under a Nikon Ni-U compound microscope equipped with specific DAPI excitation–emission filters (Ex/Em = 364/454 nm) at 400× magnification.

#### 2.5.3. Senescence-Related Gene and Protein Expression Assay

The expression levels of the key senescence marker genes p16 and p21 were detected by means of Western blot and RT-qPCR, respectively. The procedures are described in detail below.

#### 2.5.4. SASP Marker Detection

The supernatant from the OCYs was collected and detected using the Mouse XL Cytokine Array kit (Cat ARY028; R&D Systems, Inc., Minneapolis, MN, USA) and enzyme-linked immunosorbent assay (ELISA) kit (Enzyme-Linked Biotechnology, Shanghai, China) according to the manufacturer’s instructions. First, a total of 111 different cytokines in the supernatant of the cultured OCYs were measured by means of a cytokine antibody microarray. The dot blot membranes were developed utilizing an enhanced chemiluminescence horseradish peroxidase (HRP) substrate. Data were analyzed using the ImageJ software, and the expression (pixel density) was normalized between membranes using positive and negative reference spots on each membrane. Subsequently, the ELISA procedure was utilized to verify the main pro-inflammatory secretory factors. The procedure involved several steps: firstly, 50 µL standard solution with different concentrations and a 50 µL sample were added to blank, standard, or sample wells. Next, 100 µL of HRP-labeled detection antibody was added to each well, except the blank wells. Afterwards, the reaction plate was sealed and incubated at 37 °C for 1 h. After the liquid was discarded, the wells were filled with a washing solution and then repeatedly left to stand for 30 s, emptied, and shaken 5 times. Finally, 50 µL of substrate A and 50 µL of substrate B were added to every well, and the wells were incubated in the dark at 37 °C for 15 min; then, 50 µL of termination solution was added to each well and the absorbance was measured at 450 nm using a microplate reader. The sample concentration was determined using a standard curve based on the measured OD values of the standards.

### 2.6. Regulation of Osteogenesis by CCL3 Secreted from IR-Induced Senescent OCYs

#### 2.6.1. Conditioned Medium (CM) Collection

The OCYs were seeded into T75 flasks (Nunc; Thermo Fisher Scientific, Bagsværd, Denmark) at a density of 1 × 10^6^. After 24 h, the cells were irradiated with 2 Gy of X-ray and left to incubate for 24 h. The OCYs were then further incubated in a fresh volume of serum-free α-MEM for 24 h, and the culture supernatant was gathered, filtered using a 0.22 μm suction filter, and designated as the conditioned medium (CM). Supernatants derived from non-irradiated and irradiated OCYs were labeled as 0 Gy-CM and 2 Gy-CM, respectively, and stored at –80 °C for future co-culture experiments. For experiments involving a CCL3-neutralizing antibody treatment, the irradiated OCY supernatants were treated with 50 ng/mL of the antibody and labeled as 2 Gy-CM + anti-CCL3.

#### 2.6.2. Isolation of Bone-Marrow-Derived Mesenchymal Stem Cells (BMSCs)

Primary BMSCs were isolated from 3-week-old male SD rats. Euthanasia was performed via intraperitoneal injection of an anesthetic, followed by the careful separation of the femur and tibia. Total bone marrow cells were then obtained using a previously established method [42]. The sedimented cells were resuspended in α-MEM and subsequently inoculated into T25 flasks. Following incubation in a humidified incubator at 37 °C with 5% CO_2_ for 48 h, the adherent cells were collected as BMSCs.

#### 2.6.3. Colony-Forming Unit (CFU) Assay

The BMSCs (passage 3) were seeded at a density of 2 × 10^3^ cells into 6 cm Petri dishes. Following a 24 h affixation period to the dish walls, the culture medium was replaced with α-MEM containing 50% CM (0 Gy, 2 Gy, or 2 Gy + anti-CCL3). After 2 weeks of co-culture, cell colonies became visible. These colonies were fixed with methanol for 15 min and stained with crystal violet (C8470; Solarbio, Beijing, China) for an additional 10 min. The colony-forming unit (CFU) was calculated by comparing the number of colonies to the total number of seeded cells in each dish.

#### 2.6.4. Osteogenic Differentiation Potential Assay

The BMSCs (passage 3) were seeded into 48-well plates (3 × 10^4^ cells/well) and subsequently cultured in an osteogenic induction medium comprising α-MEM enriched with 15% FBS, 1% PS, 50 mg/L ascorbic acid, 0.1 µM dexamethasone (ST1254; Beyotime Institute of Biotechnology, Shanghai, China), and 10 mM β-glycerophosphate (50020; Sigma-Aldrich, St. Louis, MO, USA). Additionally, the medium was replaced with α-MEM containing 50% CM (0 Gy, 2 Gy, and 2 Gy + anti-CCL3), which was substituted with a fresh medium at 48 h intervals. After 7 days of osteogenic induction, the cells were immobilized using 2.5% glutaraldehyde for 15 min, rinsed thrice with PBS, and subsequently stained with the BCIP/NBT Alkaline Phosphatase Color Development Kit according to the manufacturer’s instructions. In addition, the mineralized nodule formation was measured in order to evaluate the in vitro mineralization ability. After 21 days of osteogenic induction, alizarin red staining was employed to quantify the mineralized nodules. Specifically, the cells were immobilized in 95% ethanol for 10 min, subsequently exposed to a 0.2% alizarin red S staining solution (0.2%, pH 8.3) (C0140; Beyotime Institute of Biotechnology, Shanghai, China), and incubated at 37 °C for 1 h. After flushing with PBS, the positively stained nodule areas in each group were measured from randomly selected regions for each well using ImageJ, and the results were presented as the area percentage of positive alizarin red staining.

### 2.7. Regulation of Osteoclastogenesis by CCL3 Secreted from IR-Induced Senescent OCYs

#### 2.7.1. Conditioned Medium (CM) Collection

The detailed processes for CM collection of senescent OCYs are described above.

#### 2.7.2. Isolation of Bone-Marrow-Derived Macrophages (BMMs)

BMMs were isolated from 6-week-old male BALB/c mice using two-step Ficoll density gradient sedimentation [43]. Briefly, the lymphocyte separation medium (17544602; Cytiva, Dorset, UK) was placed in the centrifuge tube, followed by the slow addition of an equal volume of whole bone marrow cell suspension. After centrifuging at 20 °C and 2500 rpm for 30 min, the middle layer was transferred to a fresh centrifuge tube, mixed with sterile PBS, and centrifuged at 20 °C and 1000 rpm for 10 min. Subsequently, the collected cells were resuspended in a complete medium and inoculated into T25 flasks. Following a 24 h incubation period at 37 °C and 5% CO_2_, the supernatant was discarded after being centrifuged at 20 °C and 1000 rpm for 10 min. Finally, cells were resuspended in a complete medium supplemented with 25 ng/mL of macrophage colony-stimulating factor (M-CSF) (315-02; Peprotech, Rocky Hill, NJ, USA) and inoculated in 96-well plates for 72 h.

#### 2.7.3. Migration and Invasion Ability of Osteoclast Precursors (OCPs)

The horizontal migration of OCPs was evaluated using the wound healing scratch assay, while their vertical migration was assessed using a trans-well assay in vitro. For the wound healing assay, the BMMs were seeded into 6-well plates (4 × 10^5^ cells/well) and incubated in a complete medium enriched with 25 ng/mL of M-CSF. After confluence, the cells were treated with mitomycin (1 μg/mL) (M5353; Sigma-Aldrich, St. Louis, MO, USA) for 1 h, and then a straight scratch was made in the cell monolayer using a 200 µL pipet tip, immediately followed by washing and incubation in a serum-free medium containing 50% CM (0 Gy, 2 Gy, or 2 Gy + anti-CCL3). After 24 h of culture, the number of cells that had migrated from the wound edge was quantified. For the trans-well assay, the BMMs (2 × 10^5^ cells/well) were cultured in the upper chamber of a trans-well plate in 200 µL of α-MEM with 25 ng/mL of M-CSF for 48 h and then replenished with serum-free α-MEM. Subsequently, 1 mL of α-MEM containing 50% CM (0 Gy, 2 Gy, or 2 Gy + anti-CCL3) was added into the lower chambers, and cells were incubated for an additional 24 h. Following this, the cells in the lower chamber which had migrated from the upper chamber were fixed with 2.5% glutaraldehyde for 5 min and stained with crystal violet for 10 min. Ten random images per well were captured under a light microscope at 100× magnification, and the average number of stained positive cells was calculated.

#### 2.7.4. Tartrate-Resistant Acid Phosphatase (TRAP) Staining

The BMMs were seeded into 96-well plates (2 × 10^4^ cells/well) and incubated in a complete medium enriched with 25 ng/mL of M-CSF for 72 h. Subsequently, the medium was substituted with an osteoclast induction-conditioned medium consisting of 50% CM (0 Gy, 2 Gy, or 2 Gy + anti-CCL3) and was further fortified with 25 ng/mL of M-CSF and 10 ng/mL of receptor activator of nuclear factor kappa-B ligand (RANKL) (315-11; Peprotech, Rocky Hill, NJ, USA). After a 4–5 day induction phase, the osteoclasts were immobilized and stained using the TRAP kit (387A-1KT; Sigma-Aldrich, St. Louis, MO, USA) according to the manufacturer’s guidelines. Cells with positive TRAP staining and more than 5 nuclei were enumerated as osteoclasts.

#### 2.7.5. Bone Resorption Capacity Assay

The BMMs were resuspended in an α-MEM medium enriched with 25 ng/mL of M-CSF and then evenly distributed onto a 96-well osteoclast activity detection plate (2 × 10^4^ cells/well), which had been pre-coated with a thin layer of inorganic three-dimensional crystal material (3989; Corning, NY, USA). Adherent cells were cultured for 3 days, the osteoclast induction-conditioned medium was replaced, and the incubation was continued for 10 days. Subsequently, the induction solution was discarded, and the cells were washed twice with PBS buffer, lysed with 5% sodium hypochlorite (80010428; Sinopharm Chemical Reagent Co., Shanghai, China) for 10 min, rinsed 3 times with distilled water, and then dried at room temperature for 2 h. The bone resorption pits were captured at 40× magnification, and the pit areas were analyzed using ImageJ.

#### 2.7.6. Cytoskeletal Alteration Assay

The cytoskeletal alteration was examined through staining procedures, and TRITC-phalloidin was used to visualize F-actin, whereas DAPI was used to highlight the nuclei. The detailed methods are described above.

#### 2.7.7. Osteoclastogenic Function-Related Gene Expression

The expression levels of the osteoclastogenic function-related genes were detected by means of RT-qPCR, and the methods are described in detail below.

### 2.8. RNA Sequencing and Transcriptome Analysis

High-throughput RNA sequencing (RNA-seq) and transcriptome analysis were performed by Gene Denovo Biotechnology Co., Ltd. (Guangzhou, China), following previously reported protocols [44]. In brief, total RNA was extracted from the primary OCYs treated with 2 Gy X-ray (IR) and 0 Gy X-ray (Ct) using a TRIzol^TM^ reagent (15596018; Invitrogen, CA, USA). The concentration and purity of the extracted RNA were examined using a NanoPhotometer spectrophotometer and agarose gel electrophoresis. The cDNA library was prepared from the reverse-transcribed cleaved RNA fragment standards according to the protocol of Illumina (Illumina, San Diego, CA, USA). The paired-end sequencing of cDNA was performed on the Illumina NovaSeq X Plus. Clean reads from 6 transcriptomes were obtained by removing low-quality data and adaptor sequences, which were then mapped to the genome of Balb/c mice for transcript reconstruction. To identify differentially expressed genes (DEGs), |log_2_ fold change (FC)| > log_2_(1.5) and P value < 0.05 were set as the statistically significant criteria. Furthermore, the DESeq2 analysis encompassed a total of 21,480 genes, consisting of 20,449 reference genes and 1,031 novel genes. The bioinformatic analysis was conducted on the Omicsmart platform (Available online: https://www.omicsmart.com/, assessed on 8 February 2025).

### 2.9. RNA Extraction and RT-qPCR Assay

Total RNA was extracted using the Simply P Total RNA Extraction Kit (BSC52S1; Bioflux, Beijing, China) according to standard protocols. RNA was then reverse-transcribed with the FastKing gDNA Dispelling RT SuperMix (KR118-02; Tiangen Biotech, Beijing, China). The SYBR Green-based RT-qPCR was performed on the ABI QuantStudio 5 (Applied Biosystems, Carlsbad, CA, USA) utilizing a PowerUp SYBR Green Master Mix (Invitrogen; Thermo Fisher Scientific, Inc., Waltham, MA, USA) in a 10 µL reaction volume according to the manufacturer’s protocol. Samples were normalized to the level of GAPDH mRNA and expressed as relative units. The primer sequences of the target genes are listed in Table 1.

### 2.10. Western Blot Analysis

The cells were lysed using a RIPA lysis buffer (P0013B; Beyotime Institute of Biotechnology, China) containing protease inhibitors (ST506; Beyotime Institute of Biotechnology, China) and then placed on ice for 40 min. After lysates were harvested by means of centrifugation at 12,000× *g* for 15 min at 4 °C, the total cellular protein concentrations were quantified using the BCA Protein Assay Kit (P0012; Beyotime Institute of Biotechnology, Shanghai, China) according to the manufacturer’s instructions. The protein (25–40 µg) was subjected to different SDS–polyacrylamide gel electrophoresis (Epizyme Biotech, Shanghai, China) procedures according to its molecular weight and transferred onto 0.2 μm polyvinylidene difluoride (PVDF) membranes (ISEQ00010; Millipore, Billerica, MA, USA). The whole membranes were blocked with 5% skim milk (P0216; Beyotime Institute of Biotechnology, Shanghai, China) or 5% BSA (ST025; Beyotime Institute of Biotechnology, Shanghai, China) for 1 h at room temperature; then, immunoblotting was conducted with primary antibodies (diluted to 1:1000) as follows: E11 (ab11936; Abcam, Cambridge, UK), γ-H2AX (ab81299; Abcam, Cambridge, UK), p16 (ab51243; Abcam, Cambridge, UK), p21 (ab109199; Abcam, Cambridge, UK), CCL3 (AF-450; R&D Systems, Inc., Minneapolis, MN, USA), PI3K (4257; Cell Signaling Technology, Inc., Danvers, MA, USA), P-PI3K (4228; Cell Signaling Technology, Inc., Danvers, MA, USA), Akt (4691; Cell Signaling Technology, Inc., Danvers, MA, USA), P-Akt (4060; Cell Signaling Technology, Inc., Danvers, MA, USA), NF-κB/p65 (8242; Cell Signaling Technology, Inc., Danvers, MA, USA), P-NF-κB/P-p65 (3033; Cell Signaling Technology, Inc., Danvers, MA, USA), and β-actin (13E5, Cell Signaling Technology, Inc., Danvers, MA, USA). After incubation overnight at 4 °C, the membranes were washed and incubated with anti-rabbit or anti-mouse IgG-HRP conjugated antibodies (SA00001-2; SA00001-1; diluted to 1:2000, Proteintech, Wuhan, China) for 1 h at room temperature. Immunoreactive bands were ultimately detected through the use of an ECL Kit (D3308; Beyotime Institute of Biotechnology, Shanghai, China) in conjunction with an Omega Lum™ C Imaging System (Gel Company, San Francisco, CA, USA). The quantitative analysis was carried out using ImageJ, and β-actin was used to normalize the expression of protein levels.

### 2.11. Immunofluorescence Staining

The cells were fixed with 4% cold paraformaldehyde for 15 min and permeabilized with 0.1% Triton-X100 for 10 min at room temperature. Samples were then blocked with 0.2% BSA in PBS and incubated with antibodies (diluted to 1:250) that target γ-H2AX and NF-κB/p65 overnight at 4 °C. Subsequently, the cells were incubated with FITC-labeled goat anti-rabbit IgG (H+L) (diluted to 1:500, A0562; Beyotime Institute of Biotechnology, Shanghai, China) for 2 h in the dark, and their nuclei were stained with DAPI for 5 min. Ten random fields in each well from both the control and treatment groups were captured to observe the γ-H2AX positivity and nuclear translocation of NF-κB/p65 using a Nikon Ni-U compound microscope at 200× magnification.

### 2.12. Statistical Analysis

The statistical analysis and graphic generation were performed using IBM SPSS Statistics (Version 22; IBM Corporation, Armonk, NY, USA) and GraphPad Prism (Version 8.0; GraphPad Software, Inc., San Diego, CA, USA), respectively. Heatmap with cluster analysis was constructed with Origin (Version 2022; originLab, Northampton, MA, USA). All experimental data are presented as mean ± standard deviation (SD). If the data followed a normal distribution and met homogeneity of variance, statistical differences between two groups were assessed using an independent samples *t*-test, or one-way analysis of variance (one-way ANOVA) was used for multiple groups. During the one-way ANOVA analysis, Tukey’s multiple comparison test was further used for comparison between the two groups. When the data did not conform to a normal distribution, a nonparametric test was performed.

## 3. Results

### 3.1. IR Can Induce Morphological Changes and Biological Impairment of OCYs In Vitro

**Figure 1:**
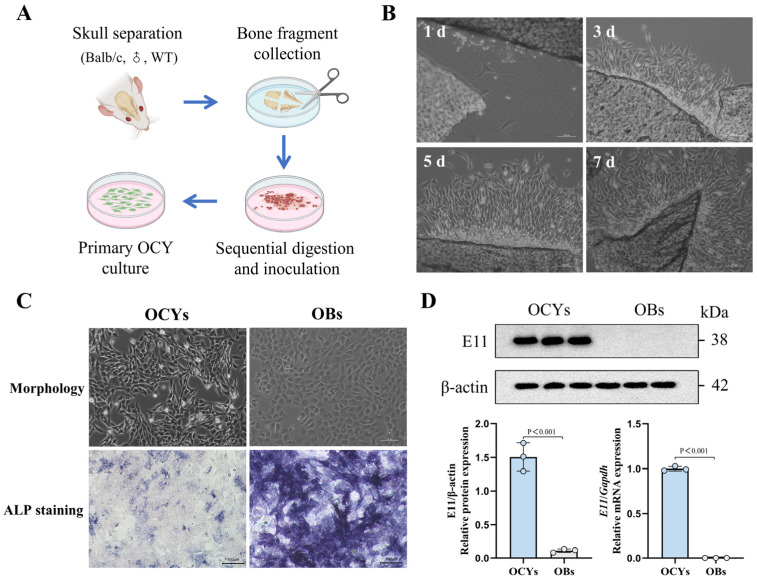
The isolation and identification of primary OCYs. (**A**) A schematic representation of the isolation and in vitro inoculation of primary OCYs. (**B**) The characteristic morphology of primary OCYs under the inverted phase-contrast optical microscope. Scale bar, 100 µm. Magnification, 100×. (**C**) Morphology and alkaline phosphatase (ALP) staining of primary OCYs and OBs, respectively. Scale bar, 100 µm. Magnification, 100×. (**D**) The relative protein and mRNA expression levels of the OCY-specific antigen E11/gp38 obtained using Western blot and RT-qPCR, respectively (n = 3). The data are presented as the mean ± standard deviation (SD).

Primary OCYs, isolated in vitro using a modified sequential digestion method (Figure 1A), were identified based on their characteristic stellate and dendritic morphology, weak or absent ALP expression, and strong expression of the specific antigen E11/gp38 [45,46]. As the culture progressed, the cells gradually migrated out of the bone fragments, continuously proliferated, and exhibited stellate features, forming dendritic structures that are specific to OCYs (Figure 1B and Figure S1). Meanwhile, ALP staining revealed that the number of positively stained cells was notably lower in the primary OCYs compared with the OBs (Figure 1C and Figures S2 and S3). Furthermore, both the mRNA and protein expression levels of the OCY-specific marker protein E11 were significantly elevated compared with those of the OBs (Figure 1D). These findings suggest that the modified sequential digestion method was effective for isolating primary OCYs.

**Figure 2:**
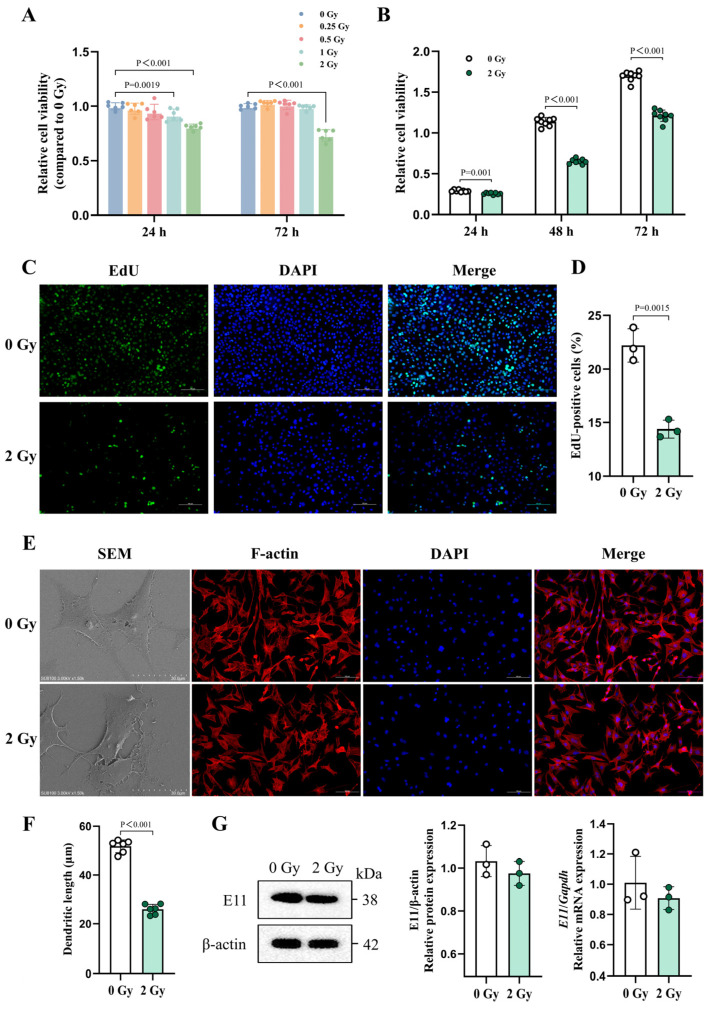
Morphological changes and biological impairment of irradiated OCYs. (**A**,**B**) The cell viability of irradiated OCYs with a dose gradient (**A**) and time gradient (**B**) using the Cell Counting Kit-8 (CCK8) assay (n ≥ 6). (**C**) Representative images showing changes in the proliferative capacity 24 h post-irradiation, with EdU (green) and DAPI (blue) staining. Scale bar, 100 µm. Magnification, 200×. (**D**) Quantification of the proliferative capacity changes (n = 3). (**E**) Representative images of the cytoskeleton and nuclei of irradiated OCYs under scanning electron microscopy (SEM) and immunostaining for F-actin (red) and DAPI (blue). Scale bar, 30 µm (SEM) and 100 µm (immunostaining). Magnification, 1500× (SEM) and 200× (immunostaining). (**F**) Change in the dendritic length of irradiated OCYs (n = 6). (**G**) The relative protein and mRNA expression levels of E11 obtained using Western blot and RT-qPCR, respectively (n = 3). The data are presented as the mean ± SD, and differences that were not statistically significant were not labelled.

To explore the impact of the radiation dose on the cell viability, primary OCYs were irradiated with dose gradients of 0.25, 0.5, 1, and 2 Gy. The results revealed that the X-rays significantly damaged the cell viability, leading to a dose-dependent decrease in the number of OCYs (Figure 2A). Specifically, the cell viability was significantly reduced by 28.5% after 72 h of 2 Gy irradiation compared with the control group (Figure 2B). Simultaneously, the EdU doping experiments revealed a notable decrease in the OCYs’ proliferation capacity by about 34.8% upon exposure to 2 Gy irradiation (Figure 2C,D and Figures S4 and S5). Furthermore, the SEM and F-actin fluorescence staining uncovered distinct morphological alterations in the irradiated OCYs. A typical mature OCY’s morphology presents as multiple outwardly extending dendrites, while the irradiated OCYs exhibited shortened dendrites, indistinct borders, an altered morphology, and a loss of their dendritic branching structure (Figure 2E and Figures S6–S9). Our quantitative analysis further revealed that the dendritic length of the OCYs was reduced by 48.3% following 2 Gy irradiation (Figure 2F). Notably, E11, a hallmark protein for dendritic formation, showed a downregulated expression in its mRNA and protein levels after 2 Gy irradiation (Figure 2G). In summary, 2 Gy of X-rays can cause cellular morphological changes and biological damage in primary OCYs.

### 3.2. IR Can Induce Persistent and Incompletely Repaired DNA Damage in OCYs

**Figure 3:**
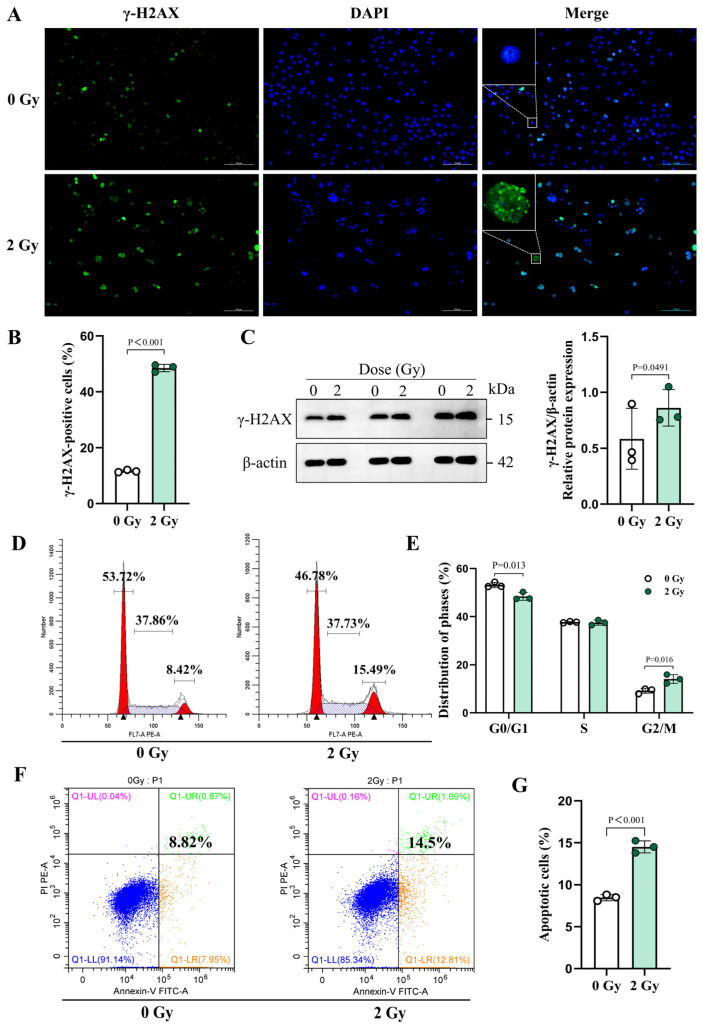
IR-induced DNA damage, cell cycle alteration, and apoptosis in OCYs. (**A**) Representative images of the γ-H2AX immunofluorescence staining (green) and nuclei (blue) in the irradiated OCYs. Scale bar, 100 µm. Magnification, 200×. White rectangles demarcate regions corresponding to higher-magnification insets, with scale bars adjusted proportionally for clarity. (**B**) Quantification of the number of γ-H2AX foci-positive cells, showing the percentage of γ-H2AX foci-positive cells in five separate fields for each group (n = 3). (**C**) The relative protein expression levels of γ-H2AX, determined using Western blot (n = 3). Each dot represents one sample from an independent experiment. (**D**,**E**) Changes in the cell cycle distribution of OCYs at 72 h after irradiation and their quantification (n = 3). (**F**,**G**) Changes in the apoptosis rate of OCYs at 72 h after irradiation and their quantification (n = 3). The data are presented as the mean ± SD, and differences that were not statistically significant are not labelled.

The accumulation of γ-H2AX in cells is a marker of DNA double-strand breaks [47]. Immunofluorescence and Western blot analyses were performed to detect the formation of γ-H2AX foci and its expression levels. The results showed that IR obviously aggravated the DNA damage, which was manifested by increased γ-H2AX foci at 24 h post-2 Gy irradiation (Figure 3A and Figures S10 and S11). Specifically, the percentage of cells in which there were ≥10 γ-H2AX foci in the 2 Gy group surged to 4.18 times that of the control group (Figure 3B). Consistently, Western blot analysis further corroborated these findings, showing a significant elevation in the γ-H2AX protein expression levels after IR (Figure 3C).

When investigating the persistence of the DNA damage caused by 2 Gy irradiation, an altered cell cycle distribution was further detected. Our results demonstrated a significant increase in G2/M phase cells in the 2 Gy group compared with the control group (Figure 3D,E). Simultaneously, the apoptosis assay revealed that the proportion of apoptosis-positive cells doubled in the 2 Gy group relative to the control group, increasing from 8.82% in the control group to 14.5% in the irradiated group (Figure 3F,G). These findings suggest that IR-induced DNA damage is long-lasting and may not be fully repaired. Moreover, OCYs that survive without undergoing apoptosis may be progressing towards senescence, a consequence of incomplete DNA damage repair, leading to permanent cellular growth arrest.

### 3.3. IR Can Induce Characteristic Senescence Phenotypes in OCYs

**Figure 4:**
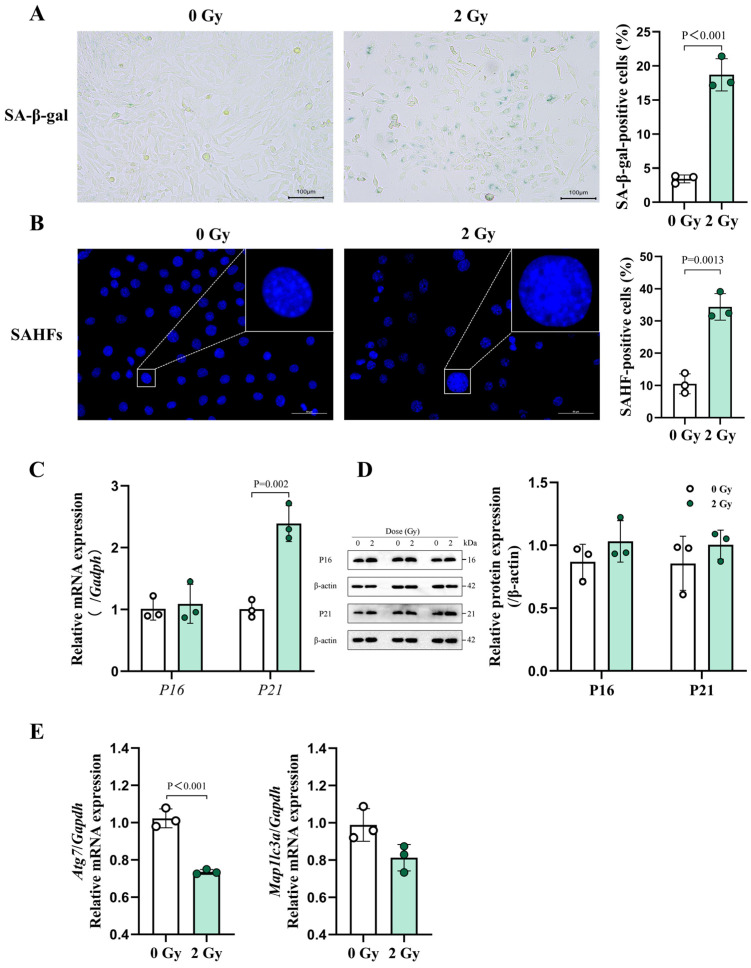
Characterization of IR-induced osteocyte senescence. (**A**) Representative images of SA-β-gal staining in OCYs 3 days post-irradiation and quantification (n = 3). Scale bar, 100 µm. Magnification, 100×. (**B**) Representative images of SAHF, characterized based on the punctate DNA foci in the nuclei (blue) of irradiated OCYs and the percentage of SAHF-positive cells (n = 3). Scale bar, 50 µm. Magnification, 400×. White rectangles demarcate regions corresponding to higher-magnification insets, with scale bars adjusted proportionally for clarity. (**C**,**D**) The relative mRNA and protein expression levels of the senescence-related markers p16 and p21 obtained using RT-qPCR and Western blot analysis, respectively (n = 3). Each dot represents one sample from an experiment. (**E**) The relative mRNA expression levels of the autophagy-related genes *Atg7* and *Map1lc3a* obtained using RT-qPCR (n = 3). The data are presented as the mean ± SD, and differences that were not statistically significant are not labelled.

Senescence-associated β-galactosidase (SA-β-gal) activity is a hallmark characteristic of cellular senescence, as it is a consequence of the increased lysosomal content in senescent cells [15]. Our results revealed a notable increase in SA-β-gal-positive cells in the 2 Gy group, specifically a 5.4-fold surge compared with the control group (Figure 4A and Figures S12 and S13). Apart from this SA-β-gal activity, senescence-associated heterochromatin condensation foci (SAHF), as DNase-resistant and DAPI-dense subnuclear cell structures, are often characterized by senescent cells [41]. In this study, the formation of SAHF in the nuclei was evaluated using DAPI staining. The results indicated that OCYs that had been irradiated with 2 Gy of X-rays exhibited enlarged nuclei, augmented punctate condensation foci, disrupted chromatin structures, and compromised nuclear integrity. Quantitatively, the percentage of SAHF-positive cells increased from 10.5% in the control group to 34.4% in the irradiated group (Figure 4B and Figures S14 and S15).

To further elucidate the molecular mechanisms underlying this process, the expression levels of the key senescence-related proteins p16 and p21 were investigated. Both the RT-qPCR and Western blot analysis revealed an upward trend in the mRNA and protein expression levels of p16 and p21 in the 2 Gy group, with a significant upregulation of the p21 mRNA (Figure 4C,D). Additionally, downward trends in the mRNA expression levels of the autophagy-related genes Atg7 and LC3 were observed in the irradiated group. Notably, the Atg7 mRNA expression was significantly downregulated (Figure 4E). Taken together, these findings indicate that 2 Gy of X-rays can induce DNA damage in primary OCYs in vitro, triggering a cellular senescence response, which may be related to dysfunctional autophagy.

**Figure 5:**
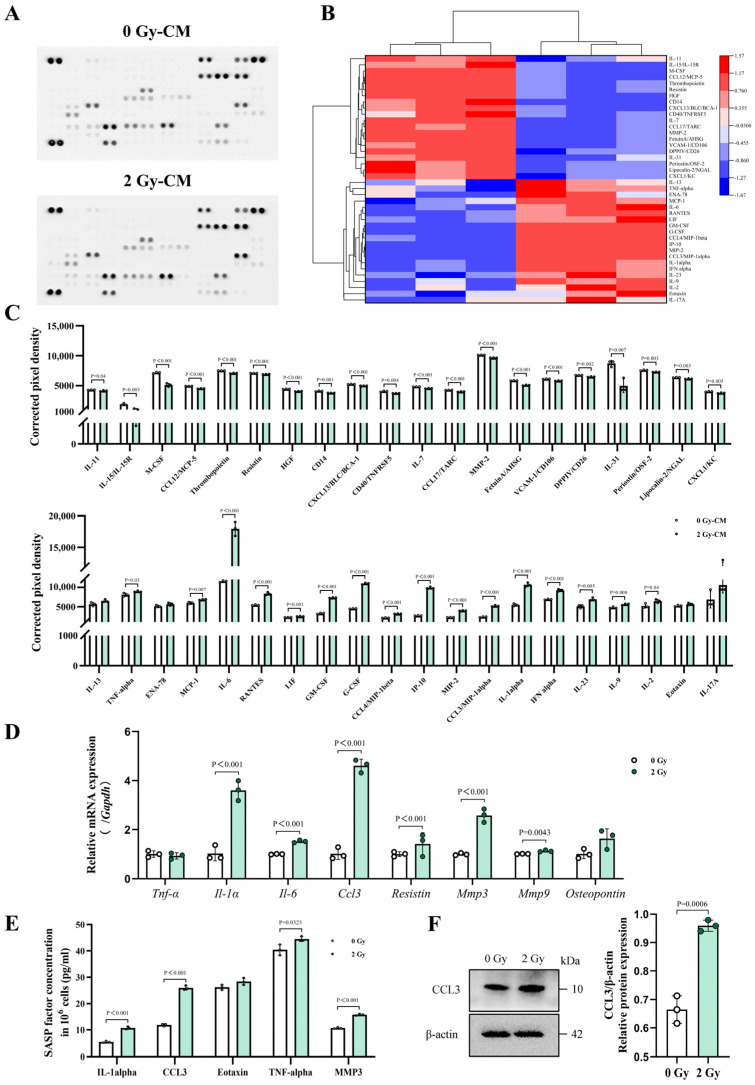
Changes in the secretory phenotype caused by IR-induced senescent OCYs. (**A**,**B**) Alterations in SASP profiles in the supernatant of the irradiated OCYs observed using cytokine antibody microarray analysis and clustering heatmap analysis (n = 3). (**C**) The quantitative expression levels of specific secretome components of the IR-induced senescent OCYs, presented as the corrected pixel density (n = 3). (**D**) The relative mRNA expression levels of key secretome components of the irradiated OCYs, determined using RT-qPCR (n = 3). (**E**) The protein concentrations of major SASP factors in the supernatant, determined using an enzyme-linked immunosorbent assay (n = 3). (**F**) The relative protein expression levels of CCL3, determined using Western blot analysis (n = 3). The data are presented as the mean ± SD, and differences that were not statistically significant are not labelled.

Cellular senescence is also characterized by changes in the SASP. In this study, high-throughput proteomics antibody microarrays were utilized to detect the differential expressions of various SASP factors in the culture supernatants of OCYs. The heatmap and statistical results revealed that the irradiated OCYs exhibited significantly increased levels of several SASP factors, including interleukins (IL-1α; IL-6), chemokines (CCL3/MIP1-α; CXCL10/IP10; CCL5/RANTES), and interferons (IFN-α), whereas the release of certain cytokines, such as IL-31 and CCL12/MCP-5, was reduced compared with the control group (Figure 5A–C). Furthermore, RT-qPCR was employed to validate the expression levels of specific factors in the irradiated OCYs, including the chemokine *Ccl3*, the interleukin-like pro-inflammatory factors *Il-1α* and *Il-6*, *Mmp3* and *Mmp9* (which are related to osteoclast differentiation and maturation), and *Resistin*, which is a crucial regulator of bone metabolism. Our results revealed a significant upregulation of these factors in the irradiated OCYs (Figure 5D). It was explicated that, apart from direct cellular dysfunction, the IR-induced senescence of OCYs also facilitates the overexpression and secretion of SASPs through the paracrine pathway, which in turn may lead to the induction of an inflammatory tissue microenvironment and irreversible bone destruction.

### 3.4. IR Can Induce Over-Excretion of the Chemokine CCL3 in Senescent OCYs

As noted above, the mRNA expression level of *Ccl3* was notably elevated in the irradiated OCYs (Figure 5D). An additional ELISA analysis demonstrated a robust secretion of CCL3, approximately 2.2-fold, in the supernatants of the irradiated OCYs compared with that of the control group (Figure 5E). Moreover, Western blot analysis also confirmed the upregulation of the CCL3 protein expression in the irradiated OCYs (Figure 5F). Collectively, these experiments demonstrated a significant upregulation of CCL3 in IR-induced senescent OCYs.

### 3.5. CCL3 Secreted from IR-Induced Senescent OCYs Is Involved in the Regulation of Osteogenesis

**Figure 6:**
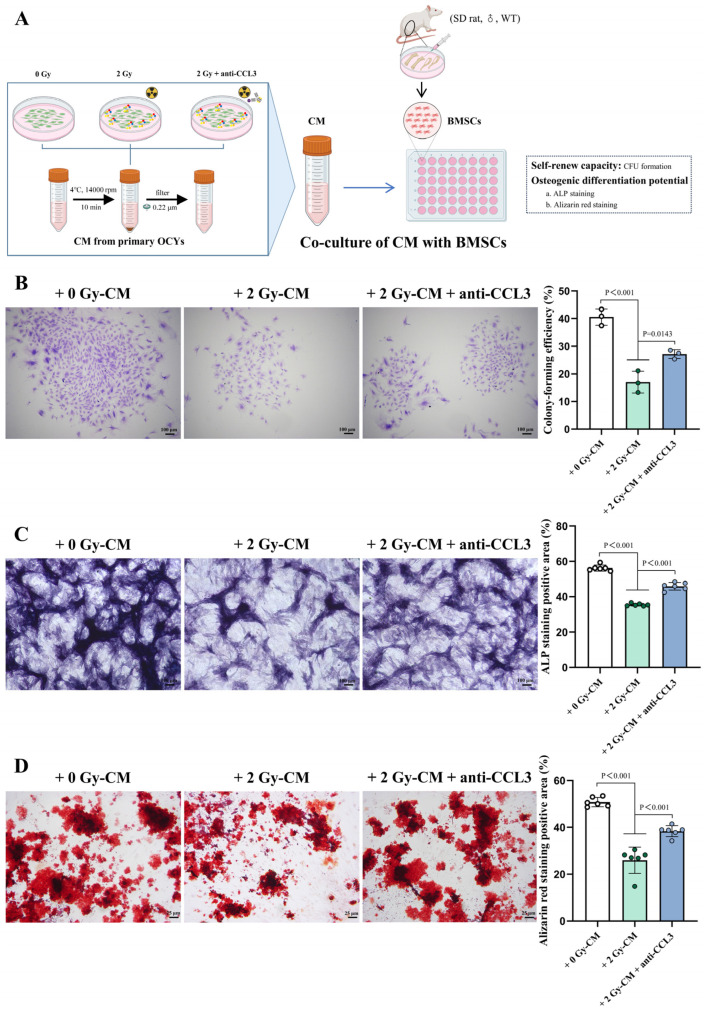
The diminished osteogenic differentiation potential of in vitro BMSCs, regulated by excessive IR-induced CCL3 secretion. (**A**) A schematic representation of the co-culture of bone-marrow-derived mesenchymal stem cells (BMSCs) with different conditioned media (CM). (**B**) Representative images of crystal violet staining to visualize the BMSCs’ colony formation and the quantification of their colony-forming efficiency (n = 3). Scale bar, 100 µm. Magnification, 40×. (**C**) Representative images of the ALP staining and quantification of the ALP-positive expression (n = 6). Scale bar, 100 µm. Magnification, 40×. (**D**) Representative images of the alizarin red (AR) staining and quantification of the in vitro mineralized nodule (n = 6). Scale bar, 25 µm. Magnification, 100×. The data are presented as the mean ± SD.

BMSCs, as progenitor cells of osteoblasts, play a pivotal role in bone formation [48]. To investigate the effects of the robust IR-induced CCL3 secretion by senescent OCYs on the biological function and osteogenic differentiation potential of BMSCs, OCY supernatants from different treatments were co-cultured with BMSCs in vitro (Figure 6A). Subsequently, changes in the colony-forming efficiency and osteogenic differentiation potential of the BMSCs were observed. Our findings indicated that co-culture with 2 Gy-CM impairs the colony-forming efficiency of BMSCs (Figure 6B and Figures S16 and S17), which is accompanied by a significant decrease in their osteogenic differentiation potential, as was evident from the reduced ALP-positive expression and diminished area of positive staining for mineralized nodules compared with the 0 Gy-CM group (Figure 6C,D and Figures S19, S20, S22 and S23). However, upon co-culture with supernatants containing a CCL3-neutralizing antibody (2 Gy-CM + anti-CCL3), this inhibition of the colony-forming efficiency and osteogenic differentiation potential of the BMSCs was partially reversed (Figure 6B–D and Figures S16–S24). These results confirmed that CCL3 secreted by senescent OCYs can impact the biological function of BMSCs via the paracrine pathway of SASPs. Additionally, targeted intervention with a CCL3-neutralizing antibody could considerably alleviate the biological damage caused to BMSCs by senescent OCYs.

### 3.6. CCL3 Secreted from IR-Induced Senescent OCYs Is Involved in the Regulation of Osteoclastogenesis

**Figure 7:**
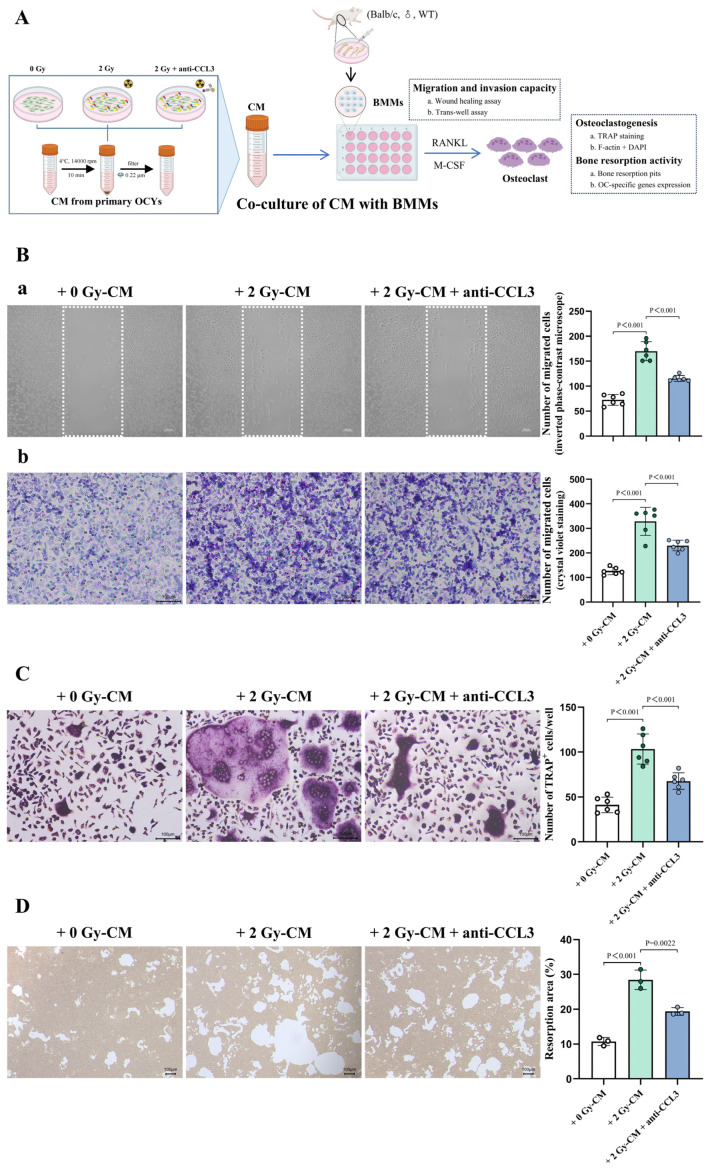
The sthenic osteoclastic differentiation potential of in vitro BMMs regulated by excessive IR-induced CCL3 secretion. (**A**) A schematic representation of the co-culture of bone-marrow-derived macrophages (BMMs) with different conditioned media (CM). (**Ba**) Representative images of the two-dimensional migration ability (determined using a wound-healing scratch assay under an inverted phase-contrast optical microscope; cell migration was assessed by counting the number of osteoclast precursors in the dashed rectangle), and quantification of the migration numbers (n = 6). Scale bar, 100 µm. Magnification, 50×. (**Bb**) Representative images of the three-dimensional migration capability (determined using a trans-well assay with crystal violet staining), and quantification of the migration numbers (n = 6). Scale bar, 100 µm. Magnification, 100×. (**C**) Representative images of OCs that have been stained for TRAP and quantification of TRAP+ cells (≥5 nuclei, n = 6). Scale bar, 100 µm. Magnification, 100×. (**D**) Representative images and quantification of bone resorption pits (n = 3). Scale bar, 100 µm. Magnification, 40×. The data are presented as the mean ± SD.

Likewise, to investigate the effects of the robust IR-induced secretion of CCL3 by the senescent OCYs on the osteoclastogenesis of BMMs, supernatants from OCYs exposed to different treatments were collected in vitro. A targeted intervention was carried out by adding the CCL3-neutralizing antibody to the irradiated OCY supernatants, and the BMMs were co-cultured with these supernatants to assess the changes in osteoclast precursor cell migration, osteoclast (OC) fusion and formation, and bone resorption capacity (Figure 7A). To examine the cell migration and invasion ability of the osteoclast precursor cells, two parallel experiments were performed in vitro: a wound healing scratch assay (horizontal migration) and a trans-well assay (vertical migration). The results of the 24 h scratch assay indicated that the number of migrated osteoclast precursor cells in the co-culture of the 2 Gy-CM group was 2.3 times greater than that of the 0 Gy-CM group under the inverted phase-contrast microscope. Moreover, the number of migrated osteoclast precursor cells in the co-culture of the 2 Gy-CM + anti-CCL3 group decreased by approximately 32.1% compared with that of the 2 Gy-CM group, and this disparity was statistically significant (Figure 7(Ba) and Figures S25–S27). The trans-well assay results further indicated that the number of migrated osteoclast precursor cells in the co-culture of the 2 Gy-CM group was increased by 2.6 times compared with that of the 0 Gy-CM group. Moreover, the addition of CCL3-neutralizing antibody led to a reduction of approximately 30% in the number of migrated osteoclast precursor cells compared with the 2 Gy-CM group (Figure 7(Bb) and Figures S28–S30).

TRAPase serves as a distinctive marker enzyme of OCs, with the extent of positive TRAP staining directly correlating to the OC activity levels [49]. The TRAP staining assay demonstrated a significant enhancement in the osteoclastic differentiation of the co-cultured osteoclast precursor cells within the 2 Gy-CM group. Specifically, there was an approximate 2.5-fold surge in TRAP^+^ cells compared with the 0 Gy-CM group. However, upon the addition of a neutralizing antibody that targets CCL3 at a concentration of 50 ng/mL, a 34.5% decrease in the TRAP^+^ cells was observed compared with the 2 Gy-CM group. These results suggested that the CCL3-neutralizing antibody can partially reverse the overactivity in IR-induced osteoclastogenesis (Figure 7C and Figures S31–S33). Moreover, the assessment of the bone resorption activity by the BMM-derived OCs revealed significant promotion of this activity in the 2 Gy-CM group. The percentage of the bone resorption pit area increased significantly from 10.75 ± 1.15% to 28.45 ± 2.78%, whereas the intervention of the CCL3-neutralizing antibody alleviated the overactivity of the OCs, with the percentage of the bone resorption pit area being reduced to 19.37% (Figure 7D and Figures S34–S36).

**Figure 8:**
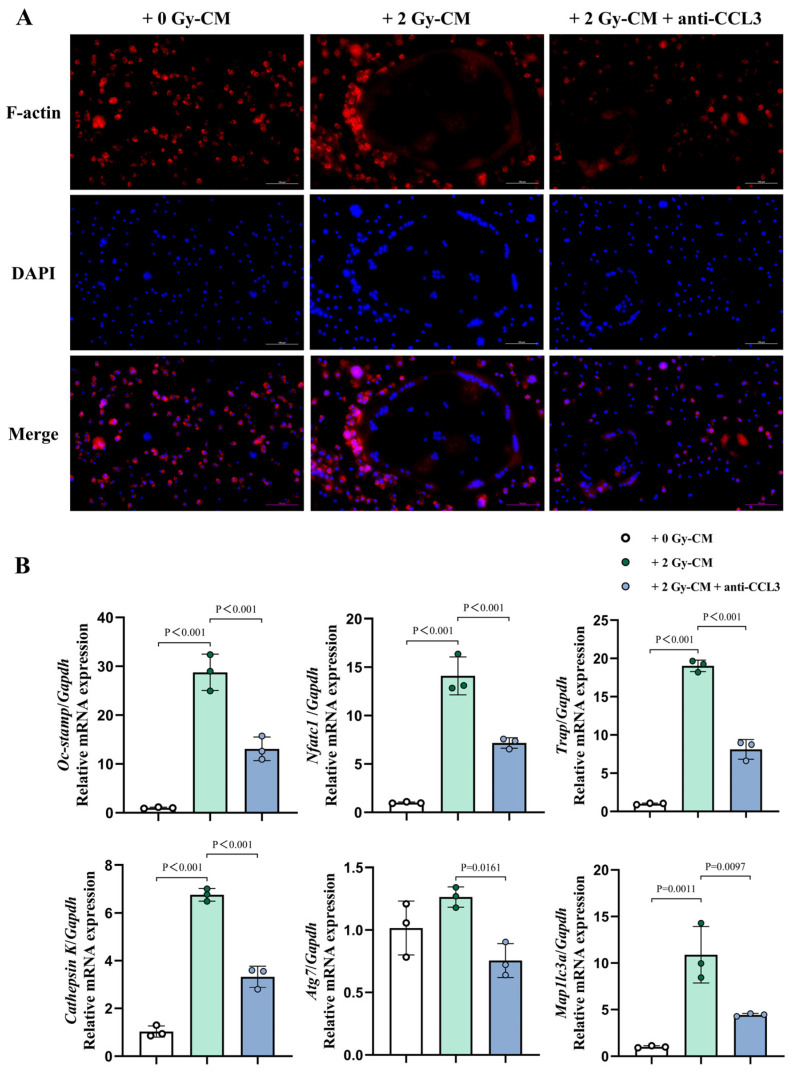
Changes in the cytoskeleton and function of in vitro BMMs, regulated by excessive IR-induced CCL3 secretion. (**A**) Representative images of the typical OC actin rings (red), stained with phalloidin-AlexaFluor488, and nuclei (blue), stained with DAPI. Scale bar, 100 µm. Magnification, 200×. (**B**) The relative mRNA expression levels of key osteoclast functional markers, including *Oc-stamp*, *Nfatc1*, *Trap*, *Cathepsin K*, and the autophagy-related genes *Atg7* and *Map1lc3a*, determined using RT-qPCR (n = 3). The data are presented as the mean ± SD.

Furthermore, upon staining the F-actin cytoskeleton in the OCs, it was observed that, when co-cultured with 2 Gy-CM, the OCs exhibited a characteristic mature morphology, featuring smooth-edged pseudopods. However, upon intervention with CCL3-neutralizing antibody, the actin ring in these co-cultured OCs became disrupted, leading to the emergence of dendritic pseudopods, indicative of a relatively immature cell morphology (Figure 8A and Figures S37–S39). These findings suggested that SASP factors secreted by the irradiated OCYs could promote the differentiation of osteoclast precursor cells into mature OCs. However, the introduction of CCL3-neutralizing antibody partially reversed the stimulatory effect on the osteoclastogenesis. In addition, RT-qPCR was utilized to detect the expression of osteoclastogenic function-related genes, revealing a remarkable upregulation of *Oc-stamp*, *Nfatc1*, *Trap*, and *Cathepsin K*, as well as the autophagy-related genes *Atg7* and *Map1lc3a*, in the co-culture of the 2 Gy-CM group. However, the introduction of the CCL3-neutralizing antibody significantly alleviated the overexpression of these osteoclast functional genes and autophagy-related genes (Figure 8B). Collectively, these findings indicated that CCL3, which is excessively secreted by IR-induced senescent OCYs, could facilitate the migration and fusion of osteoclast precursor cells, and enhance the OC-mediated bone resorption. Based on these findings, CCL3 potentially plays a pivotal role in the homeostatic imbalance of the bone microenvironment that resultins from IR.

### 3.7. CCL3 Can Regulate SASP Secretion and Self-Cascade Amplification via the PI3K/Akt/NF-κB Pathway

**Figure 9:**
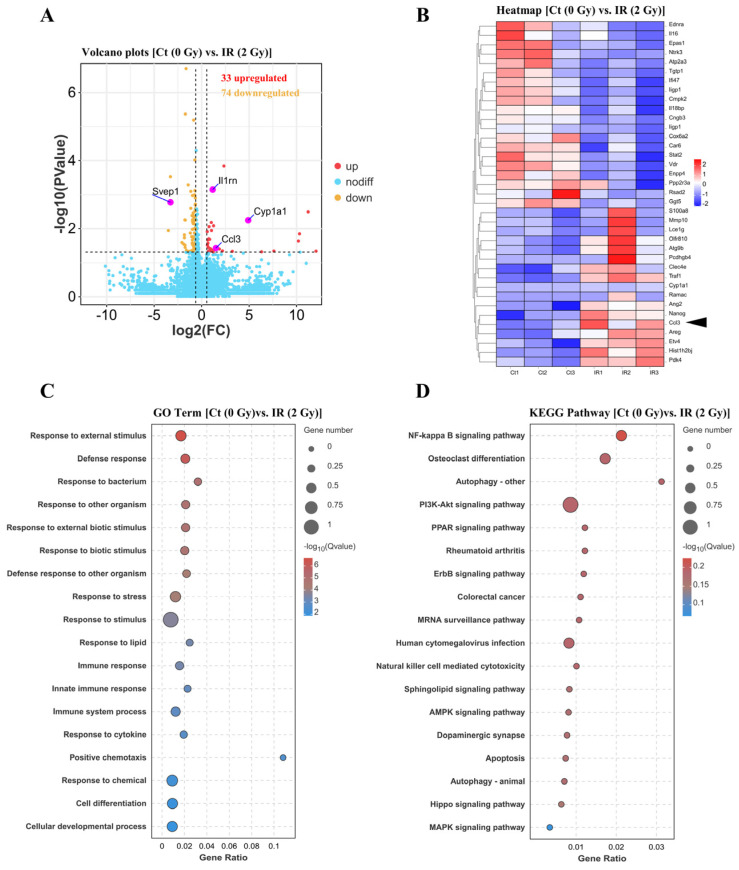
Differential gene expression and signaling pathway enrichment in irradiated OCYs. (**A**) Volcano plots depicting the differential gene expression based on our RNA sequencing analysis. Thirty three significantly upregulated genes are represented in red points, while 74 downregulated genes are marked as orange points. (**B**) A heatmap of the hierarchical clusters of 37 differentially expressed genes in irradiated OCYs, with the black arrow indicating the position of CCL3 within the heatmap. (**C**) A gene ontology (GO) enrichment analysis showing significantly enriched biological functions in the irradiated OCYs. (**D**) A Kyoto Encyclopedia of Genes and Genomes (KEGG) pathway enrichment analysis revealing the significantly enriched signaling pathways. Genes with an adjusted P-value of less than 0.05 were considered differently expressed, and biological functions or pathways with an adjusted Q-value of less than 0.05 were regarded as significantly enriched.

**Figure 10:**
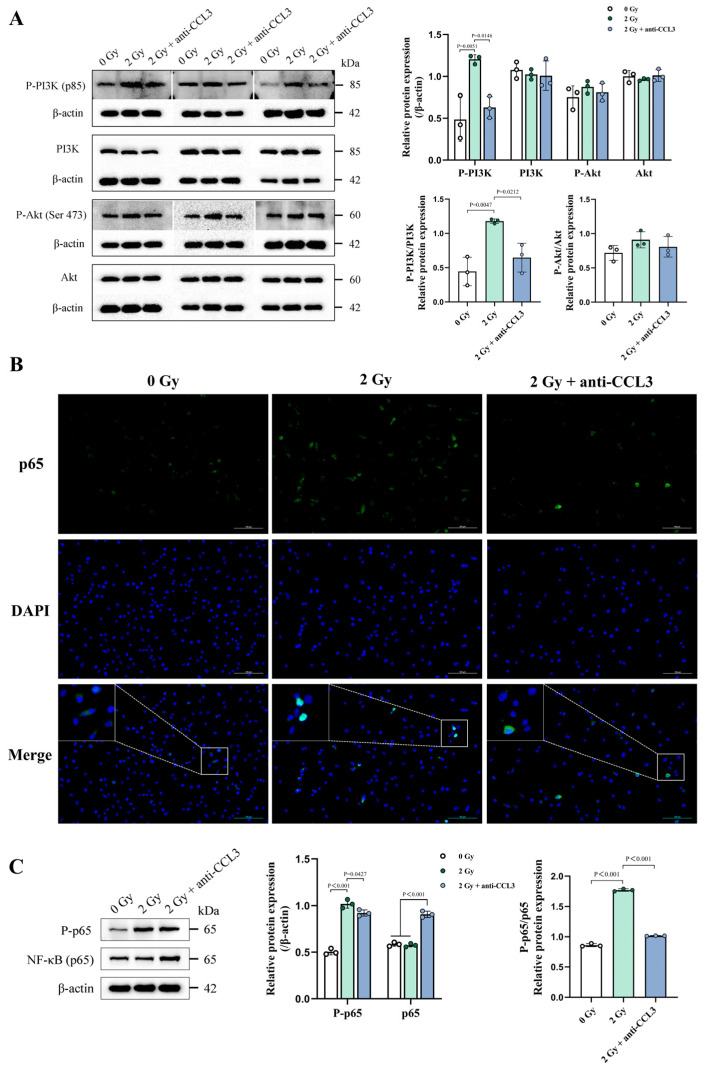
Feedback loop between CCL3 and the PI3K/Akt/NF-κB signaling pathway. (**A**) The relative protein expression levels of p-PI3K, PI3K, p-Akt, and Akt, identified using Western blot (n = 3). (**B**) Representative images showing the nuclear translocation of p65 in the OCYs at 72 h post-irradiation, obtained by means of immunostaining for p65 (green) and DAPI (blue). Scale bar, 100 µm. Magnification, 200×. White rectangles denote regions selected for colocalization analysis between DAPI (nuclei) and p65-positive signals. (**C**) The relative protein expression levels of p-p65 and p65, identified using Western blot analysis (n = 3). The data are presented as the mean ± SD, and differences that were not statistically significant were not labelled.

CCL3 could bind to its receptor, CCR5, triggering a cascade of downstream signaling molecules. This cascade involves the activation of phosphatidylinositol 3-kinase (PI3K) and its related protein kinase B (Akt), which are both critical in immune response regulation and tumor biology [50]. To further investigate the alignment between changes in the CCL3 gene expression levels following IR exposure and the corresponding secretion levels, as well as to investigate alterations in downstream signaling pathways, whole-transcriptome RNA sequencing (RNA-seq) was performed on the irradiated and control OCYs. A differential expression analysis revealed that IR stimulation could significantly upregulate 33 genes, including *Ccl3*, while downregulating 74 genes (Figure 9A). The heatmap, generated from a differential gene analysis across the two groups (Ct and IR), further highlighted the altered expression of *Ccl3* (Figure 9B). In addition, GO and KEGG pathway enrichment analyses based on the above 107 differential genes further revealed that biological processes, particularly the immune response to external stimuli, and signaling pathways such as the PI3K/Akt and NF-κB pathways were predominantly affected (Figure 9C,D). To verify the hypothesis that IR-induced CCL3 engagement might change the inflammatory activation of OCYs, an extracellular flux assay was performed to compare the pathway activation in the control, IR-treated, and CCL3-deficient OCYs. A Western blot analysis revealed that the protein expressions of phospho-PI3K and phospho-Akt in the irradiated OCYs were elevated. Notably, the intervention of the CCL3-neutralizing antibody could significantly reduce the IR-induced elevation of phospho-PI3K and, to a lesser extent, phospho-Akt (Figure 10A). Since the downstream effects of the PI3K/Akt signaling pathway can target NF-κB, thus regulating the secretion of SASP factors, the changes in NF-κB (p65) nuclear translocation were further investigated. The results indicated that p65 was predominantly translocated to the nucleus of the OCYs following exposure to 2 Gy irradiation. However, this nuclear translocation was inhibited in the irradiated OCYs upon treatment with the CCL3-neutralizing antibody (Figure 10B and Figures S40–S42). Meanwhile, the protein expression ratio of phospho-p65/p65 was significantly increased in the irradiated OCYs, and this elevation was significantly reduced following the intervention with the CCL3-neutralizing antibody (Figure 10C). These findings suggest that the activation of the PI3K/Akt/NF-κB pathway and the nuclear translocation of NF-κB might be associated with the upregulation of CCL3 secretion.

## 4. Discussion

Cancer treatment-related bone loss (CTIBL) is of high concern as a notable radiotherapy complication that restricts the prognosis of cancer patients, and further contributes to an elevated risk of fracture and bone metastases. Additionally, the combined impact of SIPS and physiological aging in tumor-prone age groups may bring about substantial imbalances in the reconstruction of both irradiated and distant skeletal sites [2,3,4,8]. Targeting paracrine regulation in senescent OCYs could potentially offer novel research avenues and targets for the prevention and treatment of complications arising from cancer radiotherapy.

As the most abundant long-lived terminally differentiated cells within the bone matrix, OCYs play a crucial role in maintaining skeletal homeostasis, participate in mechano-transduction, and secrete endocrine signals that mediate various organ functions [17,18,19]. The multi-dendritic structure of OCYs plays a pivotal role in their physiological function, facilitating signal communication between OCYs and other cells on the bone surface. Given that OCYs exhibit heightened vulnerability to stress factors, like hypoxia and reactive oxygen species, and face more stringent conditions for nutrient storage, they are prone to direct biological impairment when exposed to microcirculation disorders and oxidative stress triggered by IR [51]. In this study, we adopted a modified sequential digestion method to isolate primary OCYs from mouse calvaria for in vitro experiments. The isolated OCYs showed a dendritic morphology, were negative for ALP expression, and were positive for the E11 antigen. To assess the potential damage of IR on the biological function of OCYs, primary OCYs were exposed to a gradient of X-ray doses in vitro. Our findings revealed a notable decrease in cell viability, particularly under 2 Gy irradiation, along with a significant reduction in their proliferative capacity. Additionally, morphological alterations were observed, including synapse shortening, morphological alterations, and blurred boundaries. The viability and proliferative capacity of cells are crucial for maintaining the biological function of OCYs. These results indicate that IR can result in a decrease in OCYs’ viability and proliferative capacity, thereby impacting their biological function. This provides a crucial cytological foundation for understanding the bone homeostasis imbalance that follows irradiation.

IR can trigger cycles and cascades of oxidation/reduction reactions, which may be beneficial for restoring normal physical function and suppressing tumor growth. However, it can also cause acute DNA damage, leading to cell cycle arrest; DNA repair; and apoptosis, senescence, autophagy, and other cellular responses, which are mediated by interactions between p53 and transcriptional cofactors [6,11]. To clarify whether IR can induce DNA damage in OCYs, further investigations of a characteristic protein marker for the identification DNA damage were conducted in this study [47]. The results revealed a significant amount of clustered DNA double-strand breaks and γ-H2AX accumulation in the irradiated OCYs through immunofluorescence and Western blot analyses. These findings suggest that IR can induce DNA double-strand breaks in OCYs. To analyze the persistence of this damage, further examination of the cell cycle and apoptosis was conducted. Notably, a remarkable increase in the number of G2/M-phase cells was observed after exposure to 2 Gy irradiation, implying that DNA damage induced by IR could trigger cell cycle checkpoints, allowing for additional time for cellular repair mechanisms to address the damage [52,53]. Moreover, the concurrent decrease in cell proliferation and increase in cell apoptosis of the irradiated OCYs suggested that the DNA damage may not have been fully repaired.

IR-induced cellular senescence is a process in which cells that have been exposed to X-ray irradiation undergo changes in their function and structure, resulting in a permanent halt in the cell cycle, which is typically accompanied by reduced cell proliferation [7,14,54]. Previous studies have primarily focused on how IR triggers cell apoptosis or death in OCYs. However, recent research indicates that cells that have evaded apoptosis or death can profoundly impact the outcomes of bone injury and its prognostic regression. This may be attributed to an intermediate state known as minority mitochondrial outer-membrane permeabilization (miMOMP) [55]. Specifically, when cells are exposed to sub-lethal stress conditions, a small subset of mitochondria within them experience MOMP. The occurrence of this phenomenon, rather than cell death, may initiate the release of mitochondrial DNA (mtDNA) into the cytoplasm. The released mtDNA then activates the cGAS-STING pathway, ultimately leading to the induction of SASP. Notably, even a small proportion of senescent cells, ranging from 10% to 15% of the total cell volume, is adequate to cause tissue dysfunction in aged primates [56,57]. However, the identification of cellular senescence remains challenging due to the diverse characteristics of senescent cells [58]. Consequently, there is no single biomarker for their detection, necessitating the combination of multiple indicators to conduct a comprehensive assessment [14,15]. In this study, distinct senescence indicators in post-irradiation OCYs were observed by employing multiple senescence detection indexes, including (a) increased SA-β-gal activity, (b) transcriptional upregulation and concomitant protein accumulation of the the cyclin-dependent kinase inhibitors P16 (CDKN2A) and P21 (CDKN1A), and (c) an enhanced proportion of SAHF-positive cells. Notably, the increase in SAHF-positive cells was correlated with changes in the chromatin structure of the OCYs, indicating an irreversible decline in the OCYs’ proliferative and DNA-replication capabilities [41,59]. These findings imply that exposure to 2 Gy irradiation could cause DNA damage in OCYs, subsequently triggering cellular senescence under stressful conditions. Furthermore, it is notable that the expression levels of two autophagy-related genes, Atg7 and LC3, exhibited a downward trend in the OCYs following irradiation. The preservation of functional autophagy is pivotal for maintaining cellular and tissue health, whereas a decrease in autophagy levels is frequently associated with the initiation of cellular senescence [60]. This finding implies a possible mechanism involving autophagy in the onset of cellular senescence induced by IR.

SASPs represent a distinctive secretome that is generated by senescent cells, encompassing various components such as pro-inflammatory cytokines, growth factors, chemokines, reactive oxygen species, metabolites, and matrix metalloproteinases [14,15,16]. SASPs can initiate inflammation via autocrine and paracrine signaling, exacerbate cellular telomere dysfunction, and accelerate cellular senescence [13,15]. Recent studies indicate that SASPs exhibit intricate biological characteristics and bidirectional effects, which are not solely detrimental [30,61,62]. This understanding underscores the urgency of unraveling the key regulators of SASP secretion and identifying novel targets for clinical prevention and treatment strategies. To thoroughly investigate the changes in SASPs and identify crucial regulatory mechanisms, a high-throughput proteomics antibody microarray was employed to assess the levels of 111 secreted protein factors in the supernatants of the irradiated OCYs. Heatmap and statistical analyses revealed a notable increase in the secretion of SASPs in the culture supernatants of the irradiated OCYs. Furthermore, RT-qPCR and ELISA were used to validate the expression levels of specific SASP factors. These additional analyses provided evidence that IR-induced senescent OCYs exhibit the overexpression and oversecretion of SASPs. Concurrently, based on in vitro cellular experiments and differential gene screening via RNA sequencing, we prioritized the chemokine (C-C motif) ligand 3 (CCL3), a significant SASP factor that is secreted by IR-induced senescent OCYs. The upregulation of the CCL3 gene and protein expression was further verified, as was the enhanced exocytosis level through RT-qPCR, Western blot analysis, and ELISA. The above results unequivocally demonstrated that irradiation can stimulate a remarkable increase in CCL3 secretion in senescent OCYs.

It has been found that aging is associated with OCY dysfunction, while a degradation of the OCY lacunar–myelin network has been observed in both older adults and senescent animal models [24,25]. Furthermore, the accumulation of senescent OCYs may disrupt the osteogenic differentiation potential of BMSCs and promote the generation of OCs, leading to bone homeostasis imbalance [26,27,28]. Senescent OCYs and their SASPs have been shown to be associated with age-related bone loss [23]. The results of this study demonstrated that the co-culture of BMSCs with CM from senescent OCYs could significantly inhibit their osteogenic differentiation ability; meanwhile, the co-culture of osteoclast precursor cells with CM from senescent OCYs could significantly promote their osteoclastic differentiation potential and resorption activity. When a CCL3-neutralizing antibody was added to the CM, it was found to be effective in reversing the negative regulation of the IR-induced senescent OCYs on both osteogenic differentiation and osteoclastic differentiation. This suggests that CCL3 hyper-secretion may play a critical regulatory role in IR-induced bone homeostasis imbalance.

It has been demonstrated in an aged mice model that CCL3 can inhibit the osteogenic differentiation potential of BMSCs by blocking ERK-activated DKK1-upregulation-mediated β-catenin activity [35], which is consistent with our in vitro results. As for osteoclastogenesis, the results of the CCL3-neutralizing antibody intervention experiments provided evidence that enabled us to identify the role of CCL3 in excessive IR-induced osteoclastogenesis. Notably, the expressions of the autophagy-related genes *Atg7* and *Map1lc3a* were also upregulated after the co-culture of the osteoclast precursor cells with CM from senescent OCYs, whereas the expression of autophagy-related genes was significantly reduced following the addition of the CCL3-neutralizing antibody. Previous studies have found that autophagy plays an important role in inflammation-mediated osteoclastogenesis hyperactivity, and a possible mechanism of this is that inflammatory factors can activate autophagy within OCs, thereby promoting the generation of OCs [63]. In addition to affecting osteoclastogenesis, autophagy also influences the osteoclast resorption function during osteolysis. Li et al. [64] found that knocking down *Atg5* and *Atg7* in OCs significantly reduced the amount of bone resorption pits, and the ability of lysosomes to migrate to the ruffled border was diminished, which may be due to the impaired membrane formation and extension of autophagosomes, resulting in decreased cellular degradation and phagocytosis. These results suggest that deleting CCL3 may decrease inflammatory factor secretion, ultimately suppressing cellular autophagy in OCs and counterbalancing excessive osteoclastogenesis and bone resorption activity.

The PI3K/Akt/NF-κB pathway plays a key role in a variety of physiological and pathological processes, including tumorigenesis, immunomodulation, and inflammation [65,66,67]. The binding of CCL3 to its receptor, CCR5, can lead to the activation of PI3K through the activation of G protein-coupled receptors (GPCRs), while the activated PI3K can catalyze the conversion of phosphatidylinositol-4,5-bisphosphate (PIP2) to phosphatidylinositol-3,4,5-trisphosphate (PIP3) on the cell membrane, which in turn activates Akt. Akt can interact with these phospholipids, leading to its translocation to the inner membrane, where it is phosphorylated and activated by phospholipid-dependent kinase 1 (PDK1) and phospholipid-dependent kinase 2 (PDK2); ultimately, the function of these substrates could be regulated by activated Akt [50]. This process plays a key role in several inflammation-related diseases but has rarely been reported in IR-induced inflammatory activation in OCYs. Taking into consideration CCL3’s dual regulatory function in osteogenesis and osteoclastogenesis, this study specifically emphasizes its crucial role. Furthermore, RNA-seq analyses revealed a notable upregulation of CCL3 gene expression in senescent OCYs, concurrent with the activation of the PI3K/Akt/NF-κB signaling pathway. Our Western blot analysis similarly demonstrated a notable increase in the phosphorylation of the PI3K, Akt, and NF-κB (p65) proteins. Importantly, the application of a CCL3-neutralizing antibody reduced the overexpression of hyperphosphorylated proteins, suggesting that CCL3 may directly influence the activation of this signaling cascade. Under physiological conditions, the NF-κB complex is in an inactivated state, because it binds to the inhibitor IκB. Phosphorylated IKK can degrade IκB so that the NF-κB complex is released and ectopically translocated to the nucleus, which regulates the expression of genes and the secretion level of SASPs through transcription factors. The nuclear translocation of NF-κB was further examined in this study, and it was found that 2 Gy irradiation could promote the translocation of NF-κB to the nucleus, thereby upregulating the expression of pro-inflammatory factors. In contrast, the nuclear translocation of p65 in the irradiated OCYs was inhibited by the CCL3-neutralizing antibody. Based on the above findings, we speculate that the activation of CCL3 may trigger a cascade amplification effect, creating a positive feedback loop of CCL3 autocrine production between the CCL3 and the PI3K/Akt/NF-κB signaling pathways, which further promotes IR-induced bone homeostasis imbalance. Furthermore, in vivo irradiation experiments are necessary to validate the findings of this study and ensure that they accurately reflect physiological conditions and simulate clinical radiotherapy scenarios. The results of the animal experiments were expected to confirm the clinical relevance of this study, as hypothesized.

Although our study has identified CCL3 as a pivotal component of the SASP secreted by IR-induced senescent OCYs, several other crucial factors that contribute to IR-induced bone homeostasis disruptions deserve further exploration. Additionally, our research merely establishes experimental evidence and the foundation for identifying novel targets to treat IR-induced imbalances in bone homeostasis. Considerable work remains to be done before achieving clinical applications. Moreover, to gain a deeper understanding of the precise mechanisms facilitated by CCL3, it is essential to specifically incorporate anti-CCL3-treated samples into RNA-seq analysis.

## 5. Conclusions

This study has provided experimental evidence and a theoretical basis for understanding the mechanistic connection between OCY senescence and bone microenvironmental homeostasis in the context of IR exposure. This process is mediated by SASP factors, with CCL3 having been identified as an important regulator. CCL3 can bind to its receptor, CCR5, further activating the PI3K/Akt/NF-κB signaling pathway. This activation may exacerbate the accumulation of senescent OCYs and enhance the IR-induced excessive secretion of SASP factors, ultimately leading to a substantial imbalance in bone homeostasis.

## Figures and Tables

**Table 1:** Primer sequences for quantitative reverse-transcription polymerase.

Target Gene	Forward Primer	Reverse Primer
*E11*	GTTTTGGGGAGCGTTTGGTTC	CATTAAGCCCTCCAGTAGCAC
*P16*	GAACTCTTTCGGTCGTACCC	AGTTCGAATCTGCACCGTAGT
*P21*	CCTGGTGATGTCCGACCTGTTC	CGAAGTCAAAGTTCCACCGTTCTC
*Atg7*	GCTCCTCACTTTTTGCCAACA	GCCACCACATCATTGCAGAA
*Map1lc3a*	GCTGTAAGGAGGTGCAGCAGAT	CCTTGTAGCGCTCGATGATCA
*Tnf-α*	TCAGAATGAGGCTGGATAAG	GGAGGCAACAAGGTAGAG
*Il-1α*	AAGAGACCATCCAACCCAGATC	CCTGACGAGCTTCATCAGTTTG
*Il-6*	ATGAACAACGATGATGCACTTG	GGTACTCCAGAAGACCAGAGG
*Ccl3*	CTCCCAGCCAGGTGTCATTTT	CTTGGACCCAGGTCTCTTTGG
*Resistin*	ACAAGACTTCAACTCCCTGTTTC	TTTCTTCACGAATGTCCCACG
*Mmp3*	TTGACGATGATGAACGATGGA	GAGCAGCAACCAGGAATAGGTT
*Mmp9*	TGAGTCCGGCAGACAATCCT	CCCTGGATCTCAGCAATAGCA
*Osteopontin (OPN)*	ATCTCACCATTCGGATGAGTCT	TGTAGGGACGATTGGAGTGAAA
*Oc-stamp*	AGCCACGGAACACCTCTT	TGGGTCAGTAGTTCGTTACAG
*Nfatc1*	GGAGAGTCCGAGAATCGAGAT	TTGCAGCTAGGAAGTACGTCT
*Trap*	TGTCATCTGTGAAAAGGTGGTC	ACTGGAGCAGCGGTGTTATG
*Cathepsin K*	CTCGGCGTTTAATTTGGGAGA	TCGAGAGGGAGGTATTCTGAGT
*Gapdh*	AGGTCGGTGTGAACGGATTTG	GGGGTCGTTGATGGCAACA

## Data Availability

The datasets generated and analyzed in the present study are all included in this published article.

## Supplementary Materials

The following supporting information can be downloaded at https://www.mdpi.com/article/10.3390/cells14040249/s1. Figure S1. Original images of primary OCYs characteristic morphology under inverted phase-contrast optical microscope; Figure S2. Original images of primary OCYs and OBs morphology under inverted phase-contrast optical microscope, respectively; Figure S3. Original images of alkaline phosphatase (ALP) staining in primary OCYs and OBs under LM, respectively; Figure S4. Original image of EdU (green) and DAPI (blue) staining in primary OCYs; Figure S5. Original image of EdU (green) and DAPI (blue) staining in irradiated OCYs; Figure S6. Original image of primary OCYs morphology under SEM; Figure S7. Original image of irradiated OCYs morphology under SEM; Figure S8. Immunostaining of primary OCYs using phalloidin-AlexaFluor488 and DAPI to visualize the cytoskeleton (red) and the nuclei (blue), respectively; Figure S9. Immunostaining of irradiated OCYs using phalloidin-AlexaFluor488 and DAPI to visualize the cytoskeleton (red) and the nuclei (blue), respectively; Figure S10. Immunofluorescence staining for γ- H2AX in primary OCYs: γ- H2AX (green) and DAPI (blue); Figure S11. Immunofluorescence staining for γ- H2AX in irradiated OCYs: γ- H2AX (green) and DAPI (blue); Figure S12. Original image of SA-β-gal staining in primary OCYs under LM; Figure S13. Original image of SA-β-gal staining in irradiated OCYs under LM; Figure S14. Original image of SAHFs formation in primary OCYs nuclei under FM; Figure S15. Original image of SAHFs formation in irradiated OCYs nuclei under FM; Figure S16. Original image of crystal violet staining in BMSCs co-cultured with 0 Gy-CM under LM; Figure S17. Original image of crystal violet staining in BMSCs co-cultured with 2 Gy-CM under LM; Figure S18. Original image of crystal violet staining in BMSCs co-cultured with 2 Gy-CM + anti-CCL3 under LM; Figure S19. Original image of ALP staining in BMSCs co-cultured with 0 Gy-CM under LM; Figure S20. Original image of ALP staining in BMSCs co-cultured with 2 Gy-CM under LM; Figure S21. Original image of ALP staining in BMSCs co-cultured with2 Gy-CM + anti-CCL3 under LM; Figure S22. Original image of alizarin red staining in BMSCs co-cultured with 0 Gy-CM under LM; Figure S23. Original image of alizarin red staining in BMSCs co-cultured with 2 Gy-CM under LM; Figure S24. Original image of alizarin red staining in BMSCs co-cultured with 2 Gy-CM + anti-CCL3 under LM; Figure S25. Original image of wound-healing scratch assay in osteoclast precursor cells co-cultured with 0 Gy-CM under inverted phase-contrast optical microscope; Figure S26. Original image of wound-healing scratch assay in osteoclast precursor cells co-cultured with 2 Gy-CM under inverted phase-contrast optical microscope; Figure S27. Original image of wound-healing scratch assay in osteoclast precursor cells co-cultured with 2 Gy-CM + anti-CCL3 under inverted phase-contrast optical microscope; Figure S28. Original image of trans-well assay in osteoclast precursor cells co-cultured with 0 Gy-CM under LM; Figure S29. Original image of trans-well assay in osteoclast precursor cells co-cultured with 2 Gy-CM under LM; Figure S30. Original image of trans-well assay in osteoclast precursor cells co-cultured with 2 Gy-CM + anti-CCL3 under LM; Figure S31. Original image of TRAP staining in OCs co-cultured with 0 Gy-CM under LM; Figure S32. Original image of TRAP staining in OCs co-cultured with 2 Gy-CM under LM; Figure S33. Original image of TRAP staining in OCs co-cultured with 2 Gy-CM + anti-CCL3 under LM; Figure S34. Original image of bone resorption pits in OCs co-cultured with 0 Gy-CM under LM; Figure S35. Original image of bone resorption pits in OCs co-cultured with 2 Gy-CM under LM; Figure S36. Original image of bone resorption pits in OCs co-cultured with 2 Gy-CM + anti-CCL3 under LM; Figure S37. Immunostaining of the OCs co-cultured with 0 Gy-CM using phalloidin-AlexaFluor488 and DAPI to visualize the typical OC actin rings (red) and the nuclei (blue), respectively; Figure S38. Immunostaining of the OCs co-cultured with 2 Gy-CM using phalloidin-AlexaFluor488 and DAPI to visualize the typical OC actin rings (red) and the nuclei (blue), respectively; Figure S39. Immunostaining of the OCs co-cultured with 2 Gy-CM + anti-CCL3 using phalloidin-AlexaFluor488 and DAPI to visualize the typical OC actin rings (red) and the nuclei (blue), respectively; Figure S40. Immunofluorescence staining for nuclear translocation of p65 in primary OCYs: p65 (green) and DAPI (blue); Figure S41. Immunofluorescence staining for nuclear translocation of p65 in irradiated OCYs: p65 (green) and DAPI (blue); Figure S42. Immunofluorescence staining for nuclear translocation of p65 in irradiated OCYs treated with anti-CCL3: p65 (green) and DAPI (blue).
