# Supplementary material for: Oversecretion of CCL3 by Irradiation-Induced Senescent Osteocytes Mediates Bone Homeostasis Imbalance
Source: Cells. 2025 Feb 10;14(4):249. doi: 10.3390/cells14040249 (PMC11853822; doi:10.3390/cells14040249)
Supplement: Supplementary file 1 [file cells-14-00249-s001.zip › cells-3430810-supplementary.pdf]

Oversecretion of CCL3 by Irradiation-Induced Senescent Osteocytes Mediates Bone Homeostasis Imbalance

Fanyu Zhao, Haiqing Han, Jing Wang, Jianping Wang, Jianglong Zhai and Guoying Zhu*

Institute of Radiation Medicine, Fudan University, 2094 Xietu Road, Shanghai 200032, China;

* Correspondence: zhugy@shmu.edu.cn; Tel.: 0086-21-64049847

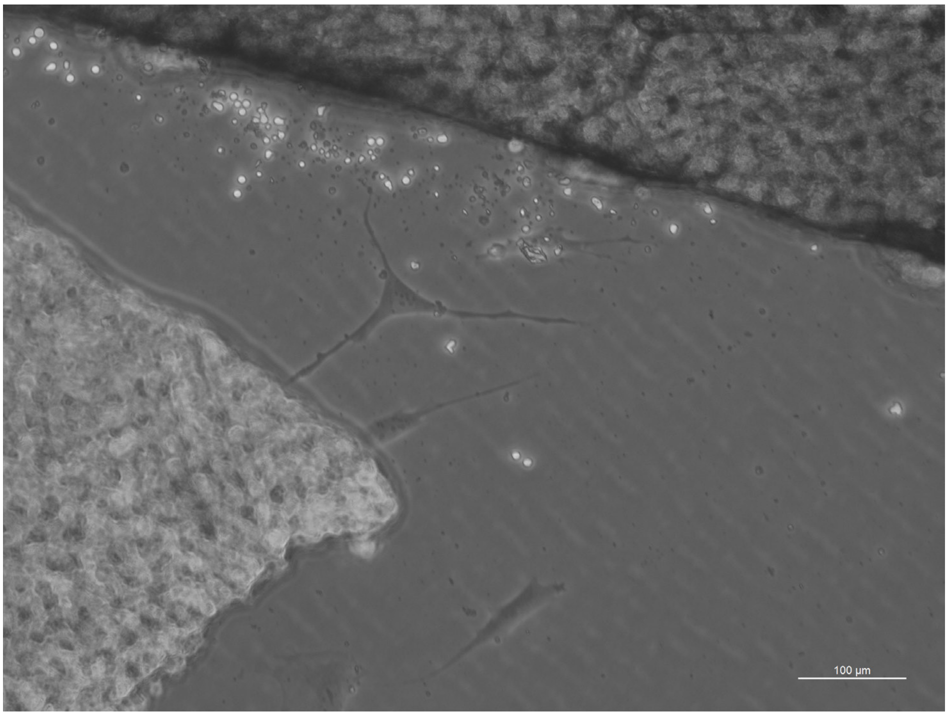

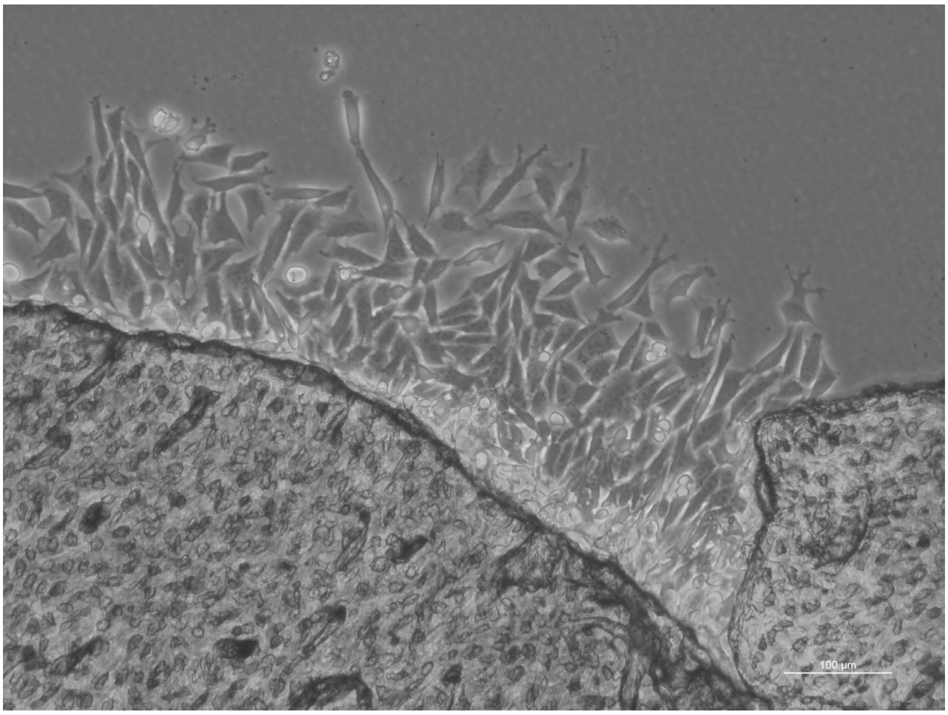

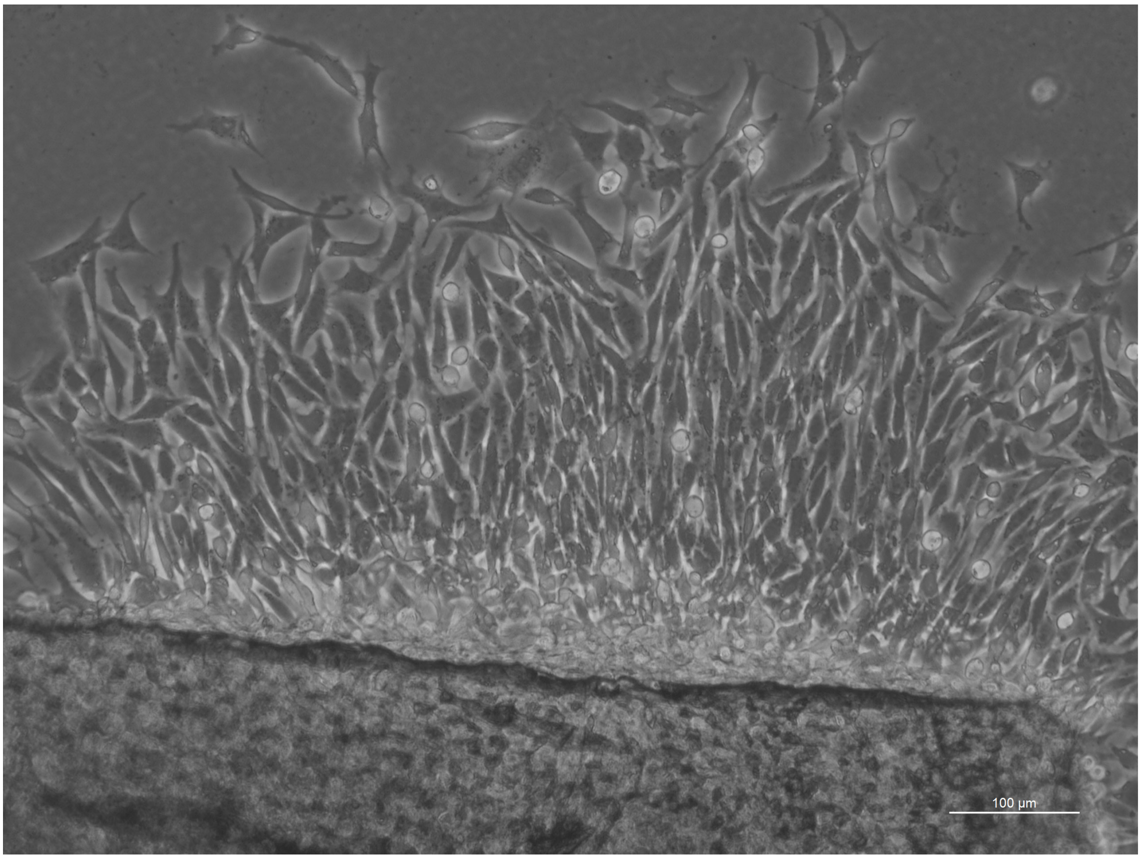

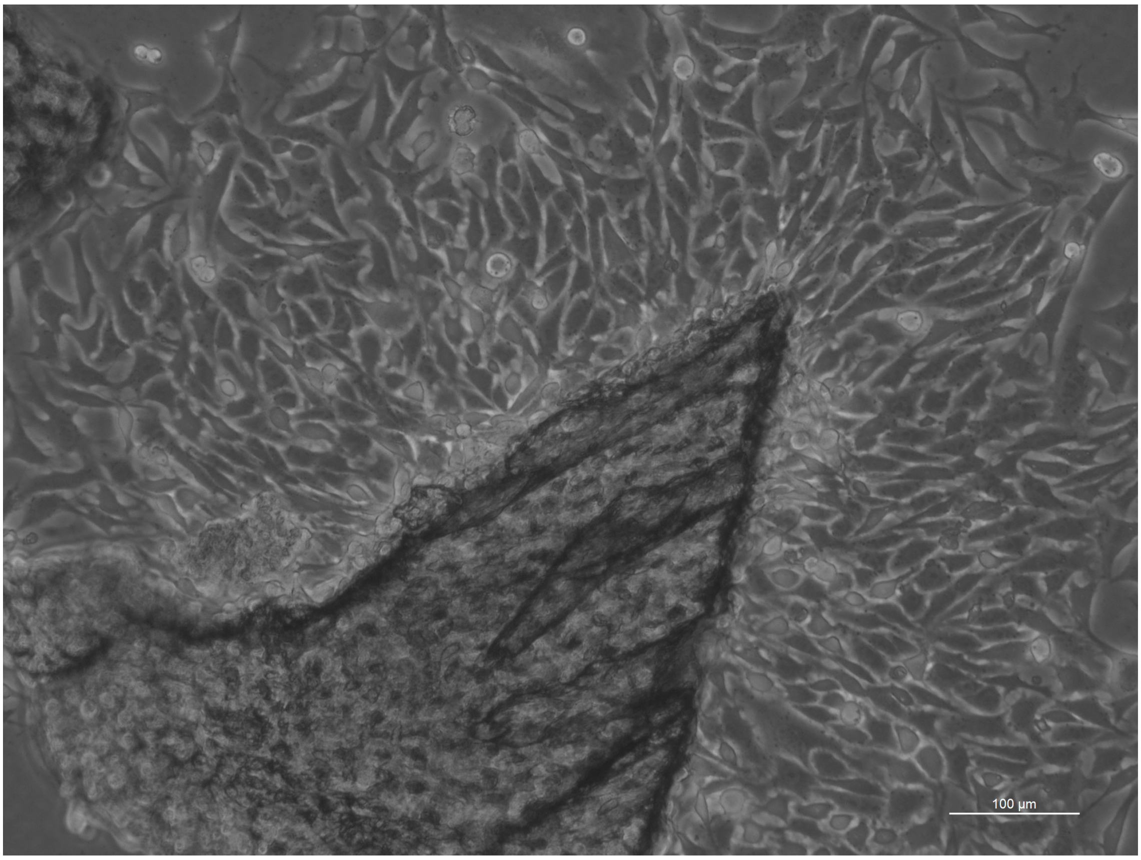

Figure S1. Original images of primary OCYs characteristic morphology under inverted phase-contrast optical microscope; Magnification = 100×.

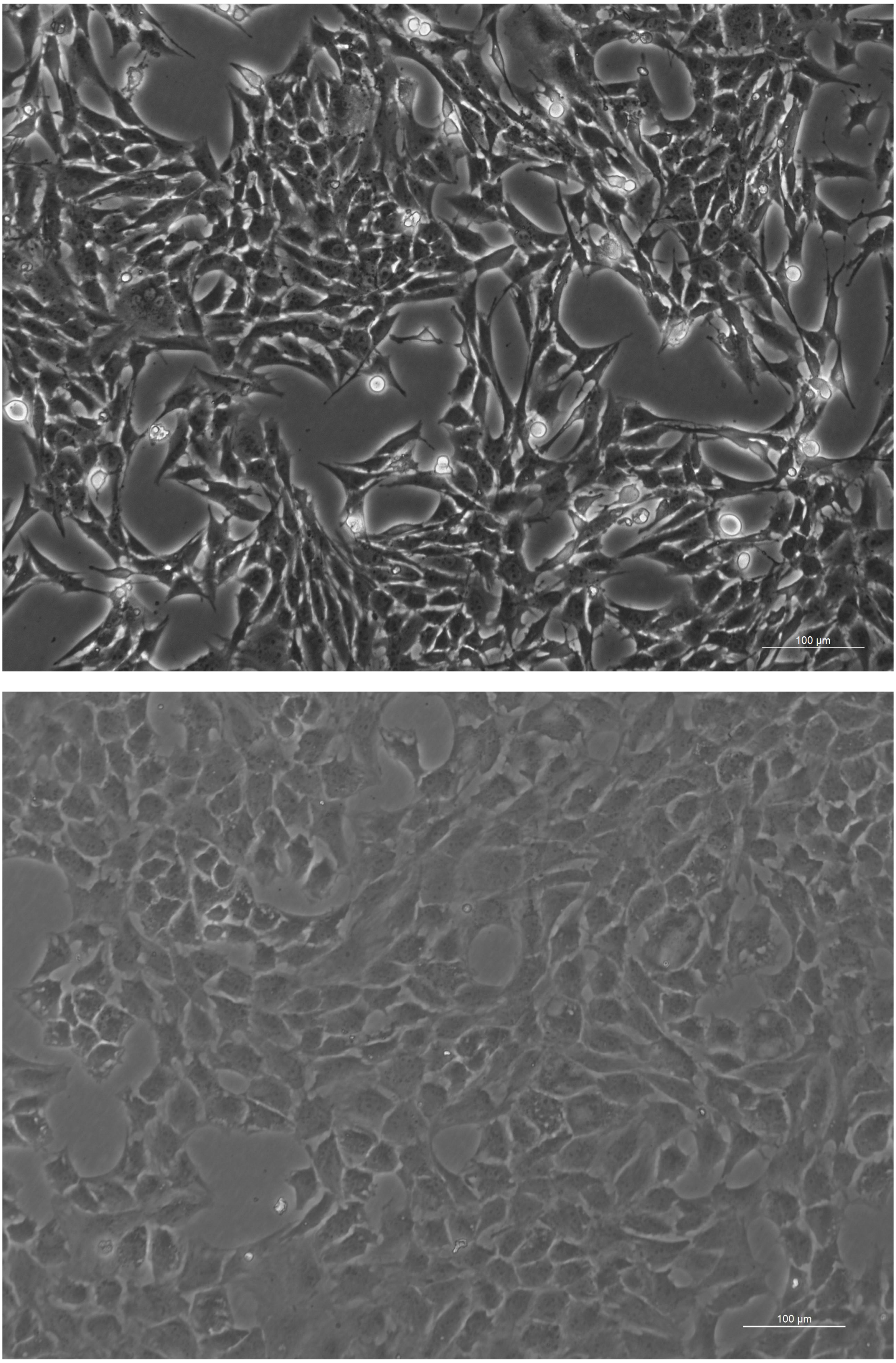

Figure S2. Original images of primary OCYs and OBs morphology under inverted phase-contrast optical microscope, respectively; Magnification = 100×.

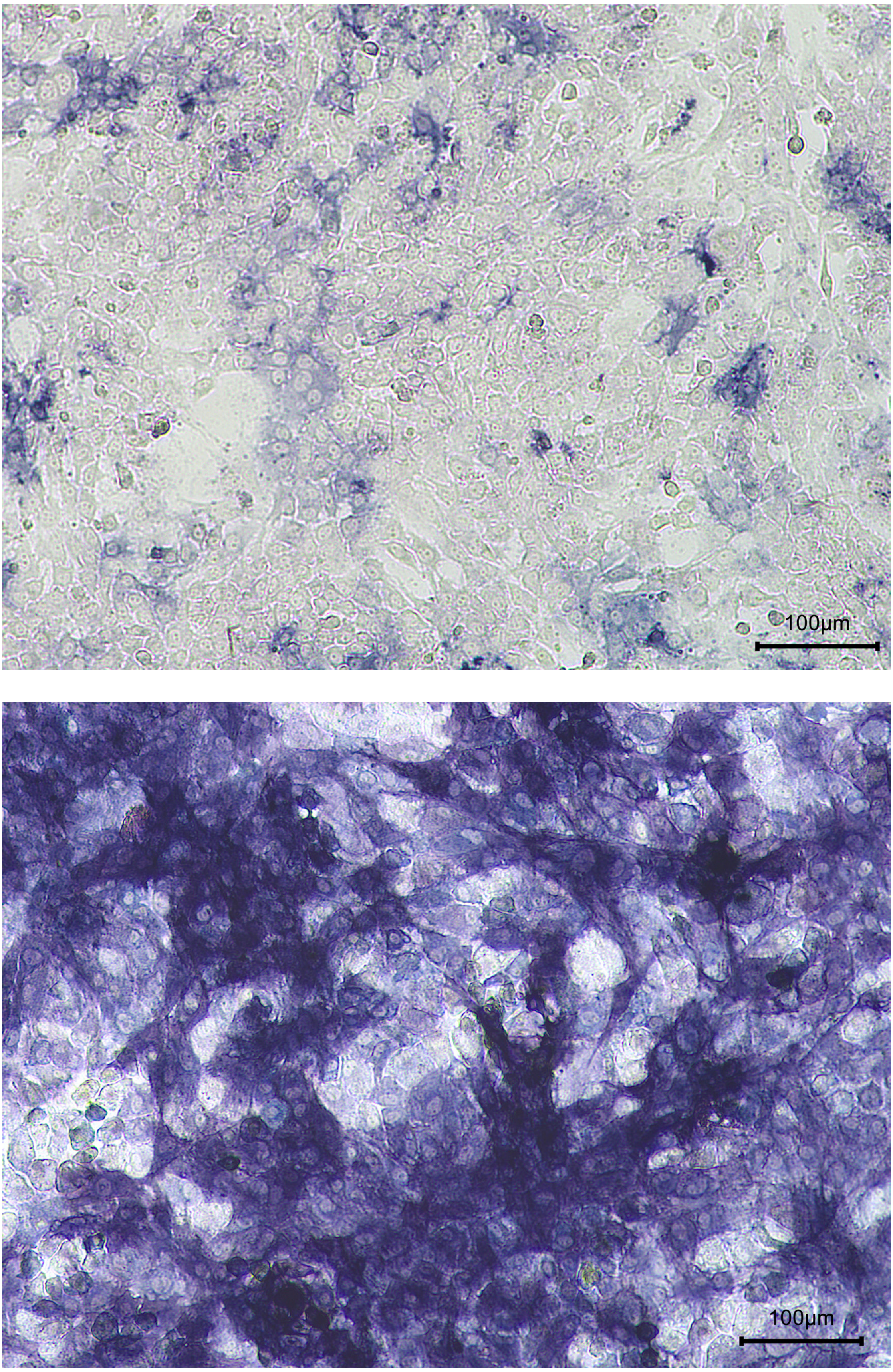

Figure S3. Original images of alkaline phosphatase (ALP) staining in primary OCYs and OBs under LM, respectively; Magnification = 100×.

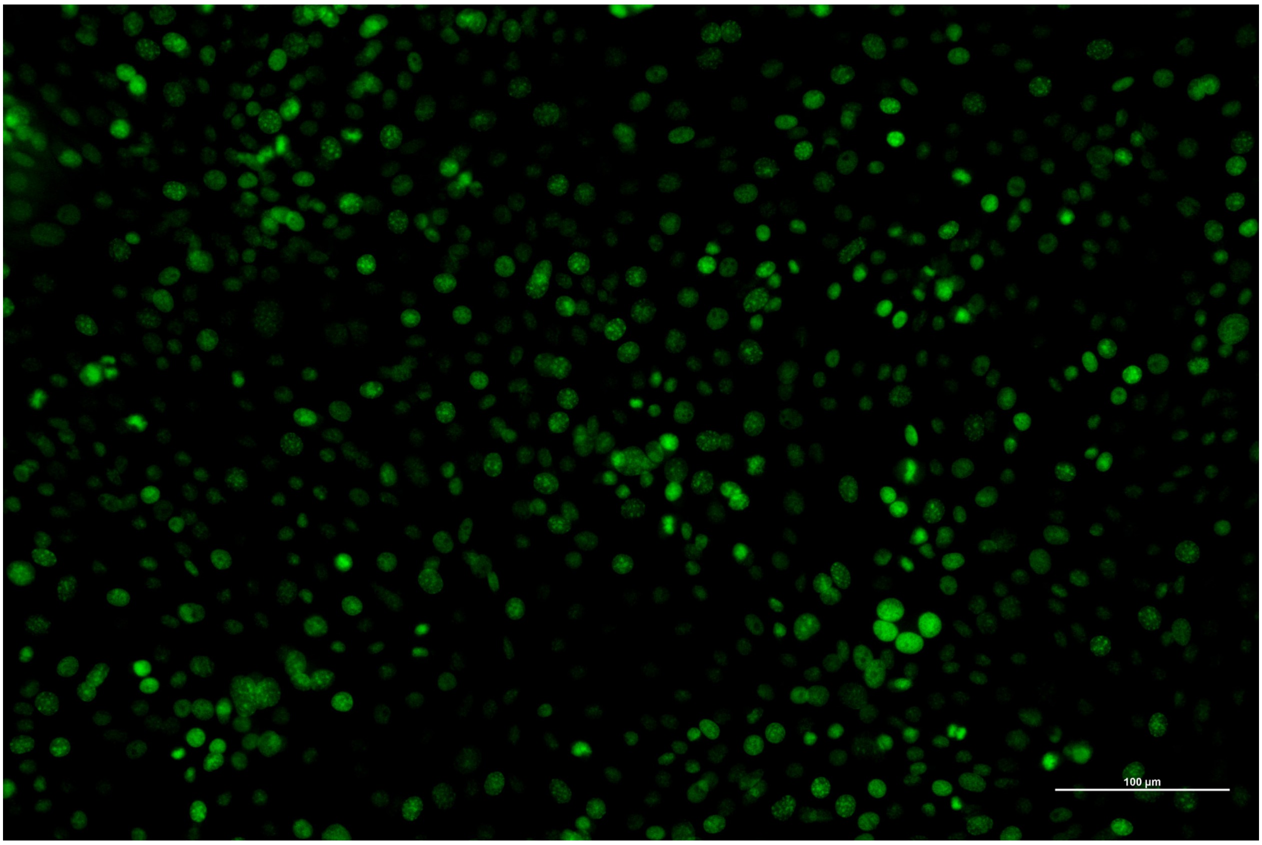

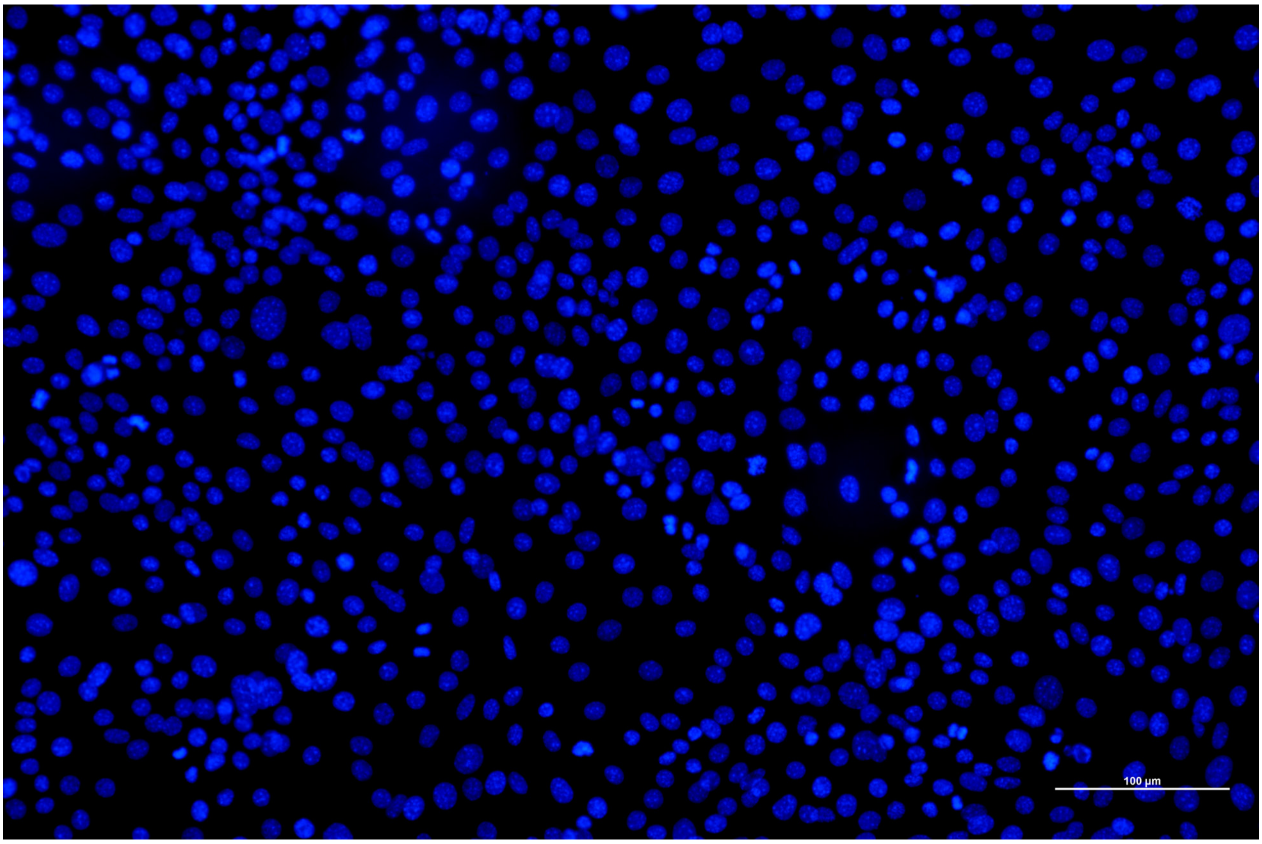

Figure S4. Original image of EdU (green) and DAPI (blue) staining in primary OCYs; Magnification = 200×.

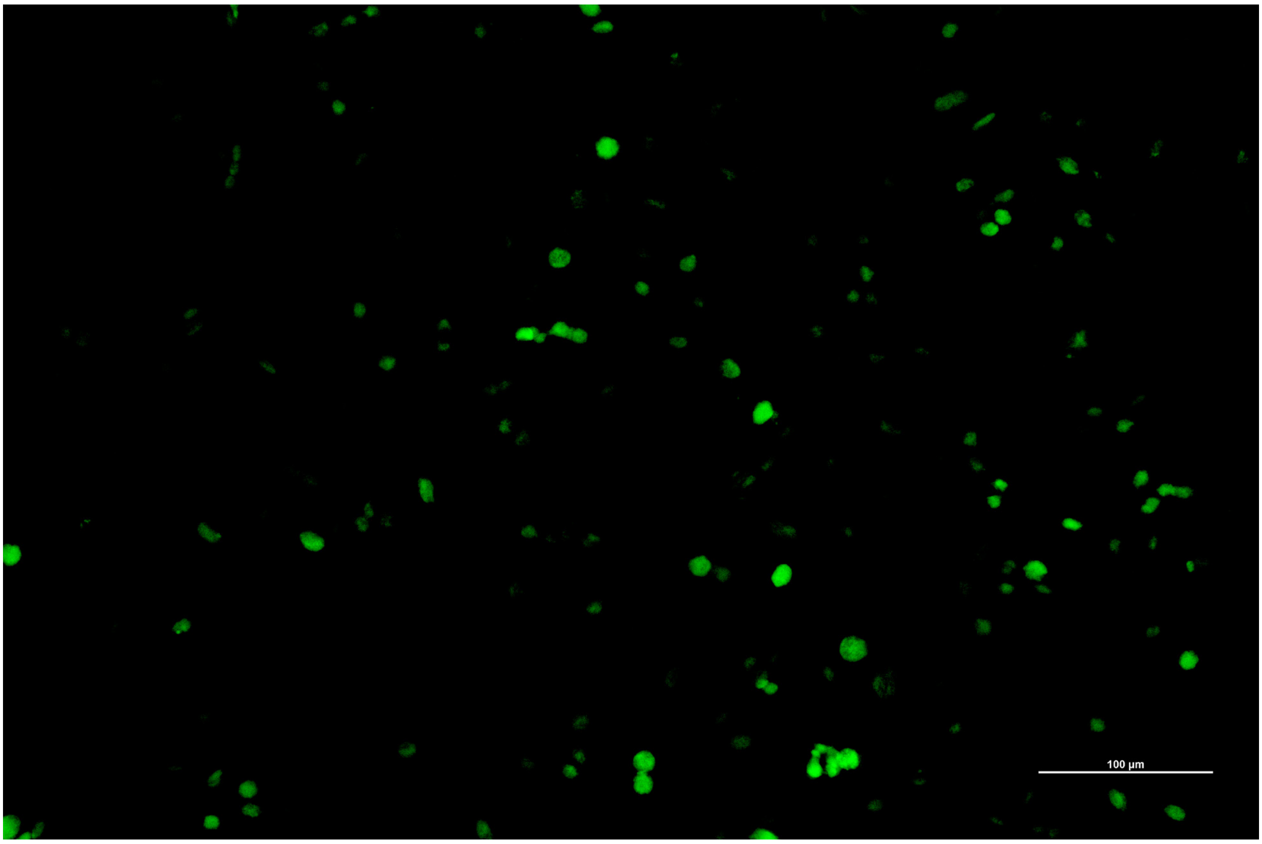

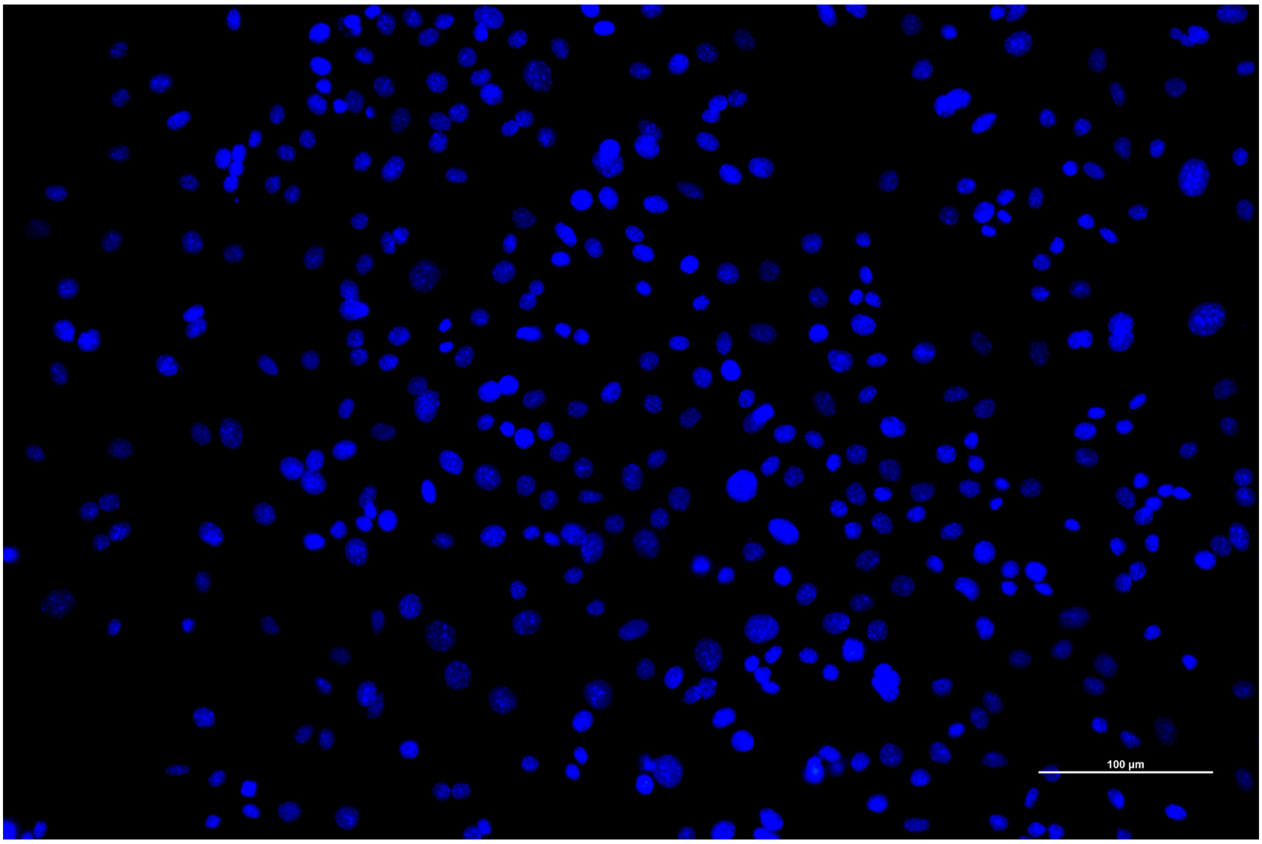

Figure S5. Original image of EdU (green) and DAPI (blue) staining in irradiated OCYs; Magnification = 200×.

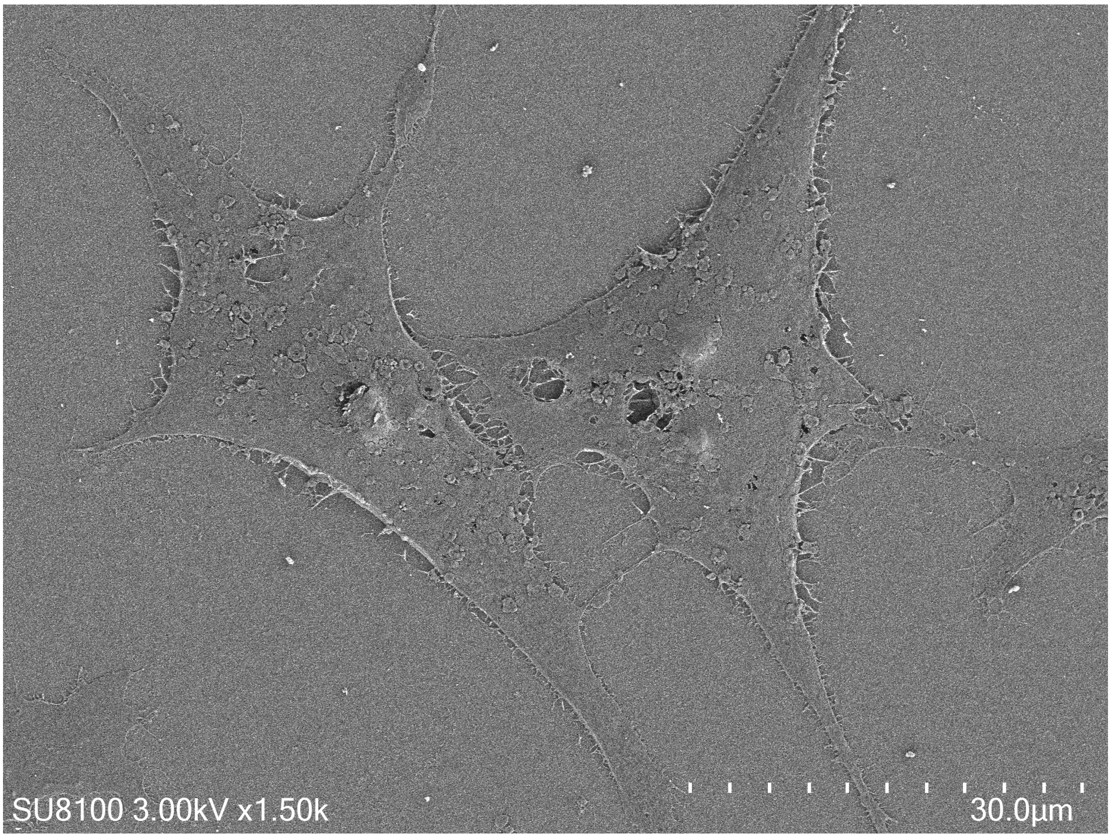

Figure S6. Original image of primary OCYs morphology under SEM; Magnification = 1500×.

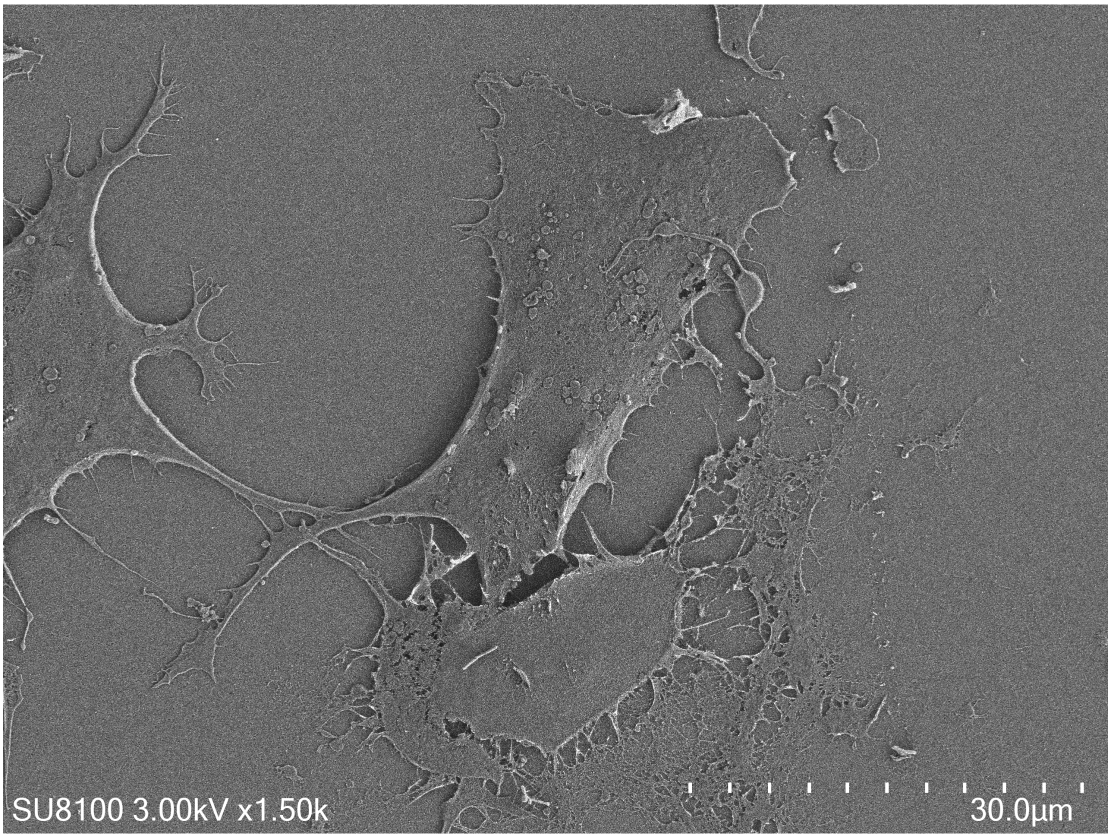

Figure S7. Original image of irradiated OCYs morphology under SEM; Magnification = 1500×.

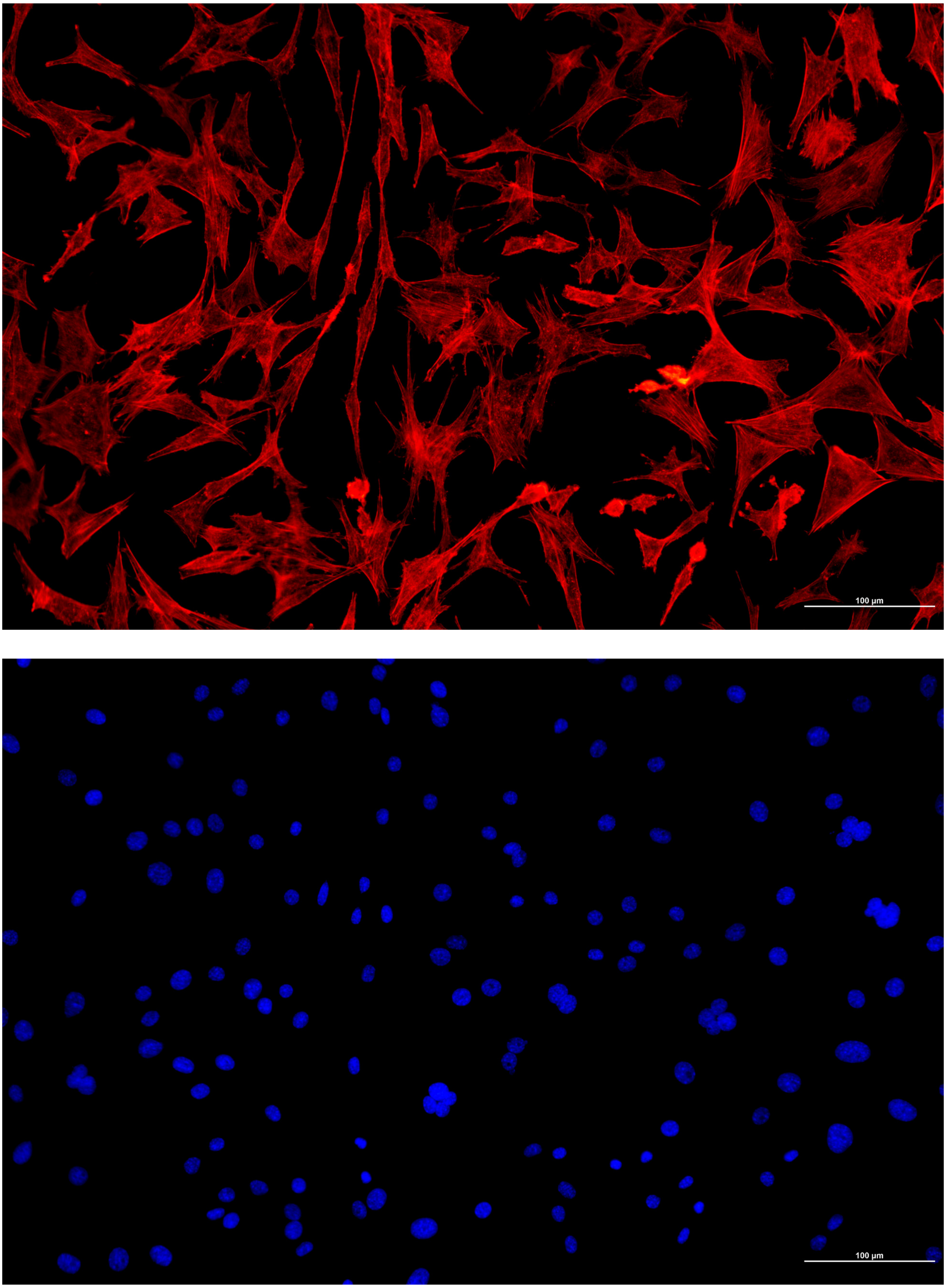

Figure S8. Immunostaining of primary OCYs using phalloidin-AlexaFluor488 and DAPI to visualize the cytoskeleton (red) and the nuclei (blue), respectively; Magnification = 200×.

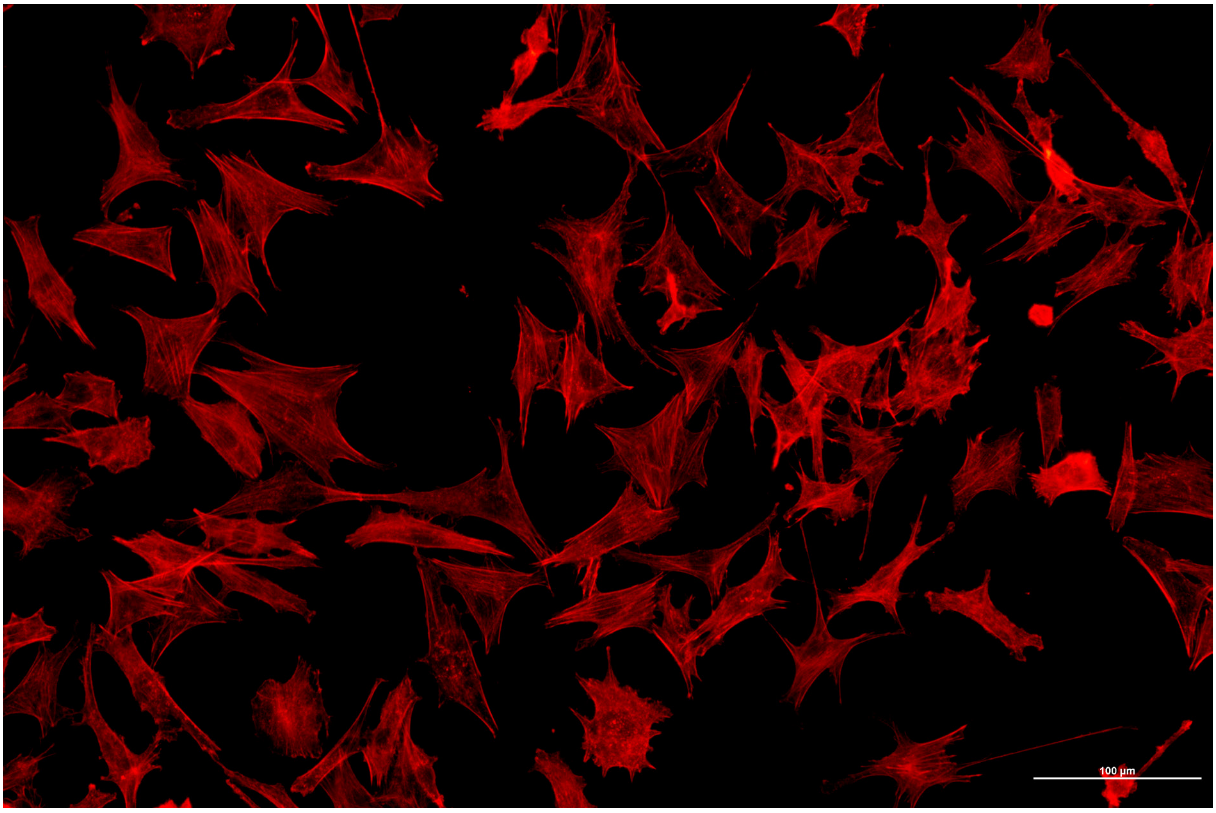

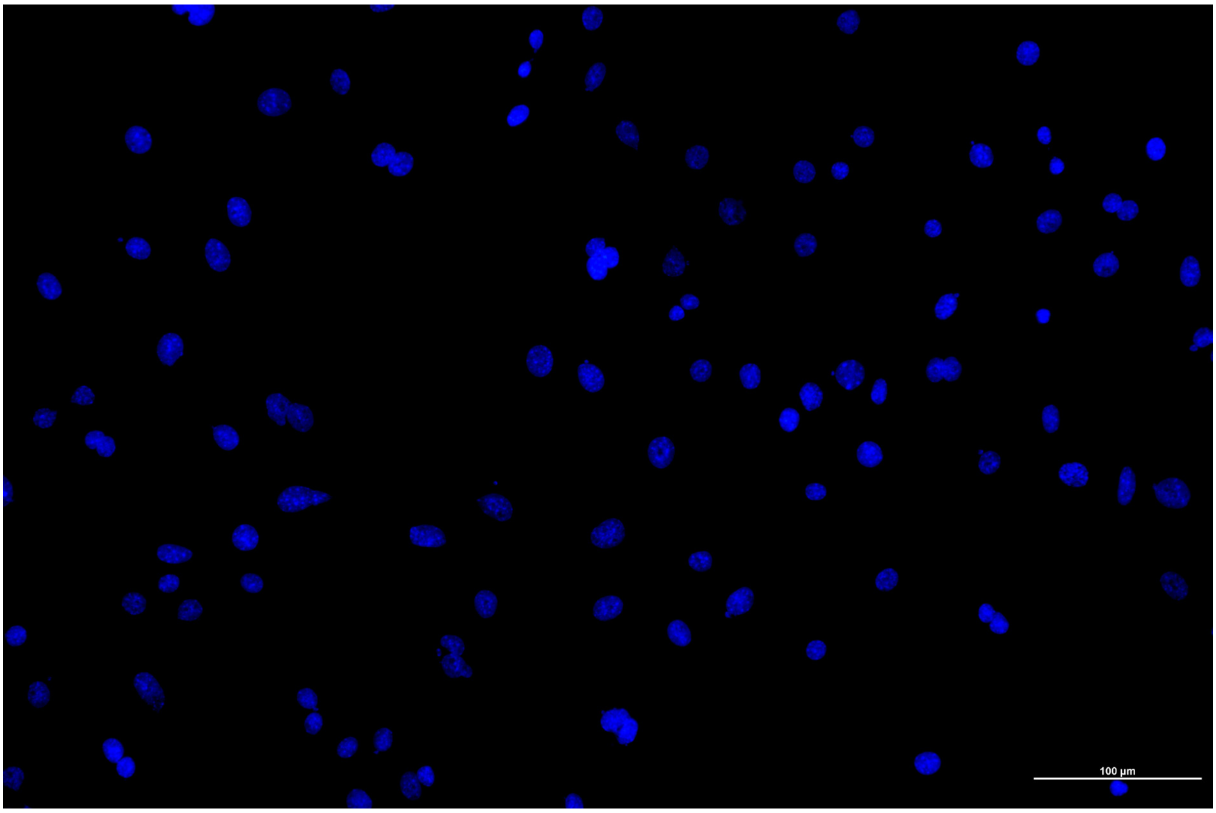

Figure S9. Immunostaining of irradiated OCYs using phalloidin-AlexaFluor488 and DAPI to visualize the cytoskeleton (red) and the nuclei (blue), respectively; Magnification = 200×.

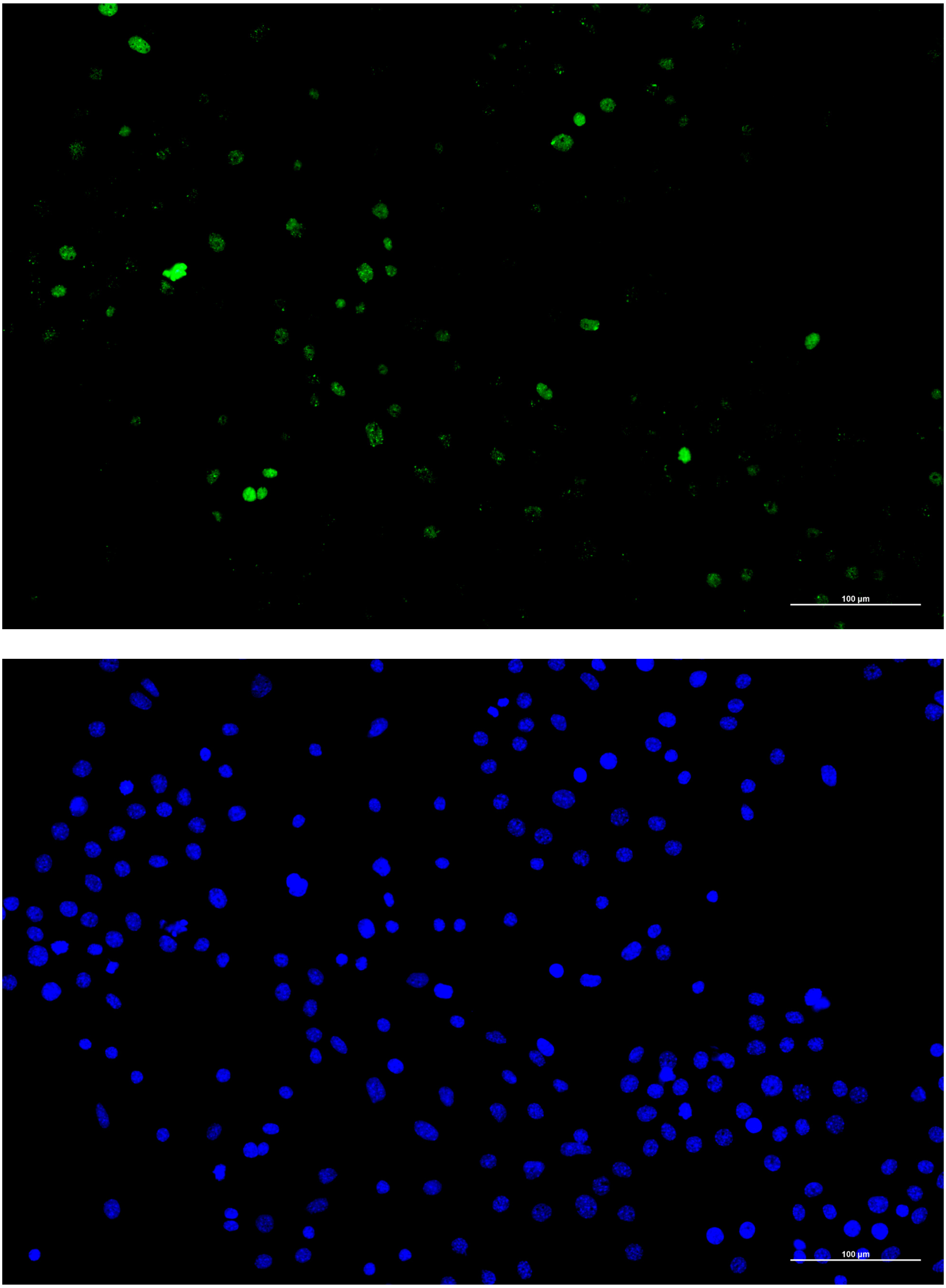

Figure S10. Immunofluorescence staining for γ- H2AX in primary OCYs: γ- H2AX (green) and DAPI (blue); Magnification = 200×.

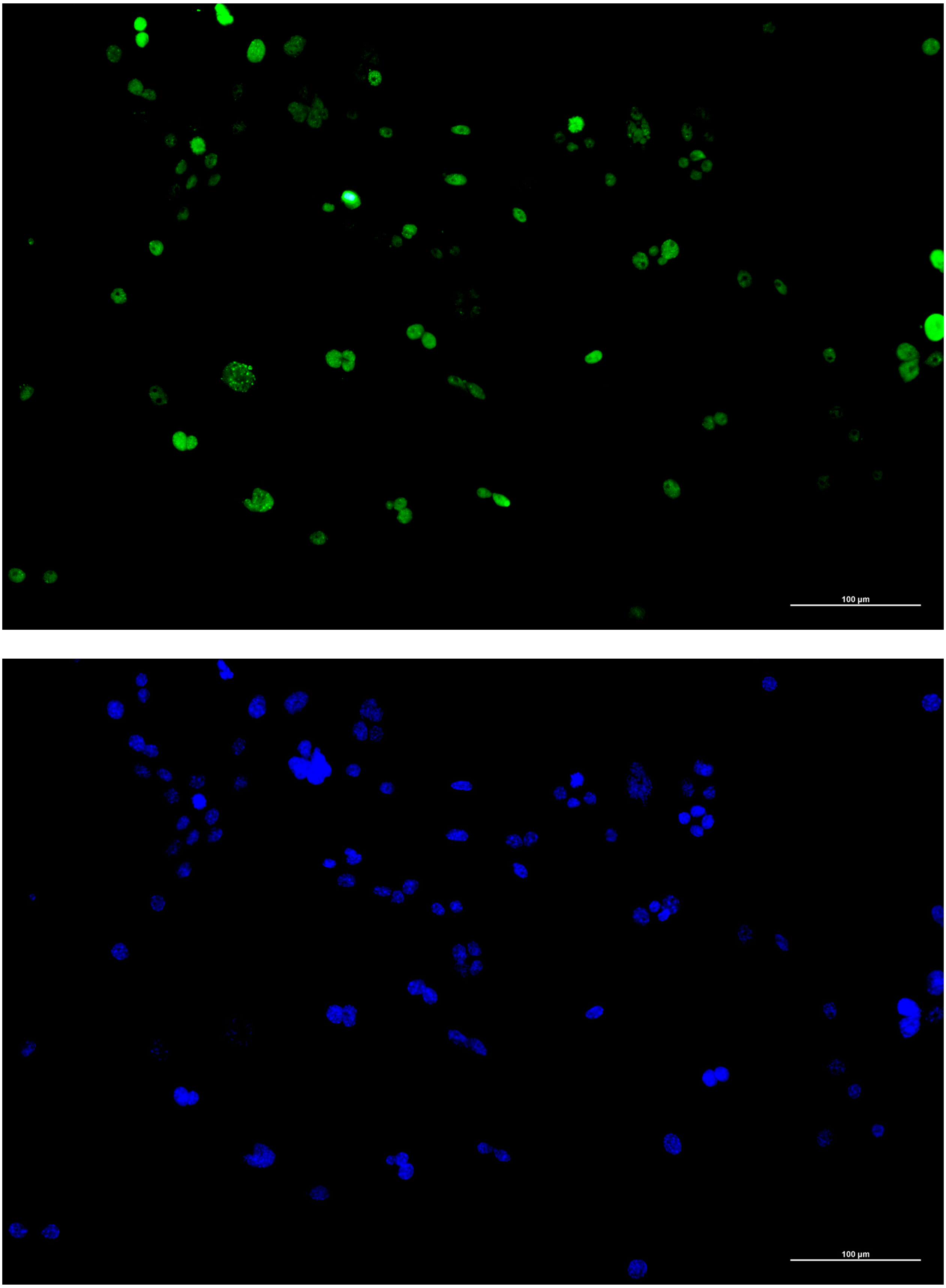

Figure S11. Immunofluorescence staining for γ- H2AX in irradiated OCYs: γ- H2AX (green) and DAPI (blue); Magnification = 200×.

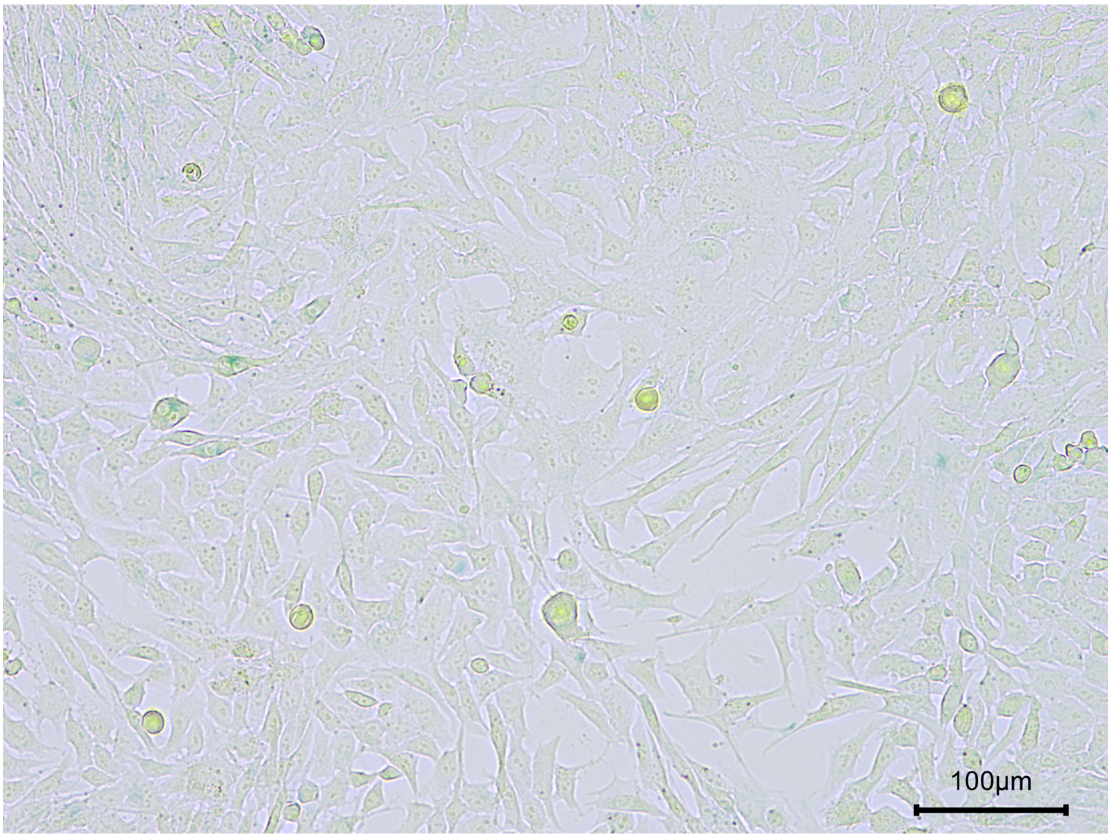

Figure S12. Original image of SA-β-gal staining in primary OCYs under LM; Magnification = 100×.

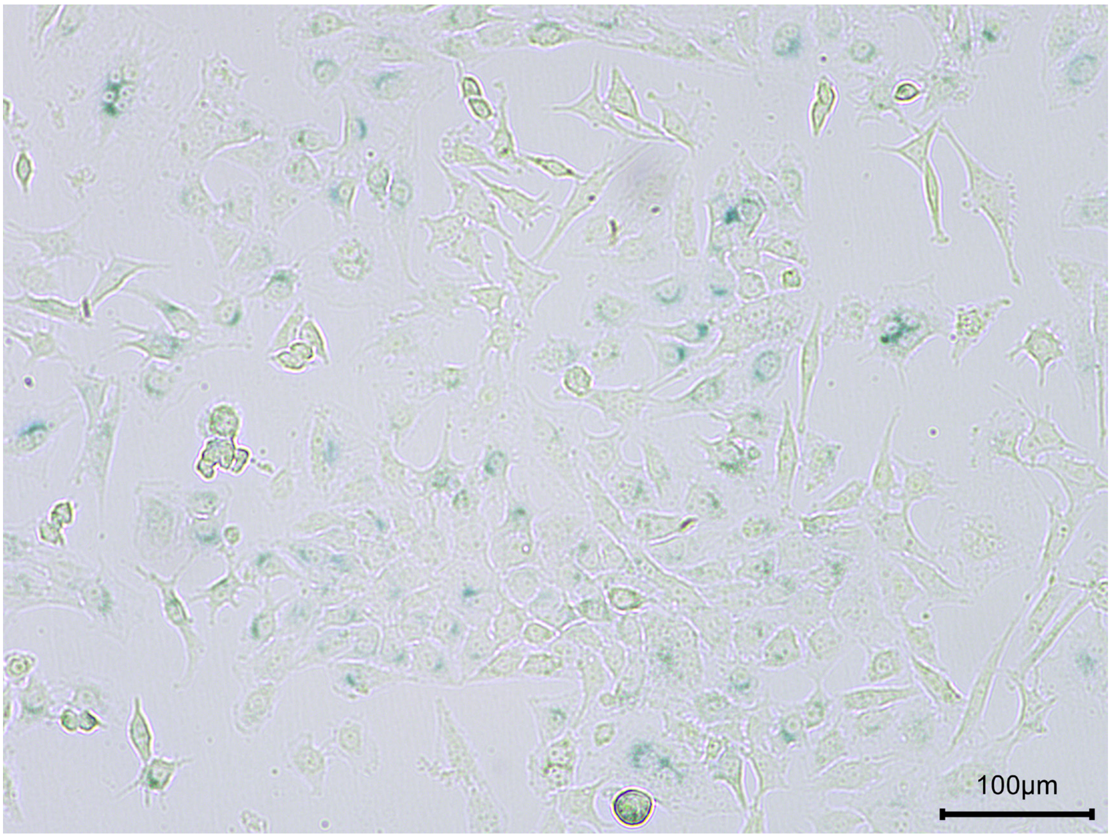

Figure S13. Original image of SA-β-gal staining in irradiated OCYs under LM; Magnification = 100×.

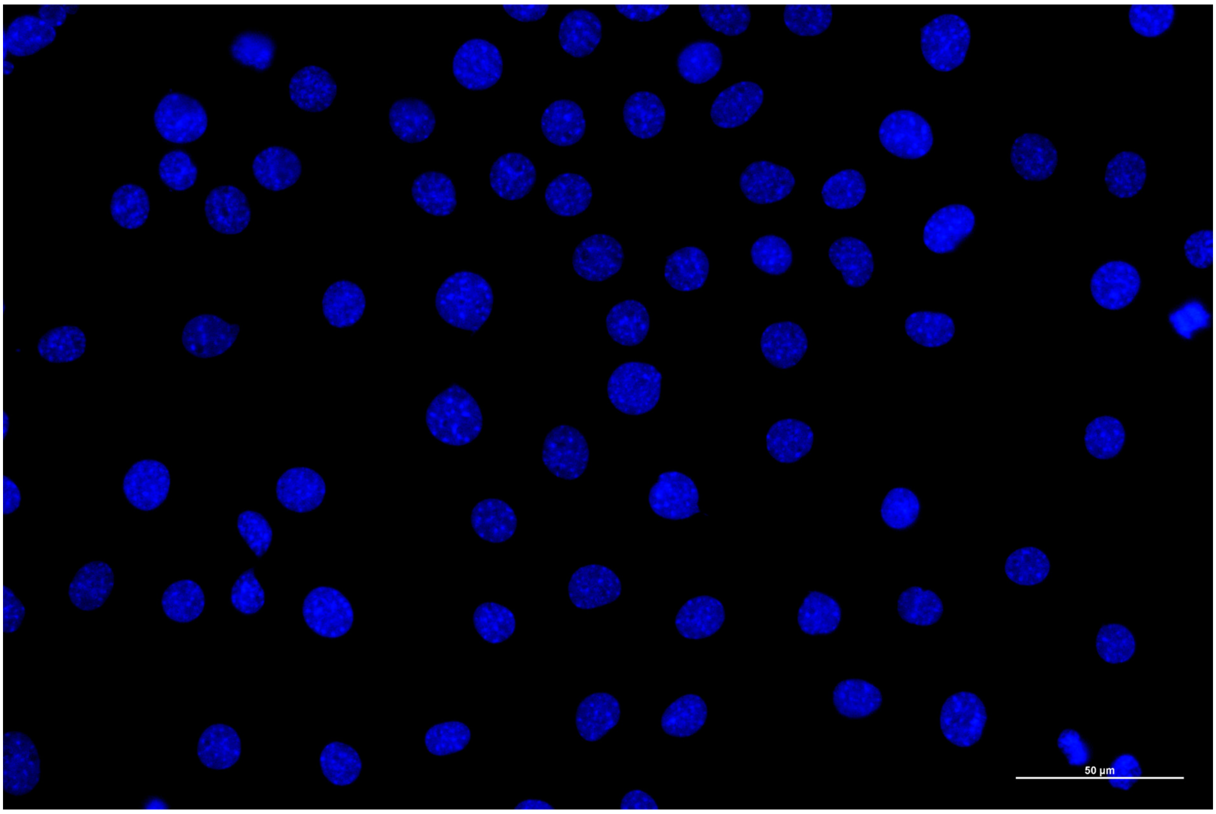

Figure S14. Original image of SAHFs formation in primary OCYs nuclei under FM; Magnification = 400×.

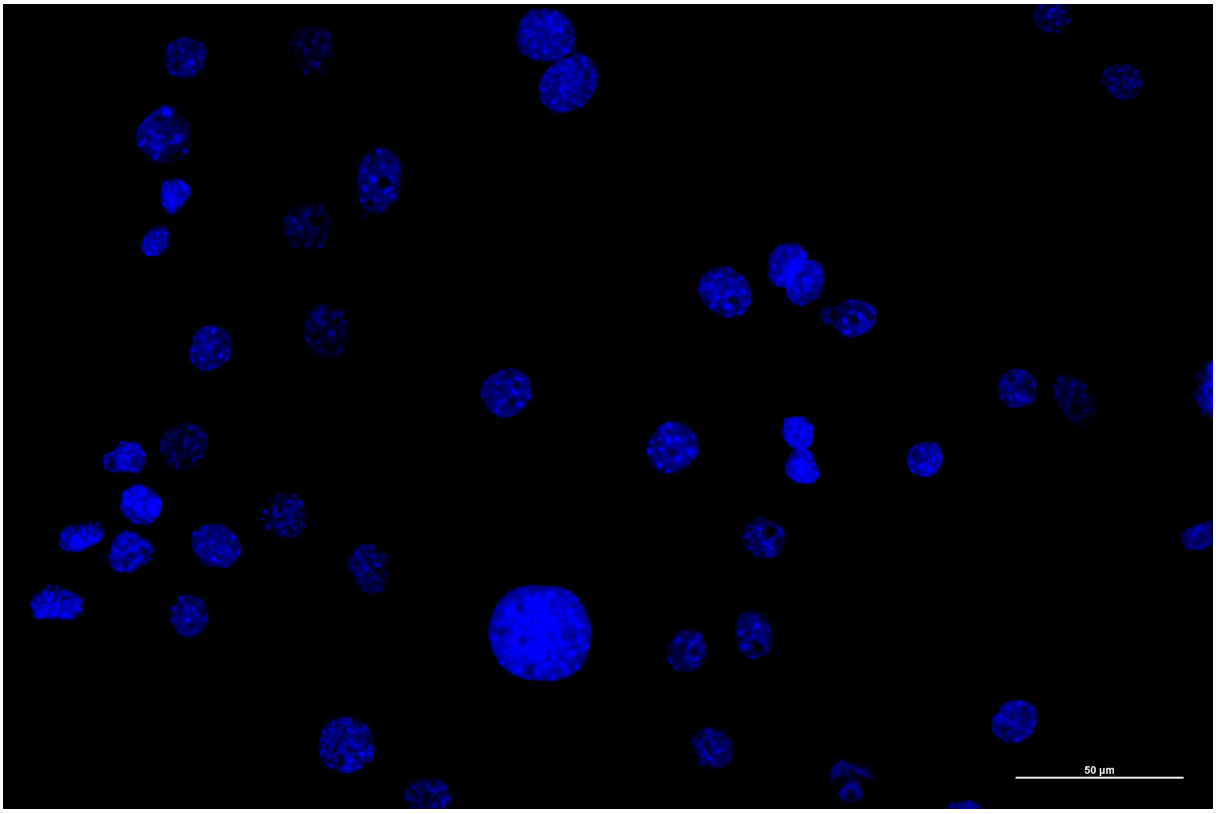

Figure S15. Original image of SAHFs formation in irradiated OCYs nuclei under FM; Magnification = 400×.

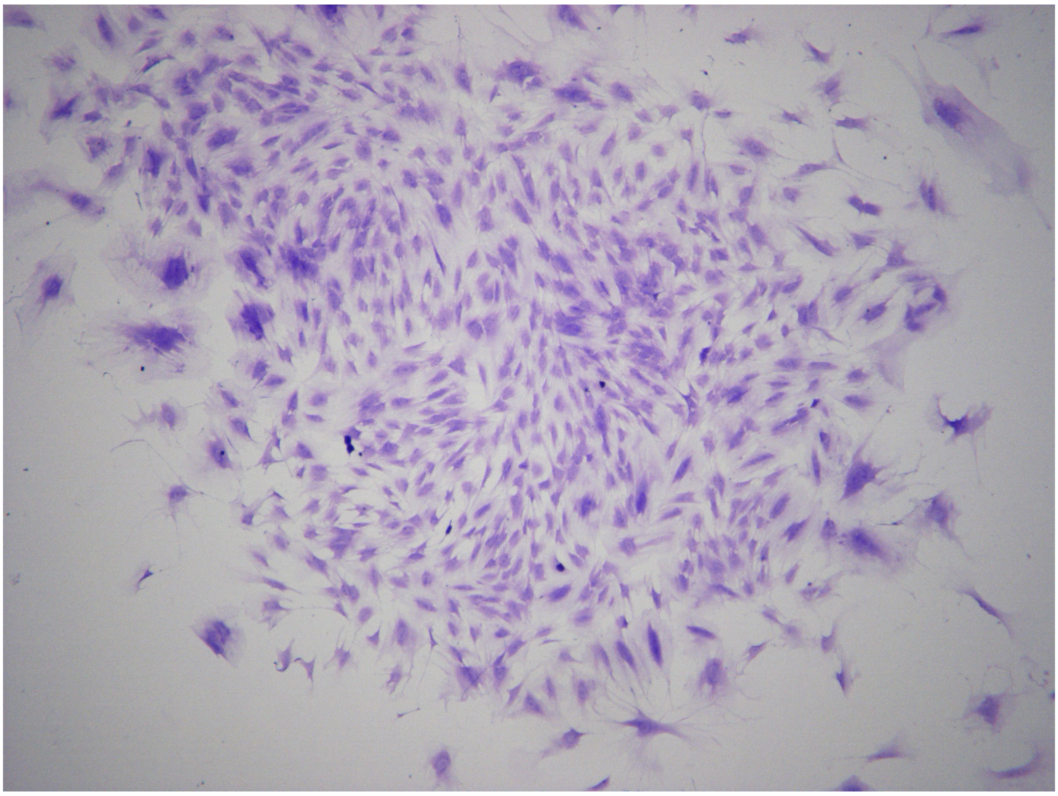

Figure S16. Original image of crystal violet staining in BMSCs co-cultured with 0 Gy-CM under LM; Magnification = 40×.

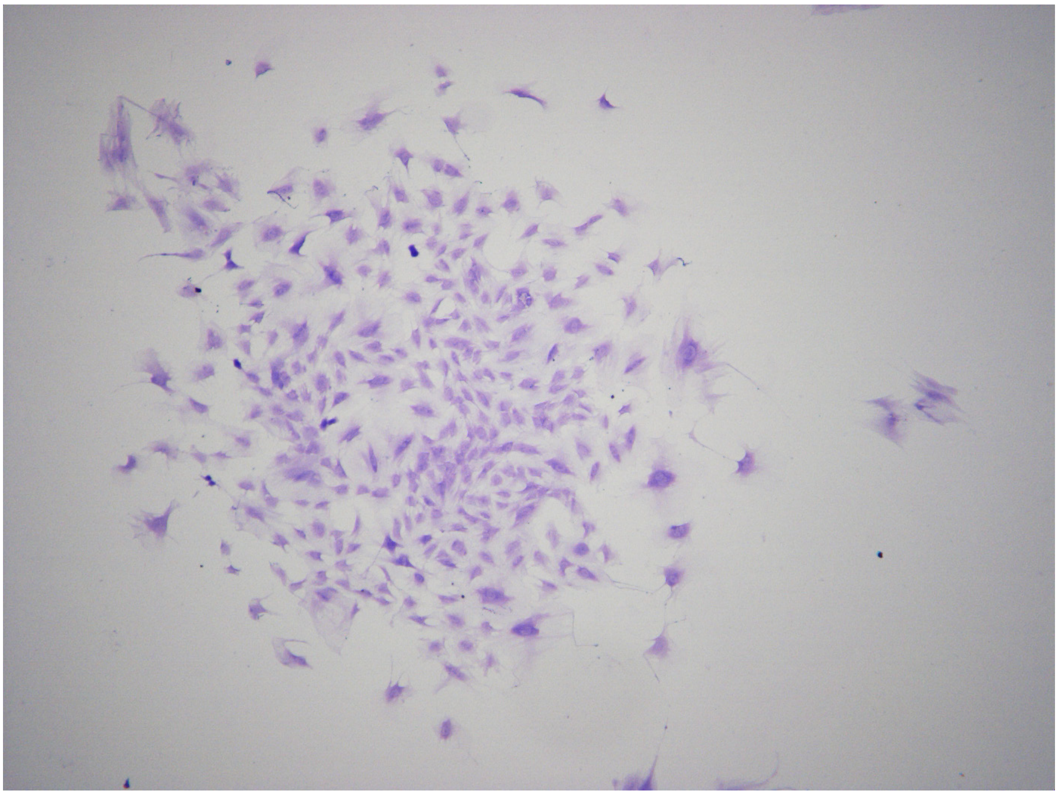

Figure S17. Original image of crystal violet staining in BMSCs co-cultured with 2 Gy-CM under LM; Magnification = 40×.

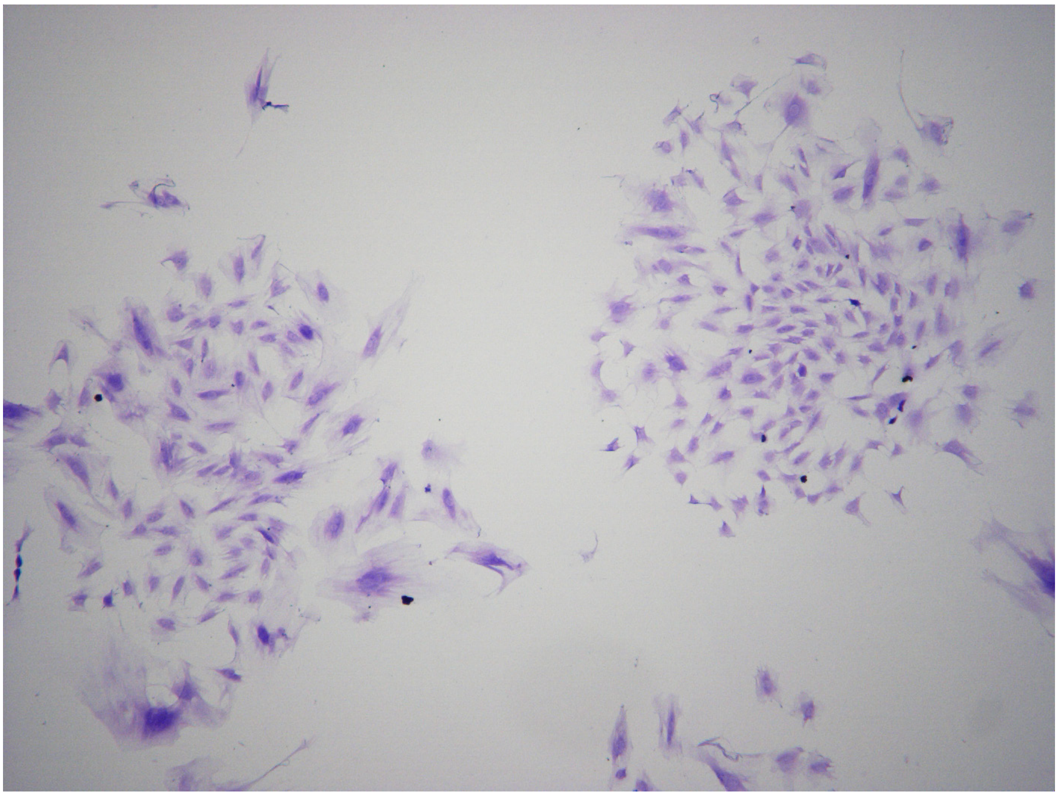

Figure S18. Original image of crystal violet staining in BMSCs co-cultured with 2 Gy-CM + anti-CCL3 under LM; Magnification = 40×.

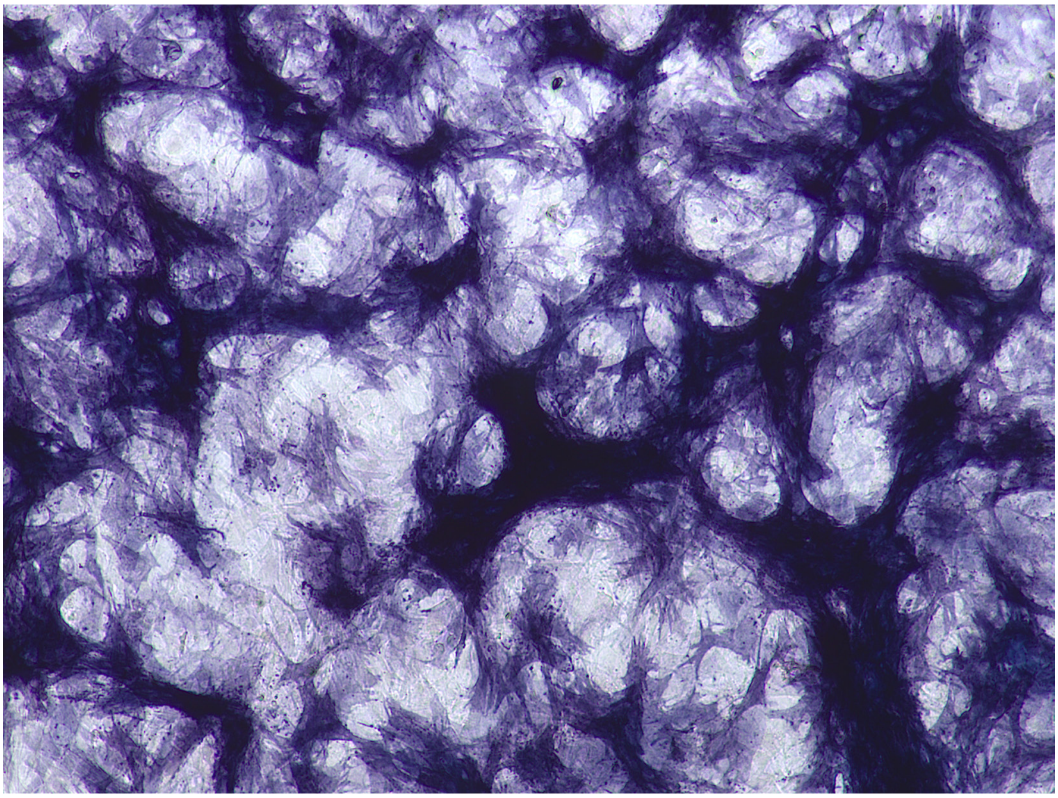

Figure S19. Original image of ALP staining in BMSCs co-cultured with 0 Gy-CM under LM; Magnification = 40×.

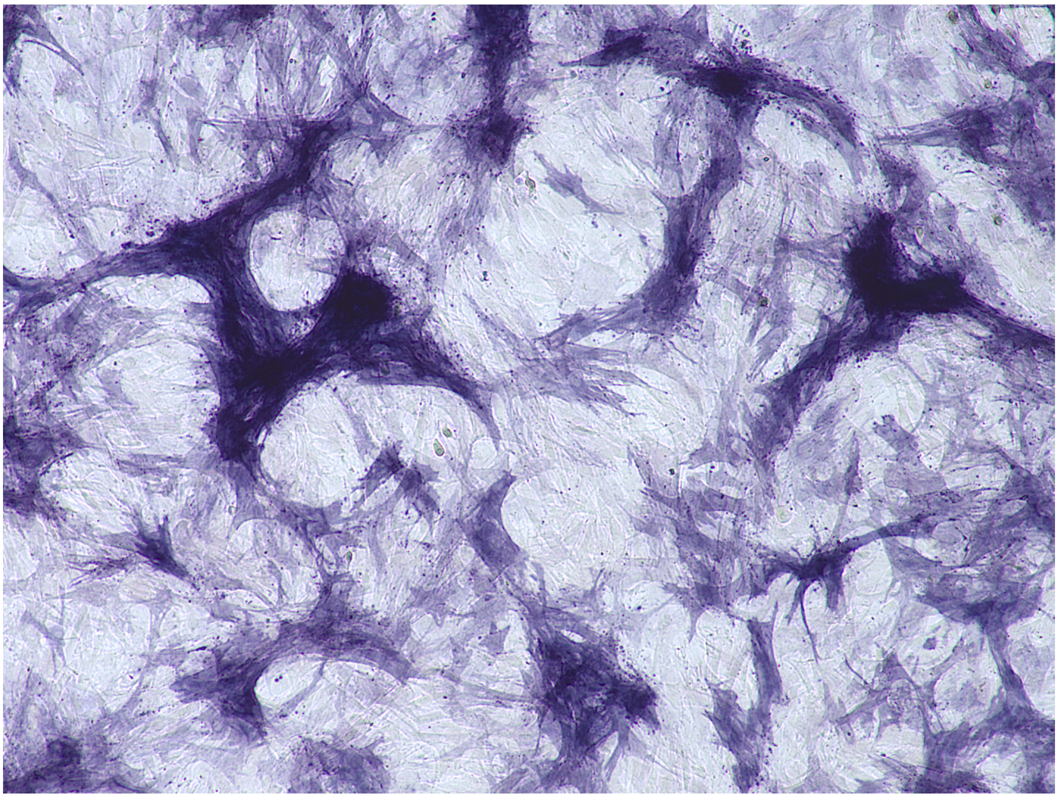

Figure S20. Original image of ALP staining in BMSCs co-cultured with 2 Gy-CM under LM; Magnification = 40×.

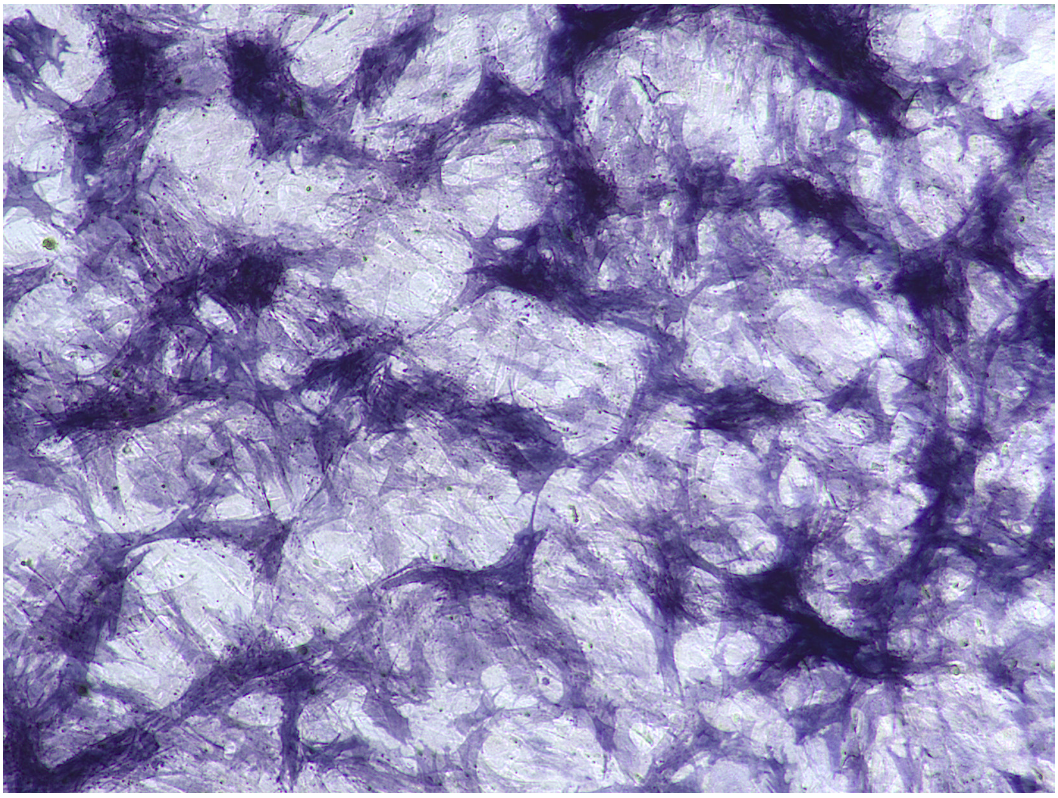

Figure S21. Original image of ALP staining in BMSCs co-cultured with2 Gy-CM + anti-CCL3 under LM; Magnification = 40×.

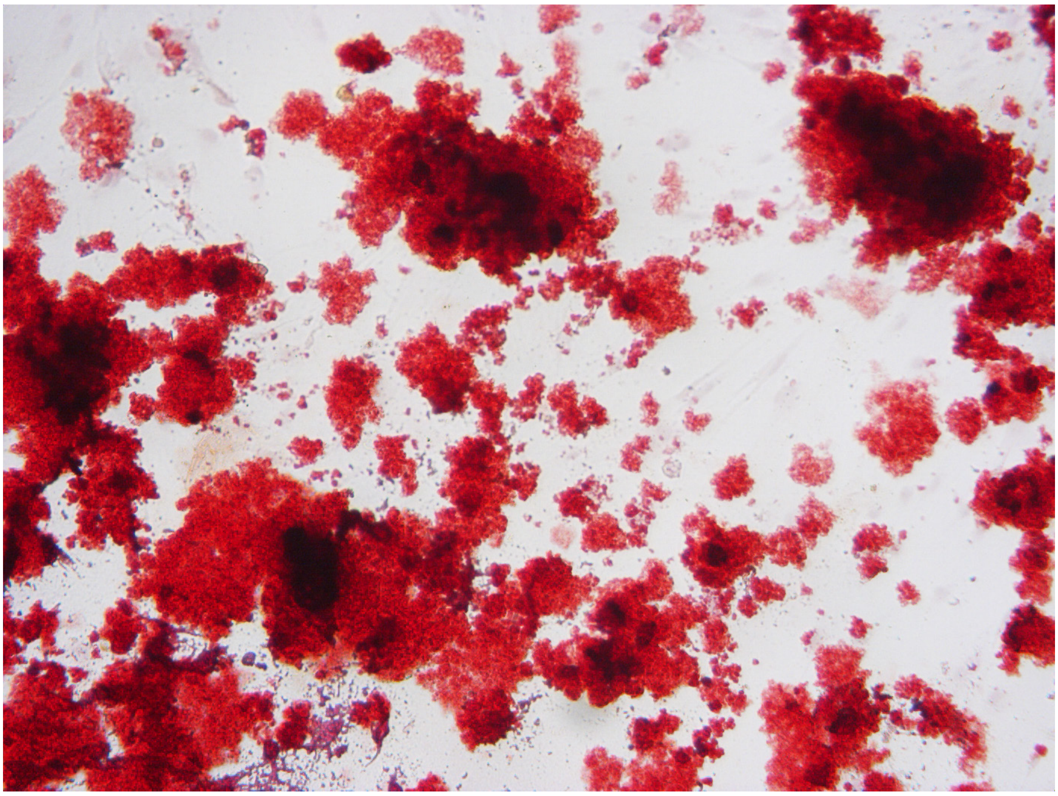

Figure S22. Original image of alizarin red staining in BMSCs co-cultured with 0 Gy-CM under LM; Magnification = 100×.

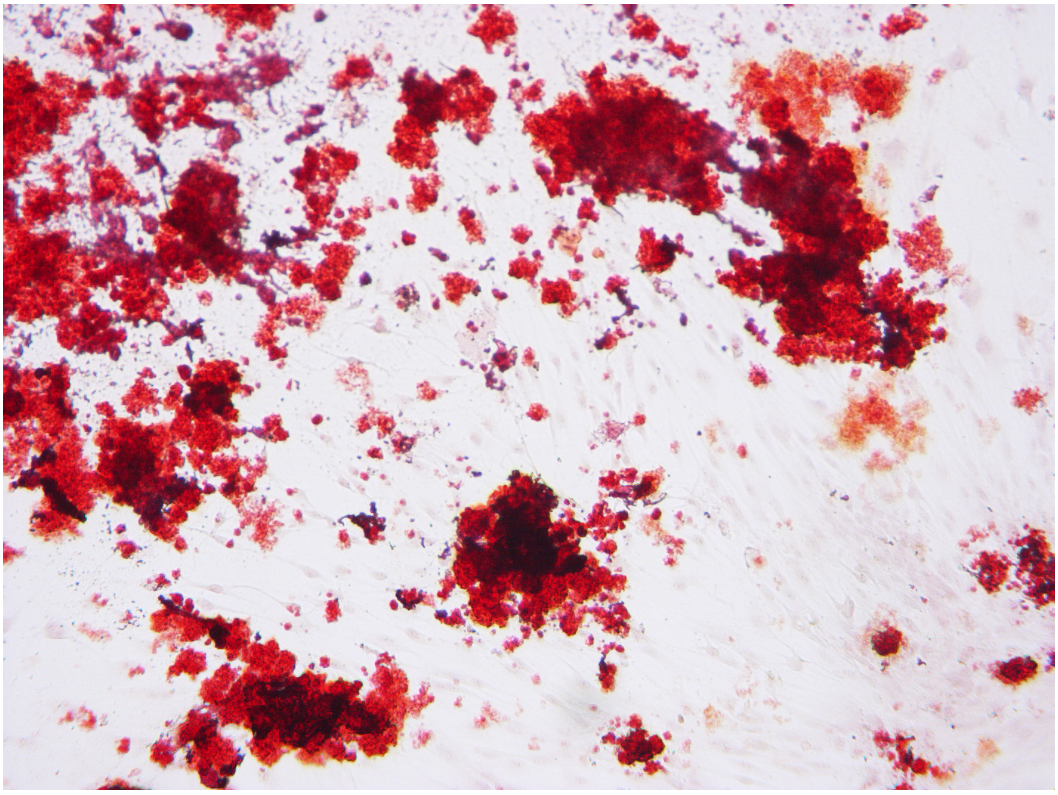

Figure S23. Original image of alizarin red staining in BMSCs co-cultured with 2 Gy-CM under LM; Magnification = 100×.

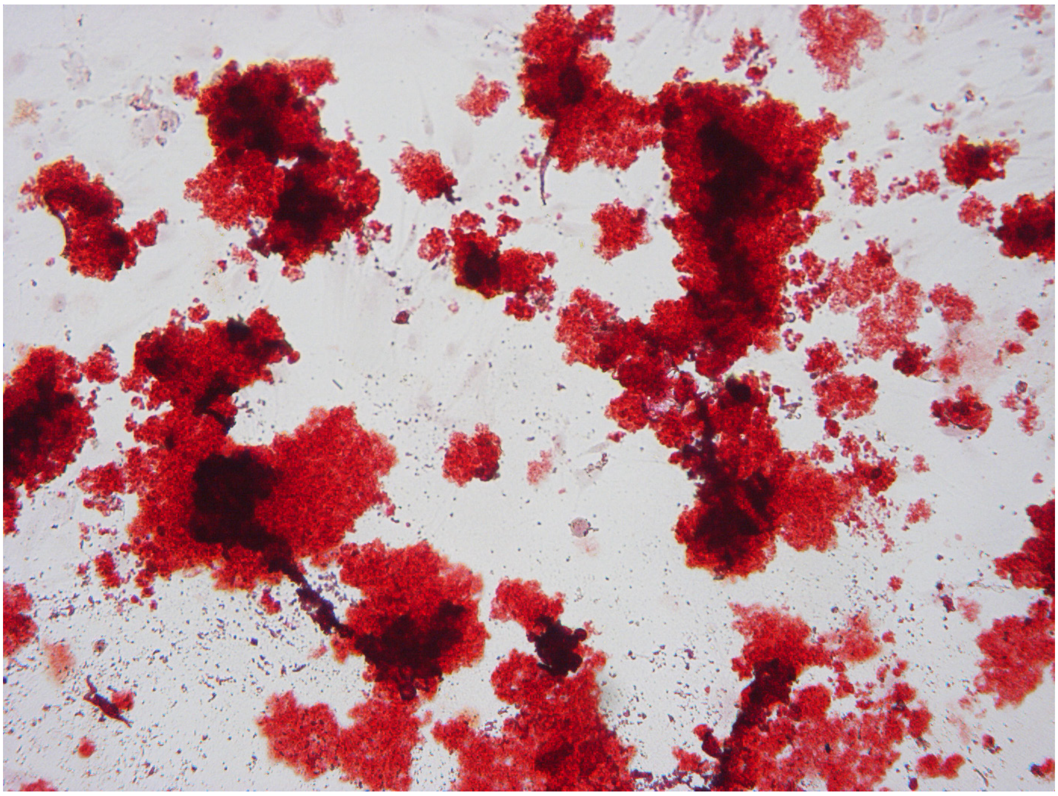

Figure S24. Original image of alizarin red staining in BMSCs co-cultured with 2 Gy-CM + anti-CCL3 under LM; Magnification = 100×.

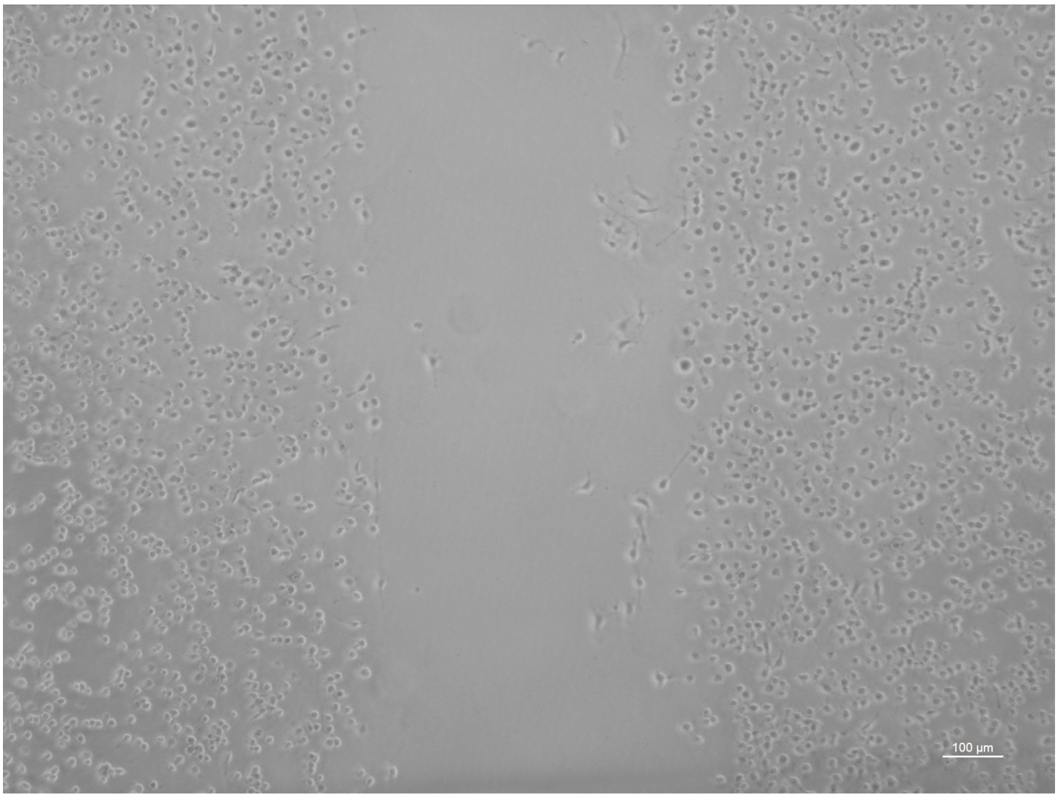

Figure S25. Original image of wound-healing scratch assay in osteoclast precursor cells co-cultured with 0 Gy-CM under inverted phase-contrast optical microscope; Magnification = 50×.

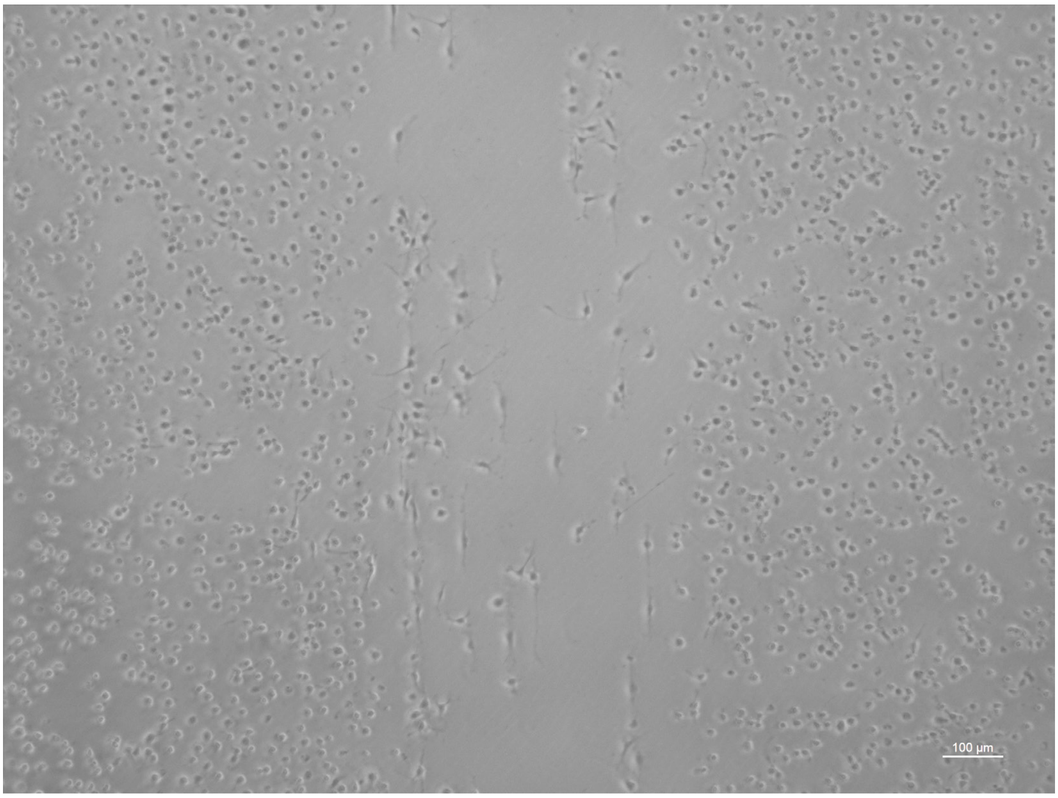

Figure S26. Original image of wound-healing scratch assay in osteoclast precursor cells co-cultured with 2 Gy-CM under inverted phase-contrast optical microscope; Magnification = 50×.

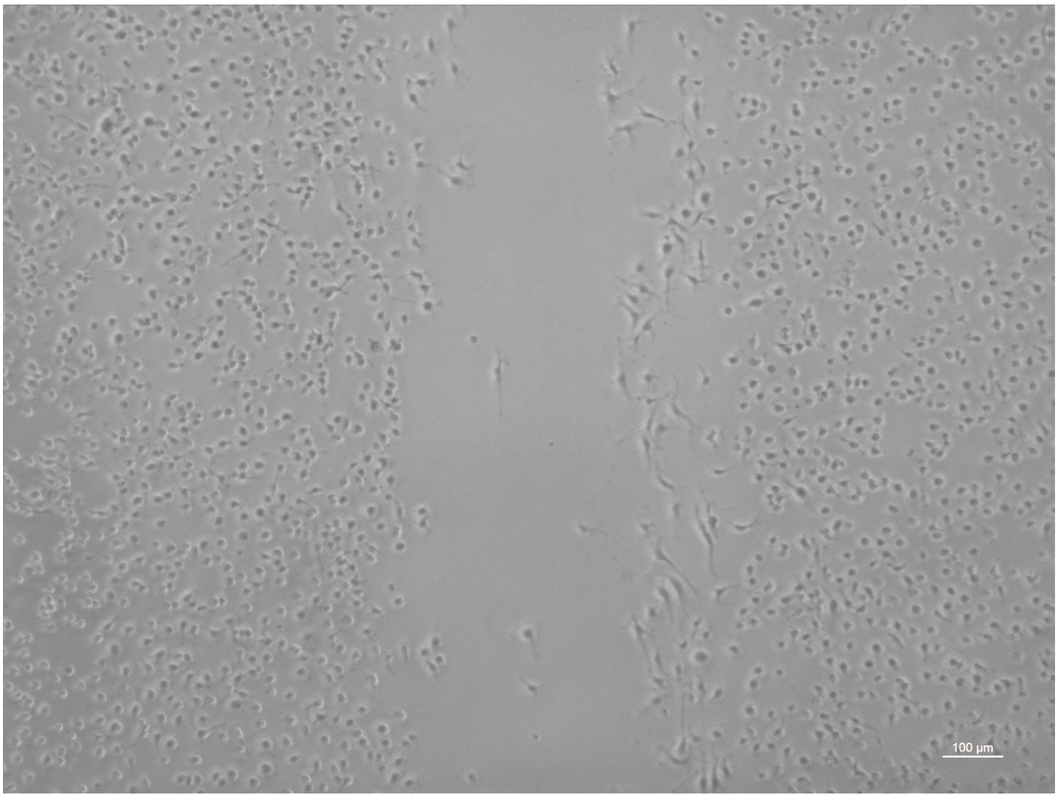

Figure S27. Original image of wound-healing scratch assay in osteoclast precursor cells co-cultured with 2 Gy-CM + anti-CCL3 under inverted phase-contrast optical microscope; Magnification = 50×.

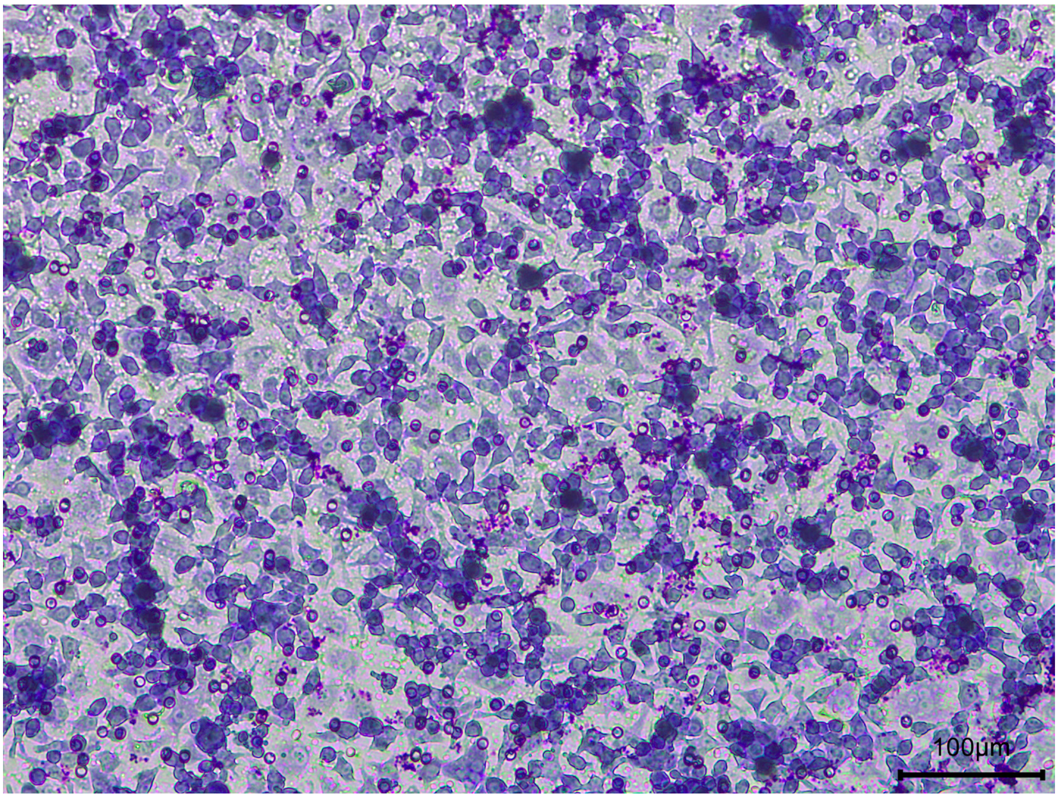

Figure S28. Original image of trans-well assay in osteoclast precursor cells co-cultured with 0 Gy-CM under LM; Magnification = 100×.

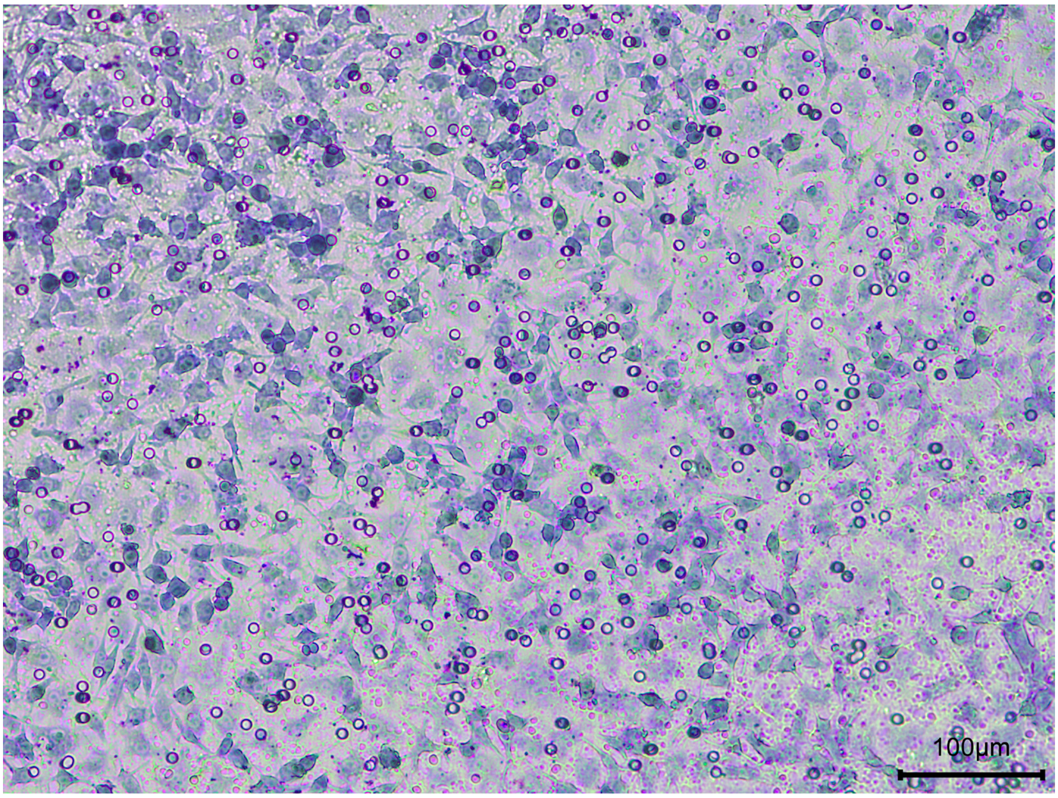

Figure S29. Original image of trans-well assay in osteoclast precursor cells co-cultured with 2 Gy-CM under LM; Magnification = 100×.

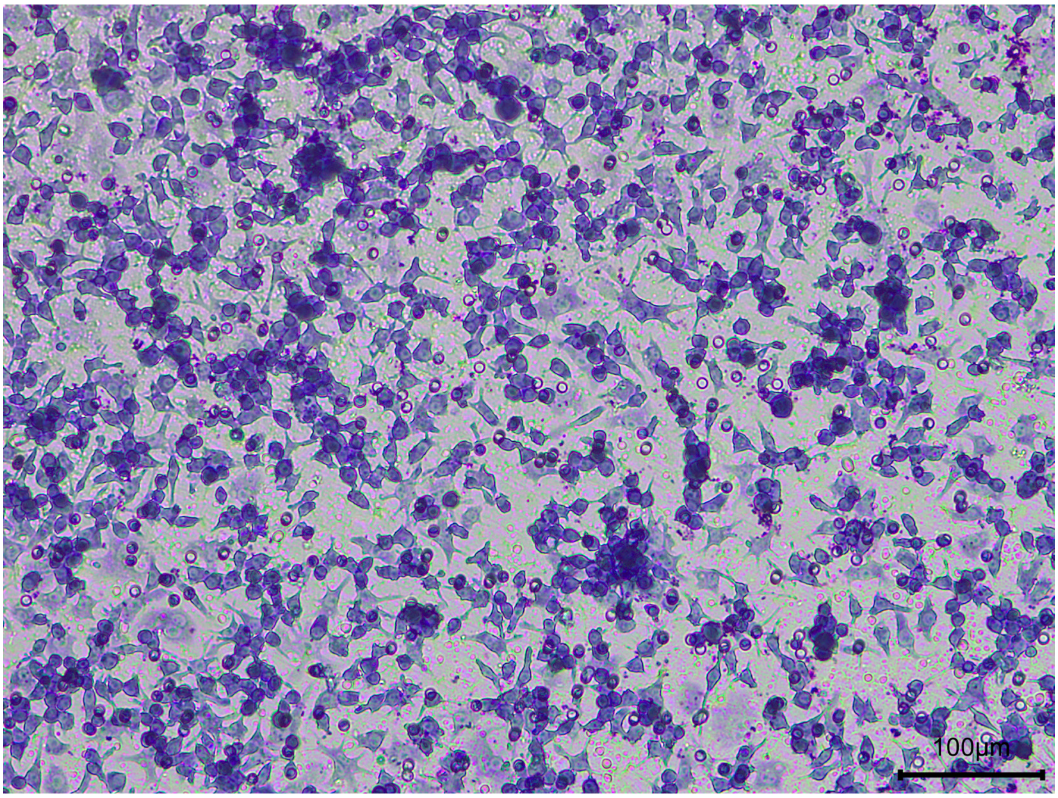

Figure S30. Original image of trans-well assay in osteoclast precursor cells co-cultured with 2 Gy-CM + anti-CCL3 under LM; Magnification = 100×.

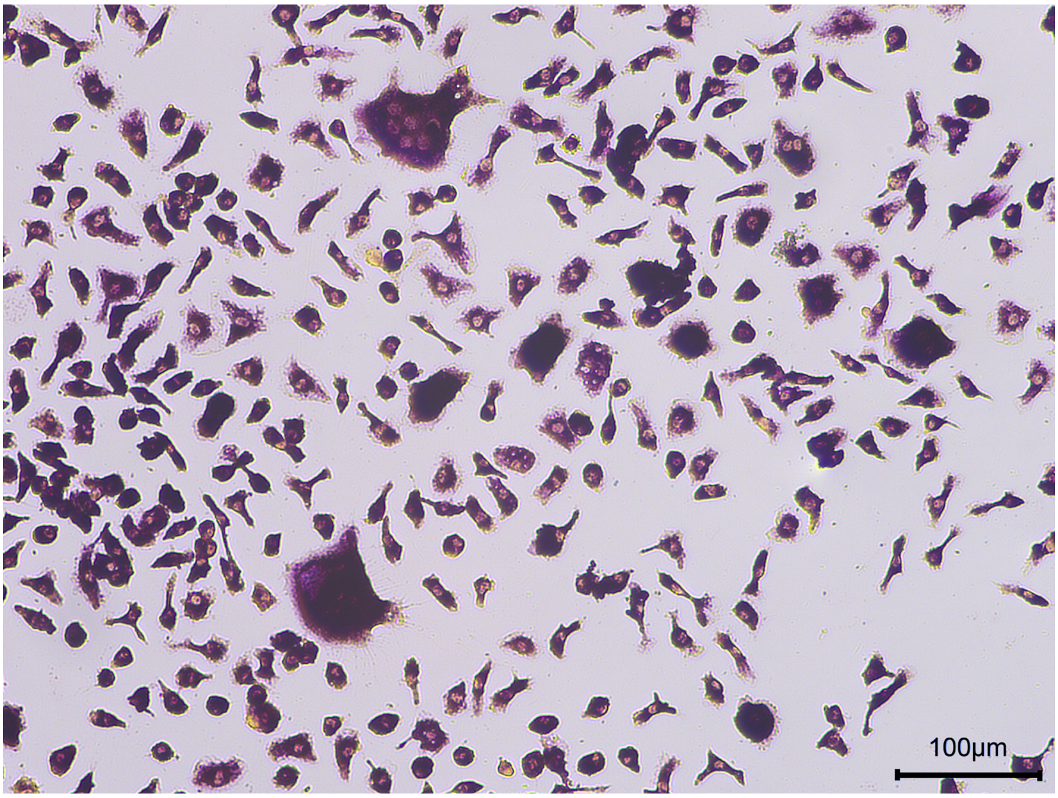

Figure S31. Original image of TRAP staining in OCs co-cultured with 0 Gy-CM under LM; Magnification = 100×.

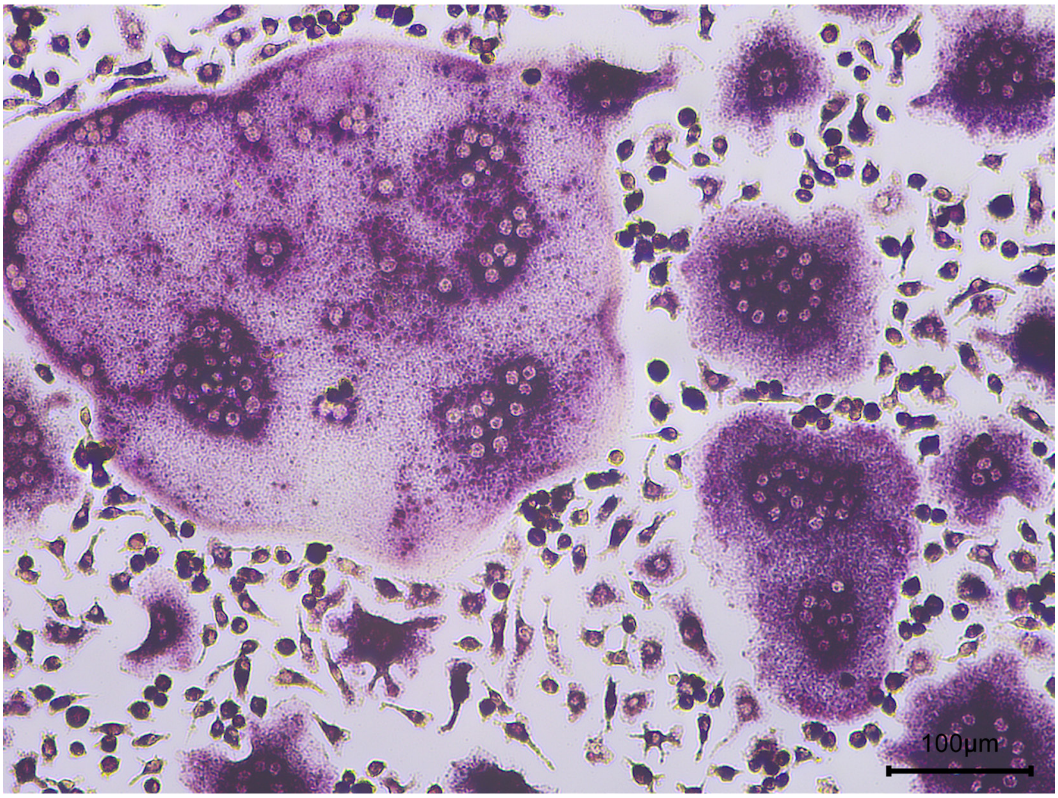

Figure S32. Original image of TRAP staining in OCs co-cultured with 2 Gy-CM under LM; Magnification = 100×.

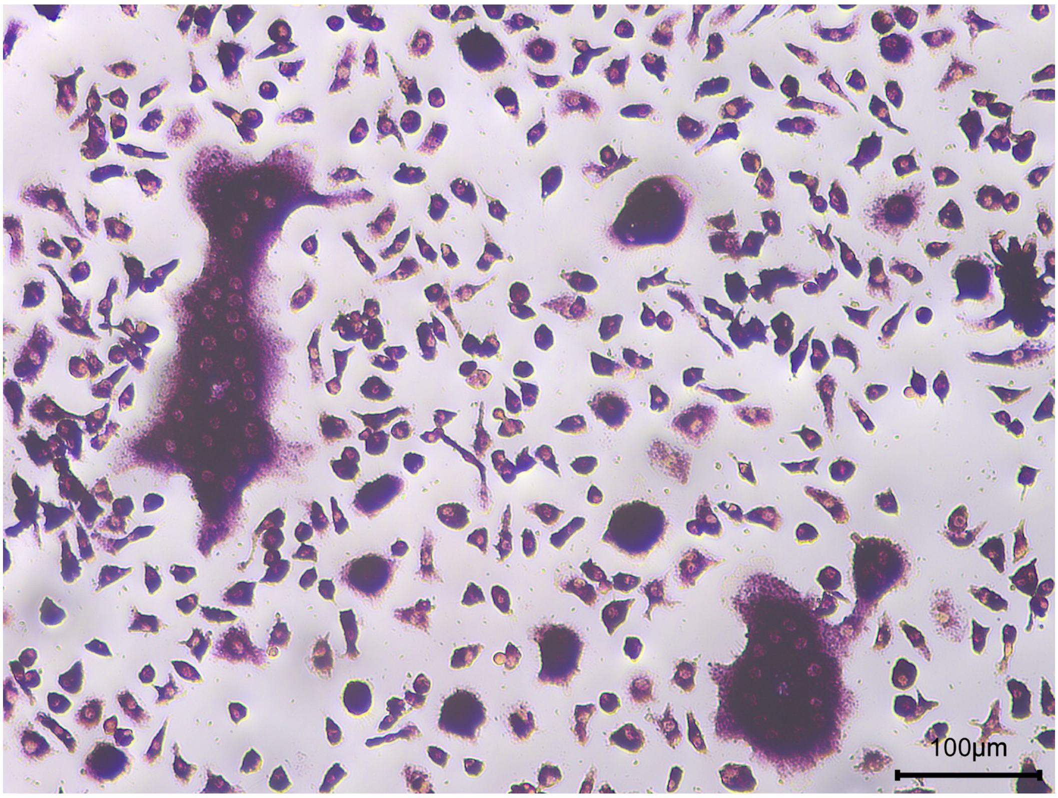

Figure S33. Original image of TRAP staining in OCs co-cultured with 2 Gy-CM + anti-CCL3 under LM; Magnification = 100×.

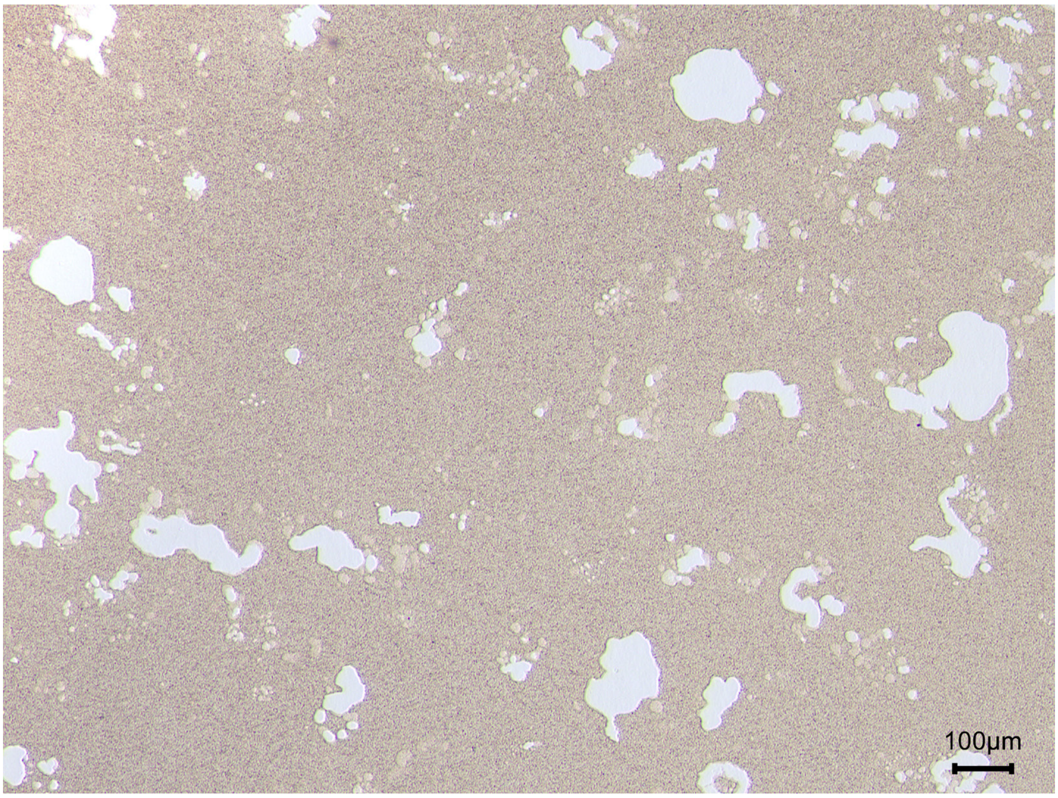

Figure S34. Original image of bone resorption pits in OCs co-cultured with 0 Gy-CM under LM; Magnification = 40×

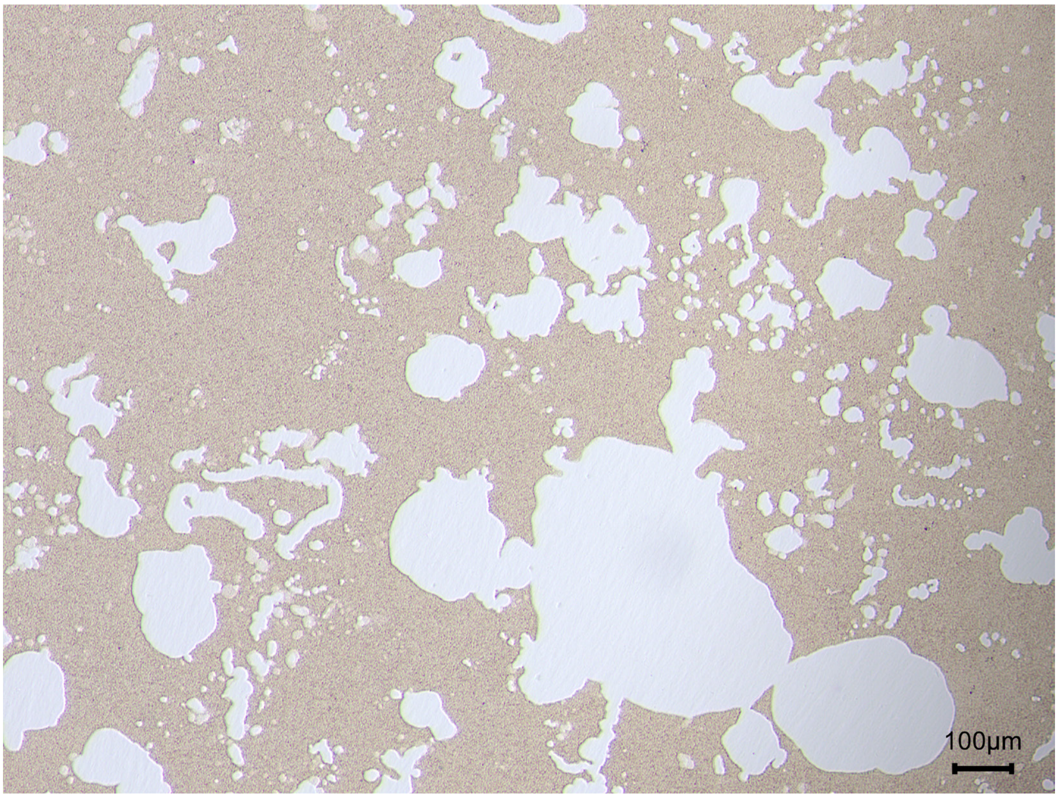

Figure S35. Original image of bone resorption pits in OCs co-cultured with 2 Gy-CM under LM; Magnification = 40×.

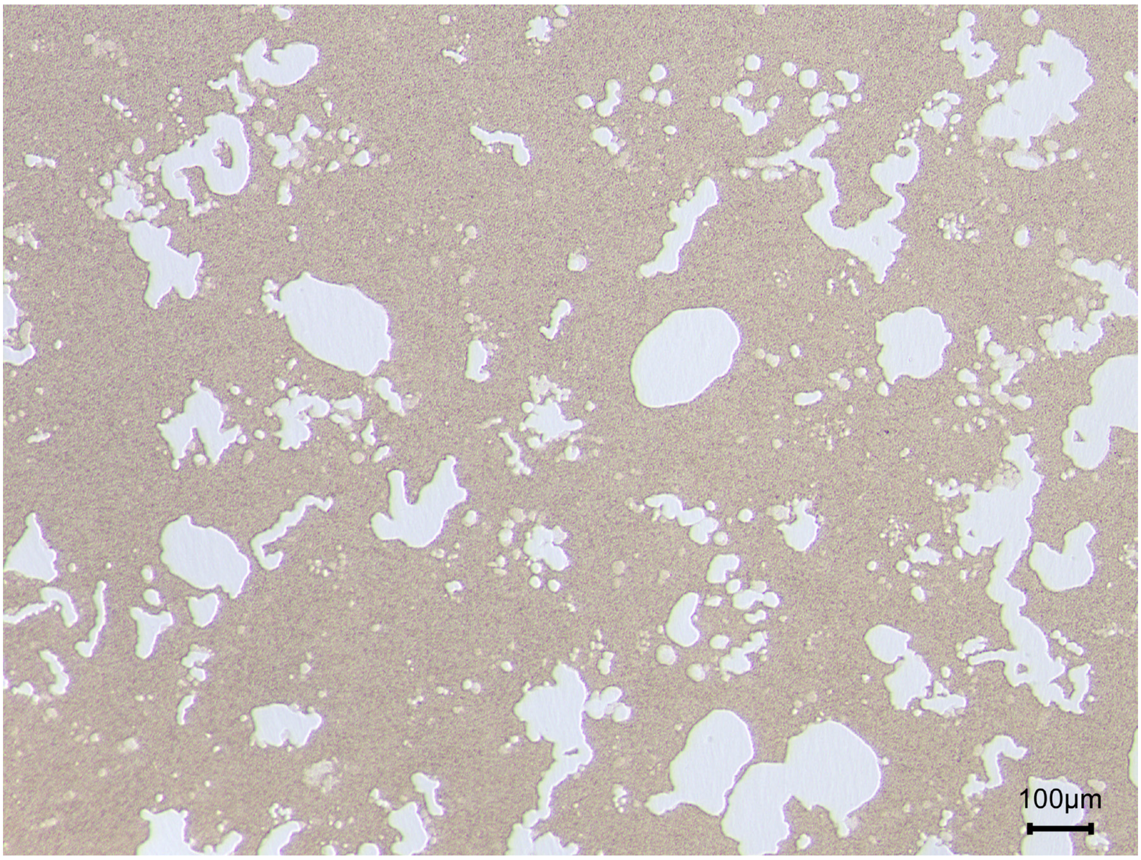

Figure S36. Original image of bone resorption pits in OCs co-cultured with 2 Gy-CM + anti-CCL3 under LM; Magnification = 40×.

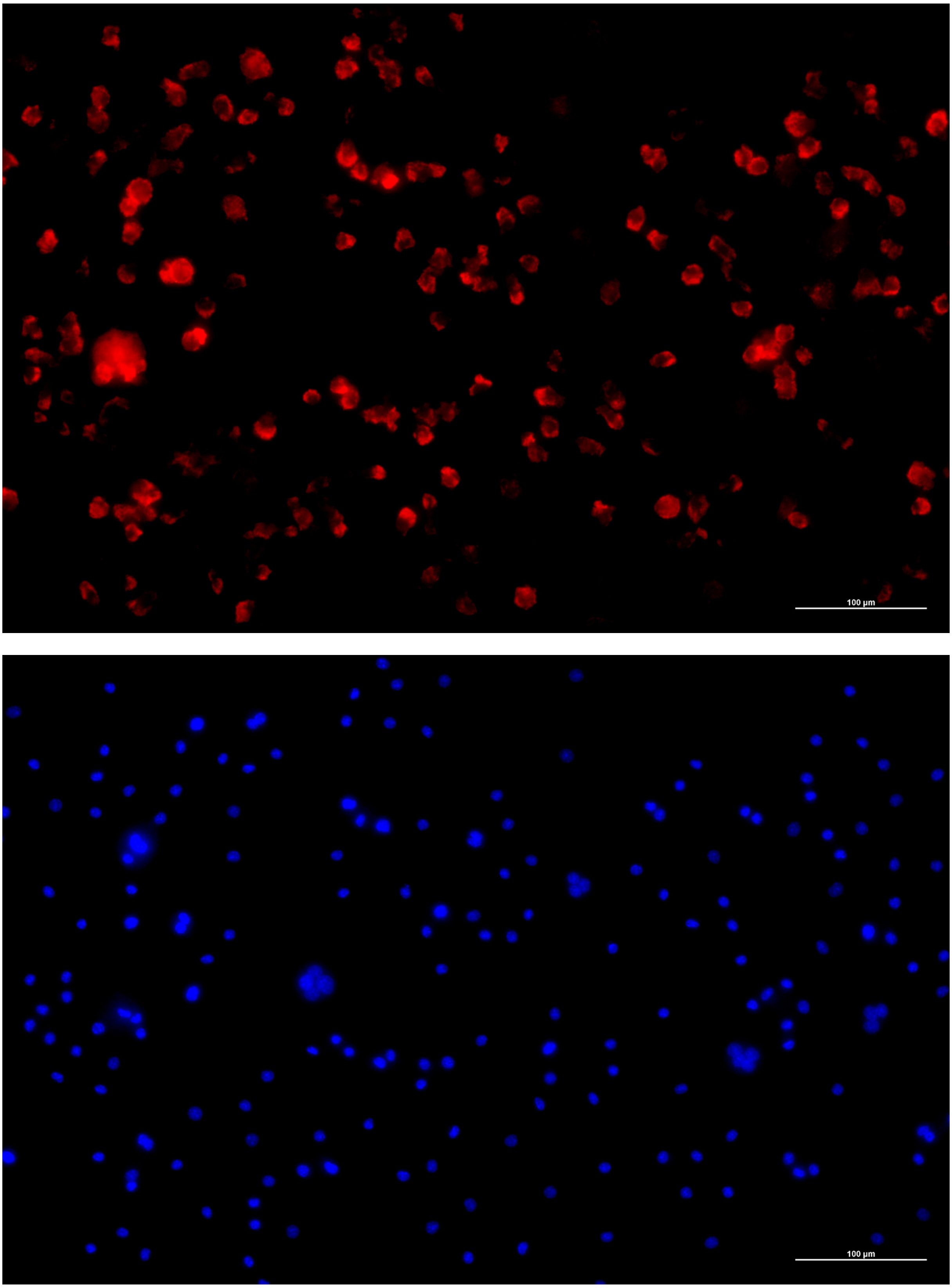

Figure S37. Immunostaining of the OCs co-cultured with 0 Gy-CM using phalloidin-AlexaFluor488 and DAPI to visualize the typical OC actin rings (red) and the nuclei (blue), respectively; Magnification = 200×.

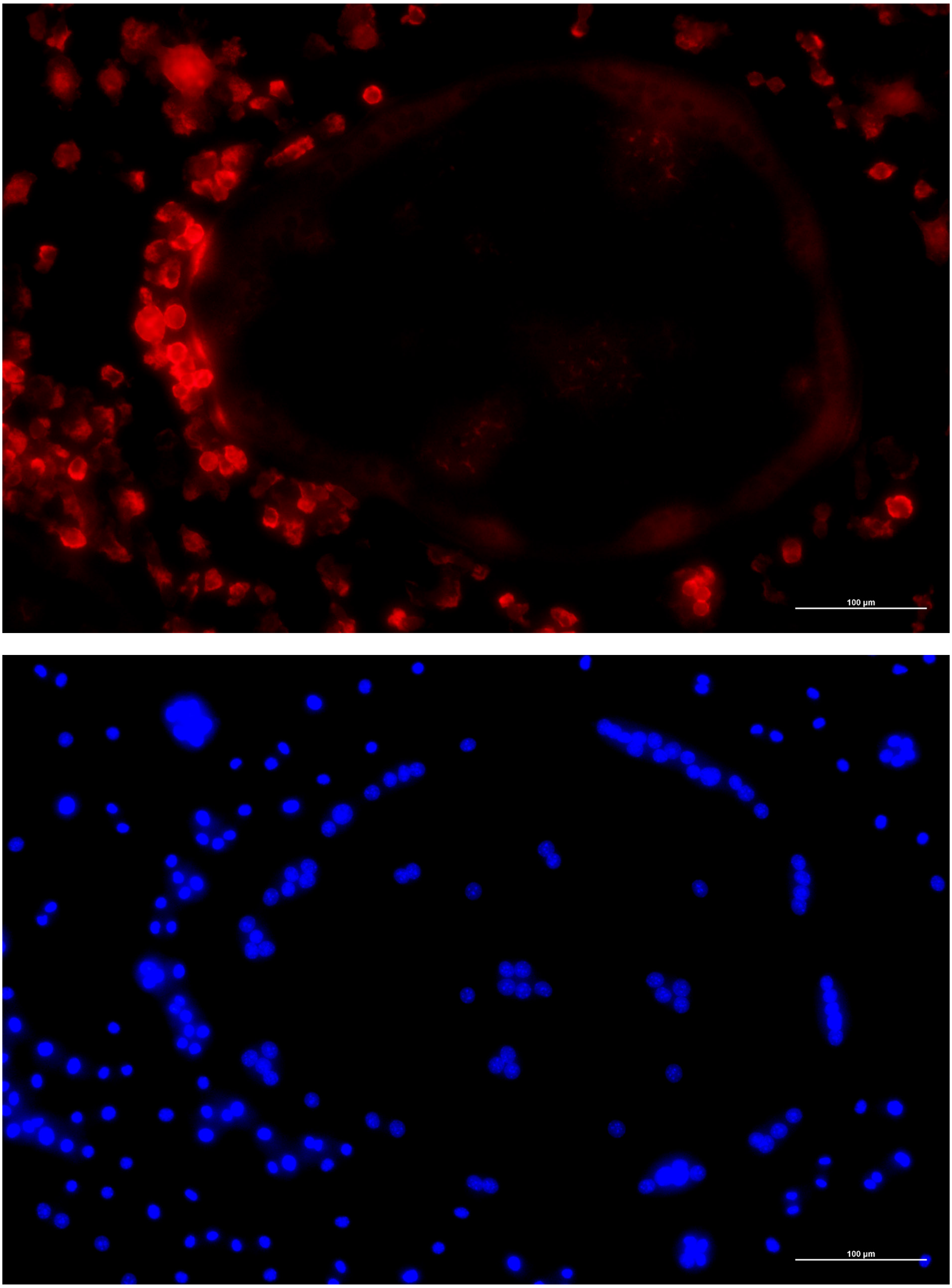

Figure S38. Immunostaining of the OCs co-cultured with 2 Gy-CM using phalloidin-AlexaFluor488 and DAPI to visualize the typical OC actin rings (red) and the nuclei (blue), respectively; Magnification = 200×.

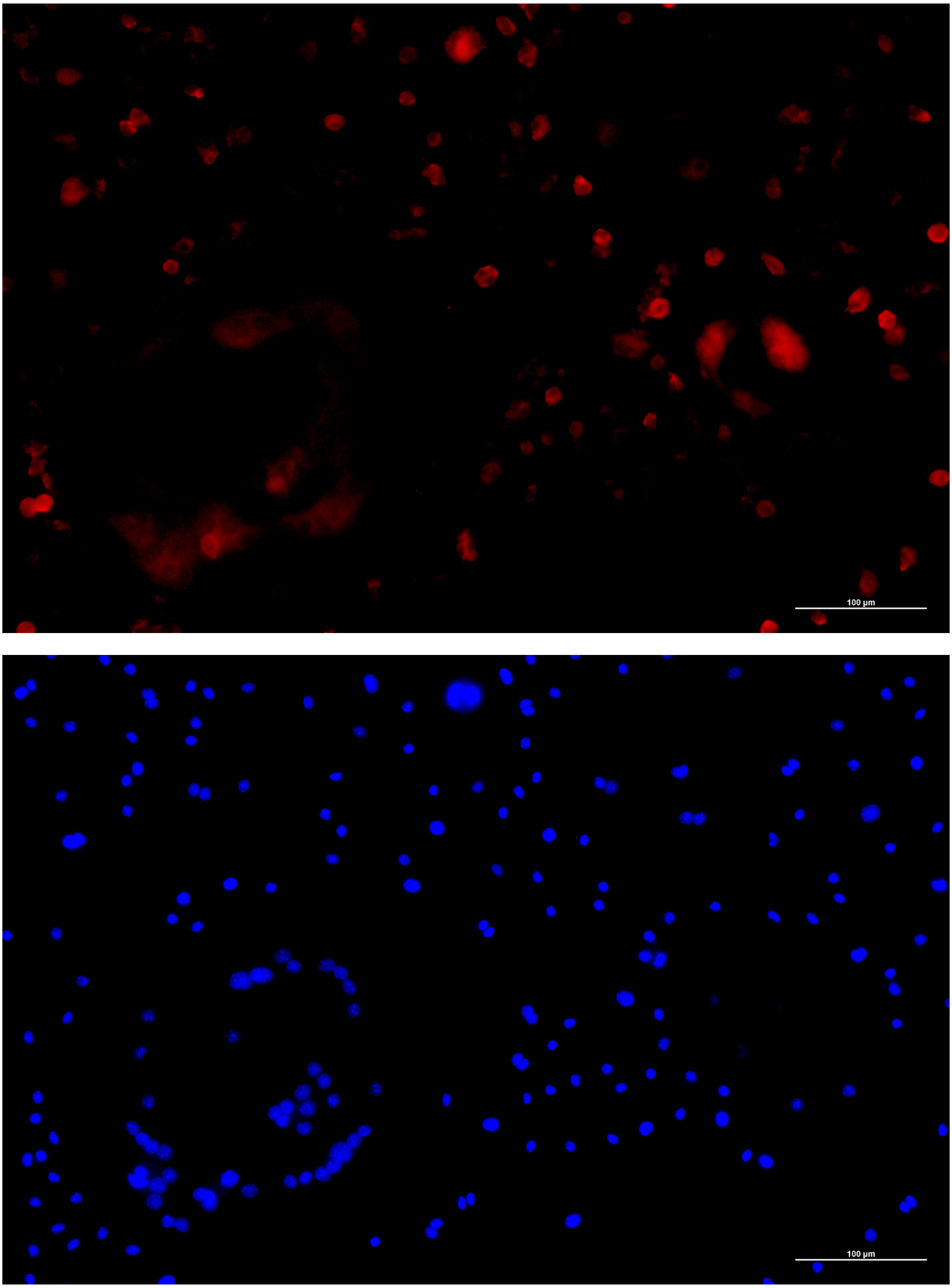

Figure S39. Immunostaining of the OCs co-cultured with 2 Gy-CM + anti-CCL3 using phalloidin-AlexaFluor488 and DAPI to visualize the typical OC actin rings (red) and the nuclei (blue), respectively; Magnification = 200×.

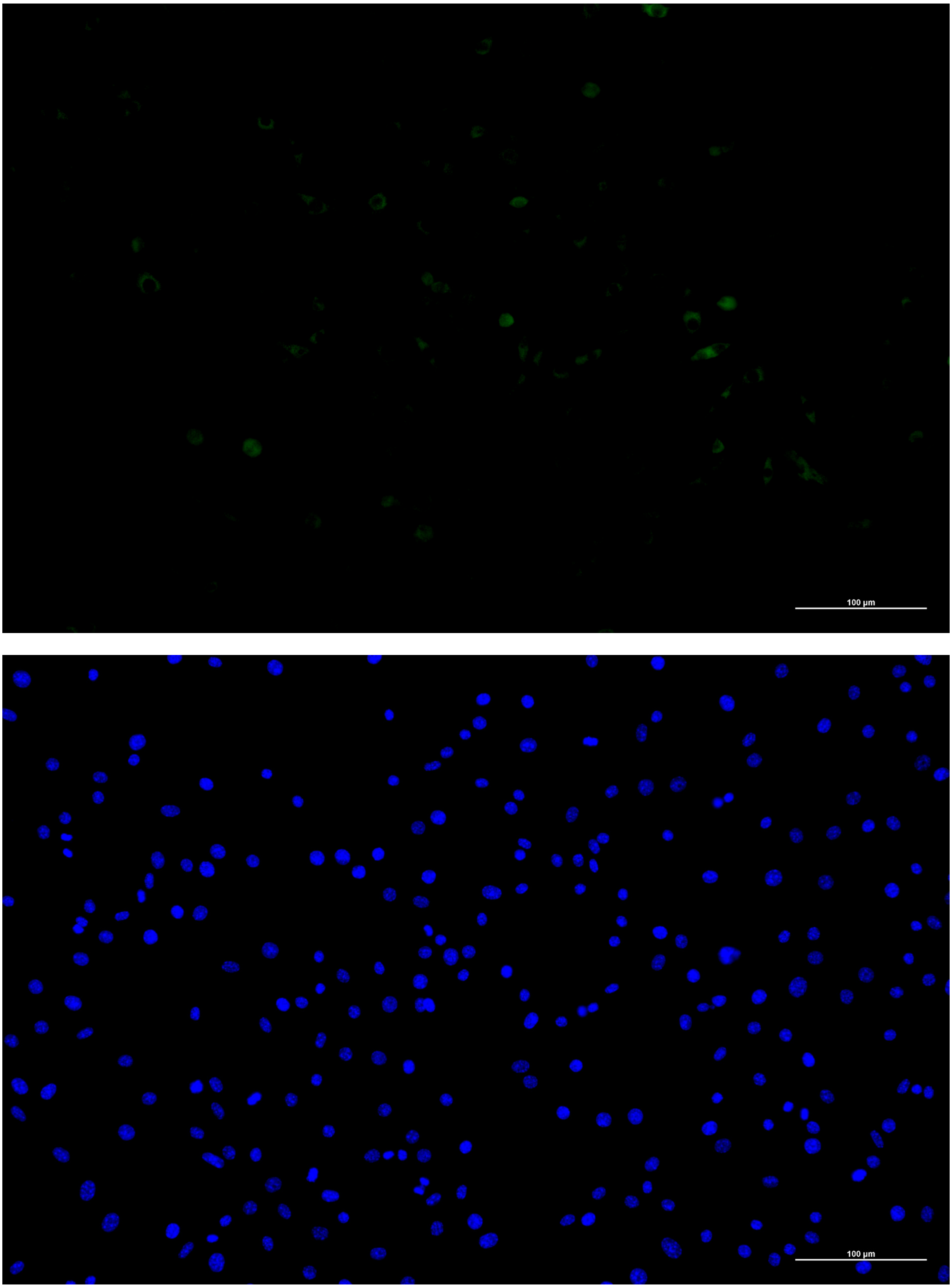

Figure S40. Immunofluorescence staining for nuclear translocation of p65 in primary OCYs: p65 (green) and DAPI (blue); Magnification = 200×.

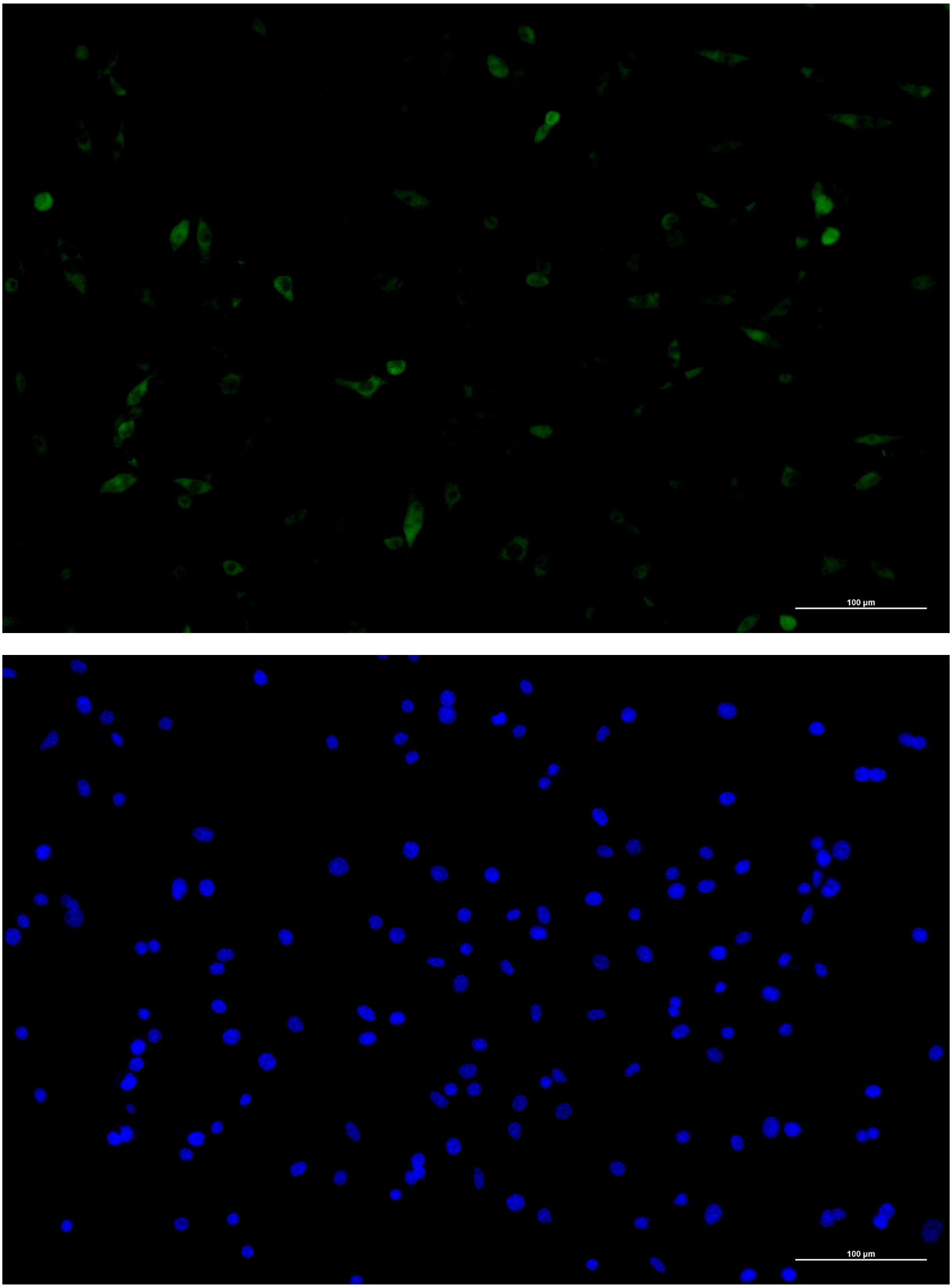

Figure S41. Immunofluorescence staining for nuclear translocation of p65 in irradiated OCYs: p65 (green) and DAPI (blue); Magnification = 200×.

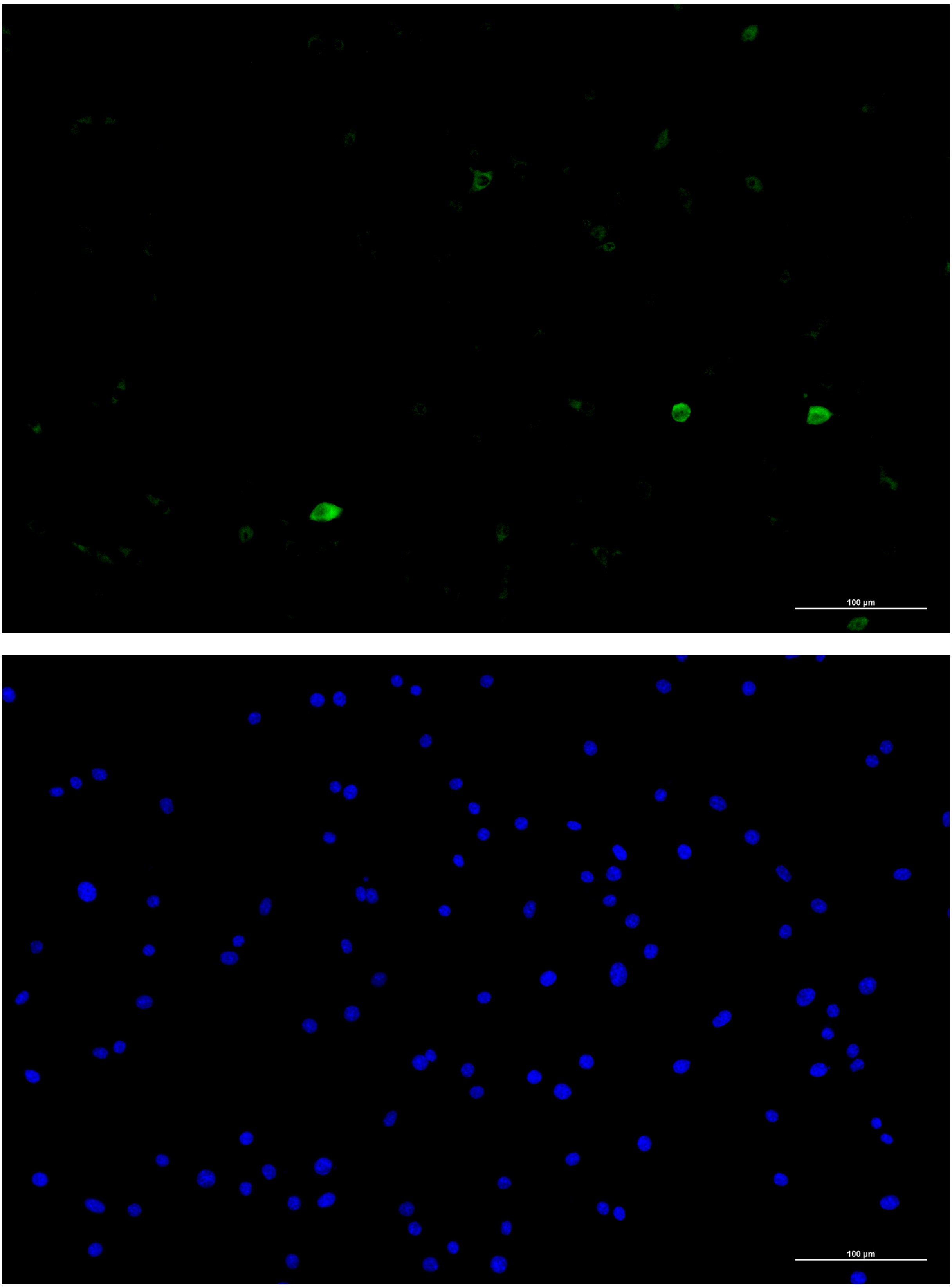

Figure S42. Immunofluorescence staining for nuclear translocation of p65 in irradiated OCYs treated with anti-CCL3: p65 (green) and DAPI (blue); Magnification = 200×.
